# Supplementary material for: Immigrant Older Adults’ Experiences of Aging in Place and Their Neighborhoods: A Qualitative Systematic Review
Source: Int J Environ Res Public Health. 2024 Jul 10;21(7):904. doi: 10.3390/ijerph21070904 (PMC11277252; doi:10.3390/ijerph21070904)
Supplement: Supplementary file 1 [file ijerph-21-00904-s001.zip › Supplemental File S1 - Full Search Strategy.pdf]

Supplemental File S1: Full search strategies for databases

Ovid MEDLINE(R) ALL <1946 to April 04, 2023>
Date of search: April 5, 2023

1 aged/ or "aged, 80 and over"/ 3442947
2 (elder* or geriatric* or gerontolog* or senior* or (old* adj2 (aged or adult* or individual* or patient* or men or man or women or woman or person* or people*)) or centenarian* or nonagenarian* or octogenarian* or septuagenarian* or sexagenarian* or dottering or decrepit or tottering or overaged or "oldest old").mp. 1056799
3 Geriatrics/ 31299
4 1 or 2 or 3 3992458
5 exp "Emigrants and Immigrants"/ or Refugees/ or (immigrant* or immigration or emigrant* or emigration or refugee* or asylum seeker* or asylee* or displaced person* or "incomer*" or "in comer*" or "new comer*" or newcomer* or migrant* or resettler*).mp. 98329
6 (((cultur* or ethnic* or linguistic* or language*) adj2 (divers* or differen* or varie* or variance* or assort* or mosaic or inclusiv*)) or pluralism or "cross cultural*").mp. 120707
7 (raciali?ed or non-white or race).mp. 143922
8 (diverse adj3 (population* or communit* or group*)).mp. 38955
9 (("non-western" or foreign) adj2 (cultur* or heritage* or ethnicit* or background*)).mp. 1214
10 ((asia* or africa* or "South America*" or "Central America*" or latin? or hispanic or black or "Middle East*" or muslim or islam* or foreign or "non-white") adj3 (origin? or originat* or background* or heritage or ethnicit*)).ab. 25280
11 ((china or Chinese or india* or Indonesia* or pakistan* or Bangladesh* or Japan* or philippin* or vietnam* or turkey or Turkish or iran* or Thai* or Myanmar or Burma or Burmese or korea* or iraq* or Afghanistan* or "Saudi Arabia*" or Uzbekistan* or Malaysia* or yemen or nepal* or "Sri lanka*" or kazakhstan* or syria* or cambodia* or jordan or Azerbaijan or "united Arab emirates" or UAE or Tajikistan* or Israel* or laos or Laotian or Leban* or Kyrgyzstan* or Turkmenistan* or singapor* or Oman or Palestin* or Kuwait* or Georgia* or Mongolia* or Armenia* or Qatar or Behrain or timor-leste or cyprus or Bhutan or Maldives or Brunei or Taiwan* or "hong kong" or macao) adj4 (origin? or originate* or heritage* or ethnicit* or background*)).ab. 21773
12 ((nigeria* or Ethiopia* or Egypt* or congo* or tanzania* or "South Africa*" or Kenya* or Uganda* or Algeria* or sudan* or Morocco or moroccan or Angola* or ghana* or madagascar or Cameroon or "cote d'ivoire" or niger or "burkina faso*" or mali or malian* or malawi* or zambia* or senegal* or chad or somalia* or Zimbabw* or guinea* or rwanda* or benin or Burundi or Tunisia* or togo or "Sierra Leone" or Libya* or Liberia* or Mauritania* or eritrea* or Namibia* or Gambia* or Botswana* or Gabon or Lesotho or "guinea-bissau" or Mauritius or

mauritian or eswatini or djibouti or Comoros or "Cabo Verde" or "sao tome & principe" or seychelles) adj4 (origin? or originate* or heritage* or ethnicit* or background*)).ab. 6248
13 ((Brazil* or Colombia* or Argentina* or peru* or venezuela* or chile* or ecuador* or Bolivia* or paraguay or uruguay or guyana* or suriname or Guiana* or "Falkland island*") adj4 (origin? or originate* or heritage* or ethnicit* or background*)).ab. 2184
14 ((Belize or "Costa Rica*" or "El Salvador*" or Guatemala* or honduras or honduran or nicaragua* or panama*) adj4 (origin? or originate* or heritage* or ethnicit* or background*)).ab. 180
15 ((mexico or mexican*) adj4 (origin? or originate* or heritage* or ethnicit*)).ab. 1535
16 ((French or Spanish or German or Russian or Slavic or dutch or Italian or Arabic or Chinese or mandarin or Hindi or Bangla or Bengali or Portuguese or polish or Japanese or Punjabi or Nepali or Kashmiri or Romanian or Ukrainian or Turkish or foreign or "non-english") adj4 speak*).ti,ab. 19644
17 or/5-16437809
18 4 and 17 79447
19 ((neighbo?rhood* or locale or locality or ((community or communities or residence*) adj2 (planned or urban* or suburban or pocket or historic* or retirement or gated or adult-only or adults-only or city or cities or town* or rural or village* or hamlet*)) or "physical environment*" or "built environment*" or "residential environment" or "residential integration" or "residential segregation" or "communit* infrastructure*") adj4 (experienc* or view* or attitude* or belief* or believ* or sentiment* or feel* or opinion* or judg* or perception* or perceiv* or reaction* or response* or story or stories or reflection or narrati* or voice* or values or perspective* or impression* or understand* or concept* or comprehend* or comprehension or meaning* or context* or belong* or "sense of")).mp. 6836
20 residence characteristics/ or exp neighborhood characteristics/ 38176
21 Independent Living/ 11014
22 ("ag?ing in place" or "ag?ing in communit*").mp. 1114
23 19 or 20 or 21 or 22 53512
24 18 and 23 2127

OVID Embase <1974 to 2023 April 04>
Date of search: April 5, 2023

1 exp aged/ 3563069
2 (elder* or geriatric* or gerontolog* or senior* or (old* adj2 (age* or adult* or individual* or patient* or men or man or women or woman or person* or people*)) or centenarian* or nonagenarian* or octogenarian* or septuagenarian* or sexagenarian* or dottering or decrepit or tottering or overaged or "oldest old").mp. 1783379
3 exp geriatrics/ 41332

4 gerontology/ 3438
5 1 or 2 or 3 or 4 4405736
6 exp migrant/ or exp forced migrant/ 48328
7 exp refugee/ 17143
8 (immigrant* or immigration or emigrant* or emigration or refugee* or asylum seeker* or asylee* or displaced person* or "incomer*" or "in comer*" or "new comer*" or newcomer* or migrant* or resettler*).mp. 99120
9 (((cultur* or ethnic* or linguistic* or language*) adj2 (divers* or differen* or varie* or variance* or assort* or mosaic or inclusiv*)) or pluralism or "cross cultural*").mp. 149507
10 (raciali?ed or non-white or race).mp. 274134
11 (diverse adj3 (population* or communit* or group*)).mp. 49703
12 (("non-western" or foreign) adj2 (cultur* or heritage* or ethnicit* or background*)).mp. 1526
13 (("non-western" or foreign) adj2 (cultur* or heritage* or ethnicit* or background*)).mp. 1526
14 ((asia* or africa* or "South America*" or "Central America*" or latin? or hispanic or black or "Middle East*" or muslim or islam* or foreign or "non-white") adj3 (origin? or originat* or background* or heritage or ethnicit*)).ab. 38591
15 ((china or Chinese or india* or Indonesia* or pakistan* or Bangladesh* or Japan* or philippin* or vietnam* or turkey or Turkish or iran* or Thai* or Myanmar or Burma or Burmese or korea* or iraq* or Afghanistan* or "Saudi Arabia*" or Uzbekistan* or Malaysia* or yemen or nepal* or "Sri lanka*" or kazakhstan* or syria* or cambodia* or jordan or Azerbaijan or "united Arab emirates" or UAE or Tajikistan* or Israel* or laos or Laotian or Leban* or Kyrgyzstan* or Turkmenistan* or singapor* or Oman or Palestin* or Kuwait* or Georgia* or Mongolia* or Armenia* or Qatar or Behrain or timor-leste or cyprus or Bhutan or Maldives or Brunei or Taiwan* or "hong kong" or macao) adj4 (origin? or originate* or heritage* or ethnicit* or background*)).ab. 31565
16 ((nigeria* or Ethiopia* or Egypt* or congo* or tanzania* or "South Africa*" or Kenya* or Uganda* or Algeria* or sudan* or Morocco or moroccan or Angola* or ghana* or madagascar or Cameroon or "cote d'ivoire" or niger or "burkina faso*" or mali or malian* or malawi* or zambia* or senegal* or chad or somalia* or Zimbabw* or guinea* or rwanda* or benin or Burundi or Tunisia* or togo or "Sierra Leone" or Libya* or Liberia* or Mauritania* or eritrea* or Namibia* or Gambia* or Botswana* or Gabon or Lesotho or "guinea-bissau" or Mauritius or mauritian or eswatini or djibouti or Comoros or "Cabo Verde" or "sao tome & principe" or seychelles) adj4 (origin? or originate* or heritage* or ethnicit* or background*)).ab. 8257
17 ((Brazil* or Colombia* or Argentina* or peru* or venezuela* or chile* or ecuador* or Bolivia* or paraguay or uruguay or guyana* or suriname or Guiana* or "Falkland island*") adj4 (origin? or originate* or heritage* or ethnicit* or background*)).ab. 3169

18 ((Belize or "Costa Rica*" or "El Salvador*" or Guatemala* or honduras or honduran or nicaragua* or panama*) adj4 (origin? or originate* or heritage* or ethnicit* or background*)).ab. 282
19 ((mexico or mexican*) adj4 (origin? or originate* or heritage* or ethnicit*)).ab. 1740
20 ((French or Spanish or German or Russian or Slavic or dutch or Italian or Arabic or Chinese or mandarin or Hindi or Bangla or Bengali or Portuguese or polish or Japanese or Punjabi or Nepali or Kashmiri or Romanian or Ukrainian or Turkish or foreign or "non-english") adj4 speak*).ti,ab. 25109
21 or/6-20612813
22 5 and 21 110104
23 ((neighbo?rhood* or locale or locality or ((community or communities or residence*) adj2 (planned or urban* or suburban or pocket or historic* or retirement or gated or adult-only or adults-only or city or cities or town* or rural or village* or hamlet*)) or "physical environment*" or "built environment*" or "residential environment" or "residential integration" or "residential segregation" or "communit* infrastructure*") adj6 (experienc* or view* or attitude* or belief* or believ* or sentiment* or feel* or opinion* or judg* or perception* or perceiv* or reaction* or response* or story or stories or reflection or narrati* or voice* or values or perspective* or impression* or understand* or concept* or comprehend* or comprehension or meaning* or context* or belong* or "sense of")).mp. 10453
24 residence characteristics/ 1672
25 exp neighborhood/ 16206
26 independent living/ 7065
27 ("ag?ing in place" or "ag?ing in communit*").mp. 1213
28 or/23-27 33084
29 22 and 28 1252

OVID APA PsycInfo <1806 to April Week 1 2023>
Date of search: April 5, 2023

1 older adulthood/ or exp geriatrics/ or gerontology/ 36911
2 (elder* or geriatric* or gerontolog* or senior* or (old* adj2 (age* or adult* or individual* or patient* or men or man or women or woman or person* or people*)) or centenarian* or nonagenarian* or octogenarian* or septuagenarian* or sexagenarian* or dottering or decrepit or tottering or overaged or "oldest old").mp. 273272
3 1 or 2 273272
4 immigration/ or refugees/ 33088
5 (immigrant* or immigration or emigrant* or emigration or refugee* or asylum seeker* or asylee* or displaced person* or "incomer*" or "in comer*" or "new comer*" or newcomer* or migrant* or resettler*).mp. 61842

6 (((cultur* or ethnic* or linguistic* or language*) adj2 (divers* or differen* or varie* or variance* or assort* or mosaic or inclusiv*)) or pluralism or "cross cultural*").mp. 165327

7 (raciali?ed or non-white or race).mp. 87677

8 (diverse adj3 (population* or communit* or group*)).mp. 16467

9 (("non-western" or foreign) adj2 (cultur* or heritage* or ethnicit* or background*)).mp. 1667

10 ((asia* or africa* or "South America*" or "Central America*" or latin? or hispanic or black or "Middle East*" or muslim or islam* or foreign or "non-white") adj3 (origin? or originat* or background* or heritage or ethnicit*)).ab. 6892

11 ((china or Chinese or india* or Indonesia* or pakistan* or Bangladesh* or Japan* or philippin* or vietnam* or turkey or Turkish or iran* or Thai* or Myanmar or Burma or Burmese or korea* or iraq* or Afghanistan* or "Saudi Arabia*" or Uzbekistan* or Malaysia* or yemen or nepal* or "Sri lanka*" or kazakhstan* or syria* or cambodia* or jordan or Azerbaijan or "united Arab emirates" or UAE or Tajikistan* or Israel* or laos or Laotian or Leban* or Kyrgyzstan* or Turkmenistan* or singapor* or Oman or Palestin* or Kuwait* or Georgia* or Mongolia* or Armenia* or Qatar or Behrain or timor-leste or cyprus or Bhutan or Maldives or Brunei or Taiwan* or "hong kong" or macao) adj4 (origin? or originate* or heritage* or ethnicit* or background*)).ab. 4850

12 ((nigeria* or Ethiopia* or Egypt* or congo* or tanzania* or "South Africa*" or Kenya* or Uganda* or Algeria* or sudan* or Morocco or moroccan or Angola* or ghana* or madagascar or Cameroon or "cote d'ivoire" or niger or "burkina faso*" or mali or malian* or malawi* or zambia* or senegal* or chad or somalia* or Zimbabw* or guinea* or rwanda* or benin or Burundi or Tunisia* or togo or "Sierra Leone" or Libya* or Liberia* or Mauritania* or eritrea* or Namibia* or Gambia* or Botswana* or Gabon or Lesotho or "guinea-bissau" or Mauritius or mauritian or eswatini or djibouti or Comoros or "Cabo Verde" or "sao tome & principe" or seychelles) adj4 (origin? or originate* or heritage* or ethnicit* or background*)).ab. 724

13 ((Brazil* or Colombia* or Argentina* or peru* or venezuela* or chile* or ecuador* or Bolivia* or paraguay or uruguay or guyana* or suriname or Guiana* or "Falkland island*") adj4 (origin? or originate* or heritage* or ethnicit* or background*)).ab. 288

14 ((Belize or "Costa Rica*" or "El Salvador*" or Guatemala* or honduras or honduran or nicaragua* or panama*) adj4 (origin? or originate* or heritage* or ethnicit* or background*)).ab. 32

15 ((mexico or mexican*) adj4 (origin? or originate* or heritage* or ethnicit*)).ab. 1447

16 ((French or Spanish or German or Russian or Slavic or dutch or Italian or Arabic or Chinese or mandarin or Hindi or Bangla or Bengali or Portuguese or polish or Japanese or Punjabi or Nepali or Kashmiri or Romanian or Ukrainian or Turkish or foreign or "non-english") adj4 speak*).ti,ab. 19542

17 or/4-16312004

18 3 and 17 17022

19 ((neighbo?rhood* or locale or locality or ((community or communities or residence*) adj2 (planned or urban* or suburban or pocket or historic* or retirement or gated or adult-only or adults-only or city or cities or town* or rural or village* or hamlet*)) or "physical environment*" or "built environment*" or "residential environment" or "residential integration" or "residential segregation" or "communit* infrastructure*") adj4 (experienc* or view* or attitude* or belief* or believ* or sentiment* or feel* or opinion* or judg* or perception* or perceiv* or reaction* or response* or story or stories or reflection or narrati* or voice* or values or perspective* or impression* or understand* or concept* or comprehend* or comprehension or meaning* or context* or belong* or "sense of")).mp. 6894
20 neighborhoods/ 10059
21 aging in place/ 271
22 ("ag?ing in place" or "ag?ing in communit*").mp. 744
23 19 or 20 or 21 or 22 14528
24 18 and 23 244

CINAHL via EBSCOhost (1936 - Present)
Date of search: April 5, 2023

S1 (MH "Aged+") OR (MH "Aged, 80 and Over+") 947,521
S2 (elder* or geriatric* or gerontolog* or senior* or (old* N2 (age* or adult* or individual* or patient* or men or man or women or woman or person* or people*)) or centenarian* or nonagenarian* or octogenarian* or septuagenarian* or sexagenarian* or dottering or decrepit or tottering or overaged or "oldest old") 413,024
S3 (MH "Geriatrics+") 6,863
S4 S1 OR S2 OR S3 1,103,457
S5 (MH "Immigrants+") OR (MH "Refugees+") 26,179
S6 (MH "Emigration and Immigration") 7,569
S7 (immigrant* or immigration or emigrant* or emigration or refugee* or asylum seeker* or asylee* or displaced person* or "incomer*" or "in comer*" or "new comer*" or newcomer* or migrant* or resettler*)46,686
S8 (((cultur* or ethnic* or linguistic* or language*) N2 (divers* or differen* or varie* or variance* or assort* or mosaic or inclusiv*)) or pluralism or "cross cultural*") 54,322
S9 (raciali#ed or non-white or race) 79,722
S10 (diverse N3 (population* or communit* or group*)) 12,717
S11 (("non-western" or foreign) N2 (cultur* or heritage* or ethnicit* or background*))628
S12 ((asia* or africa* or "South America*" or "Central America*" or latin# or hispanic or black or "Middle East*" or muslim or islam* or foreign or "non-white") N3 (origin# or originat* or background* or heritage or ethnicit*)) 10,246

S13 ((china or Chinese or india* or Indonesia* or pakistan* or Bangladesh* or Japan* or philippin* or vietnam* or turkey or Turkish or iran* or Thai* or Myanmar or Burma or Burmese or korea* or iraq* or Afghanistan* or "Saudi Arabia*" or Uzbekistan* or Malaysia* or yemen or nepal* or "Sri lanka*" or kazakhstan* or syria* or cambodia* or jordan or Azerbaijan or "united Arab emirates" or UAE or Tajikistan* or Israel* or laos or Laotian or Leban* or Kyrgyzstan* or Turkmenistan* or singapor* or Oman or Palestin* or Kuwait* or Georgia* or Mongolia* or Armenia* or Qatar or Behrain or timor-leste or cyprus or Bhutan or Maldives or Brunei or Taiwan* or "hong kong" or macao) N4 (origin# or originate* or heritage* or ethnicit* or background*)) 8,569

S14 ((nigeria* or Ethiopia* or Egypt* or congo* or tanzania* or "South Africa*" or Kenya* or Uganda* or Algeria* or sudan* or Morocco or moroccan or Angola* or ghana* or madagascar or Cameroon or "cote d'ivoire" or niger or "burkina faso*" or mali or malian* or malawi* or zambia* or senegal* or chad or somalia* or Zimbabw* or guinea* or rwanda* or benin or Burundi or Tunisia* or togo or "Sierra Leone" or Libya* or Liberia* or Mauritania* or eritrea* or Namibia* or Gambia* or Botswana* or Gabon or Lesotho or "guinea-bissau" or Mauritius or mauritian or eswatini or djibouti or Comoros or "Cabo Verde" or "sao tome & principe" or seychelles) N4 (origin# or originate* or heritage* or ethnicit* or background*)) 2,278

S15 ((Brazil* or Colombia* or Argentina* or peru* or venezuela* or chile* or ecuador* or Bolivia* or paraguay or uruguay or guyana* or suriname or Guiana* or "Falkland island*") N4 (origin# or originate* or heritage* or ethnicit* or background*)) 626

S16 ((Belize or "Costa Rica*" or "El Salvador*" or Guatemala* or honduras or honduran or nicaragua* or panama*) N4 (origin# or originate* or heritage* or ethnicit* or background*)) 67

S17 ((mexico or mexican*) N4 (origin# or originate* or heritage* or ethnicit*)) 977

S18 ((French or Spanish or German or Russian or Slavic or dutch or Italian or Arabic or Chinese or mandarin or Hindi or Bangla or Bengali or Portuguese or polish or Japanese or Punjabi or Nepali or Kashmiri or Romanian or Ukrainian or Turkish or foreign or "non-english") N4 speak*) 9,447

S19 S5 OR S6 OR S7 OR S8 OR S9 OR S10 OR S11 OR S12 OR S13 OR S14 OR S15 OR S16 OR S17 OR S18 198,630

S20 S4 AND S19 40,542

S21 ((neighbo#rhood* or locale or locality or ((community or communities or residence*) N2 (planned or urban* or suburban or pocket or historic* or retirement or gated or adult-only or adults-only or city or cities or town* or rural or village* or hamlet*)) or "physical environment*" or "built environment*" or "residential environment" or "residential integration" or "residential segregation" or "communit* infrastructure*") N4 (experienc* or view* or attitude* or belief* or believ* or sentiment* or feel* or opinion* or judg* or perception* or perceiv* or reaction* or response* or story or stories or reflection or narrati* or voice* or values or perspective* or impression* or understand* or concept* or comprehend* or comprehension or meaning* or context* or belong* or "sense of")) 4,867

S22 (MH "Residence Characteristics") 14,970
S23 (MH "Neighborhood Characteristics") 428
S24 ("ag#ing in place" or "ag#ing in communit*") 952
S25 S21 OR S22 OR S23 OR S24 19,601
S26 S20 AND S25: Limit to academic journals 916

SocINDEX (inception - present) via EBSCOhost
Date of search: April 5, 2023

S1 (elder* or geriatric* or gerontolog* or senior* or (old* N2 (age* or adult* or individual* or patient* or men or man or women or woman or person* or people*)) or centenarian* or nonagenarian* or octogenarian* or septuagenarian* or sexagenarian* or dottering or decrepit or tottering or overaged or "oldest old") 121,398
S2 (immigrant* or immigration or emigrant* or emigration or refugee* or asylum seeker* or asylee* or displaced person* or "incomer*" or "in comer*" or "new comer*" or newcomer* or migrant* or resettler*)98,016
S3 (((cultur* or ethnic* or linguistic* or language*) N2 (divers* or differen* or varie* or variance* or assort* or mosaic or inclusiv*)) or pluralism or "cross cultural*") 60,718
S4 (raciali#ed or non-white or race) 86,770
S5 (diverse N3 (population* or communit* or group*)) 5,652
S6 (("non-western" or foreign) N2 (cultur* or heritage* or ethnicit* or background*))978
S7 ((asia* or africa* or "South America*" or "Central America*" or latin# or hispanic or black or "Middle East*" or muslim or islam* or foreign or "non-white") N3 (origin# or originat* or background* or heritage or ethnicit*)) 6,192
S8 ((china or Chinese or india* or Indonesia* or pakistan* or Bangladesh* or Japan* or philippin* or vietnam* or turkey or Turkish or iran* or Thai* or Myanmar or Burma or Burmese or korea* or iraq* or Afghanistan* or "Saudi Arabia*" or Uzbekistan* or Malaysia* or yemen or nepal* or "Sri lanka*" or kazakhstan* or syria* or cambodia* or jordan or Azerbaijan or "united Arab emirates" or UAE or Tajikistan* or Israel* or laos or Laotian or Leban* or Kyrgyzstan* or Turkmenistan* or singapor* or Oman or Palestin* or Kuwait* or Georgia* or Mongolia* or Armenia* or Qatar or Behrain or timor-leste or cyprus or Bhutan or Maldives or Brunei or Taiwan* or "hong kong" or macao) N4 (origin# or originate* or heritage* or ethnicit* or background*)) 5,051
S9 ((nigeria* or Ethiopia* or Egypt* or congo* or tanzania* or "South Africa*" or Kenya* or Uganda* or Algeria* or sudan* or Morocco or moroccan or Angola* or ghana* or madagascar or Cameroon or "cote d'ivoire" or niger or "burkina faso*" or mali or malian* or malawi* or zambia* or senegal* or chad or somalia* or Zimbabw* or guinea* or rwanda* or benin or Burundi or Tunisia* or togo or "Sierra Leone" or Libya* or Liberia* or Mauritania* or eritrea* or Namibia* or Gambia* or Botswana* or Gabon or Lesotho or "guinea-bissau" or Mauritius or

mauritian or eswatini or djibouti or Comoros or "Cabo Verde" or "sao tome & principe" or seychelles) N4 (origin# or originate* or heritage* or ethnicit* or background*)) 1,452

S10 ((Brazil* or Colombia* or Argentina* or peru* or venezuela* or chile* or ecuador* or Bolivia* or paraguay or uruguay or guyana* or suriname or Guiana* or "Falkland island*") N4 (origin# or originate* or heritage* or ethnicit* or background*)) 523

S11 ((Belize or "Costa Rica*" or "El Salvador*" or Guatemala* or honduras or honduran or nicaragua* or panama*) N4 (origin# or originate* or heritage* or ethnicit* or background*)) 87

S12 ((mexico or mexican*) N4 (origin# or originate* or heritage* or ethnicit*)) 1,095

S13 ((French or Spanish or German or Russian or Slavic or dutch or Italian or Arabic or Chinese or mandarin or Hindi or Bangla or Bengali or Portuguese or polish or Japanese or Punjabi or Nepali or Kashmiri or Romanian or Ukrainian or Turkish or foreign or "non-english") N4 speak*) 5,293

S14 S2 OR S3 OR S4 OR S5 OR S6 OR S7 OR S8 OR S9 OR S10 OR S11 OR S12 OR S13 242,120

S15 S1 AND S14 11,502

S16 ((neighbo#rhood* or locale or locality or ((community or communities or residence*) N2 (planned or urban* or suburban or pocket or historic* or retirement or gated or adult-only or adults-only or city or cities or town* or rural or village* or hamlet*)) or "physical environment*" or "built environment*" or "residential environment" or "residential integration" or "residential segregation" or "communit* infrastructure*") N4 (experienc* or view* or attitude* or belief* or believ* or sentiment* or feel* or opinion* or judg* or perception* or perceiv* or reaction* or response* or story or stories or reflection or narrati* or voice* or values or perspective* or impression* or understand* or concept* or comprehend* or comprehension or meaning* or context* or belong* or "sense of")) 6,873

S17 ("ag#ing in place" or "ag#ing in communit*") 744

S18 S16 OR S17 7,573

S19 S15 AND S18: Limit to academic journals 142

Scopus (1976 - Present)
Date of search: April 5, 2023

(TITLE-ABS-KEY ((elder* OR geriatric* OR gerontolog* OR senior* OR (old* W/2 (age* OR adult* OR individual* OR patient* OR men OR man OR women OR woman OR person* OR people*)) OR centenarian* OR nonagenarian* OR octogenarian* OR septuagenarian* OR sexagenarian* OR dottering OR decrepit OR tottering OR overaged OR "oldest old"))) AND (TITLE-ABS-KEY ((immigrant* OR immigration OR emigrant* OR emigration OR refugee* OR asylum AND seeker* OR asylee* OR displaced AND person* OR "incomer*" OR "in comer*" OR "new comer*" OR newcomer* OR migrant* OR resettler*)) OR TITLE-ABS-

KEY ((((cultur* OR ethnic* OR linguistic* OR language*) W/2 (divers* OR differen* OR varie* OR variance* OR assort* OR mosaic OR inclusiv*)) OR pluralism OR "cross cultural*")) OR TITLE-ABS-KEY ((raciali?ed OR non-white OR race)) OR TITLE-ABS-KEY ((diverse W/3 (population* OR communit* OR group*))) OR TITLE-ABS-KEY ((("non-western" OR foreign) W/2 (cultur* OR heritage* OR ethnicit* OR background*))) OR TITLE-ABS-KEY (((asia* OR africa* OR "South America*" OR "Central America*" OR latin? OR hispanic OR black OR "Middle East*" OR muslim OR islam* OR foreign OR "non-white") W/3 (origin? OR originat* OR background* OR heritage OR ethnicit*))) OR TITLE-ABS-KEY (((french OR spanish OR german OR russian OR slavic OR dutch OR italian OR arabic OR chinese OR mandarin OR hindi OR bangla OR bengali OR portuguese OR polish OR japanese OR punjabi OR nepali OR kashmiri OR romanian OR ukrainian OR turkish OR foreign OR "non-english") W/4 speak*))) AND ((TITLE-ABS-KEY (((neighbo?rhood* OR locale OR locality OR ((community OR communities OR residence*) W/2 (planned OR urban* OR suburban OR pocket OR historic* OR retirement OR gated OR adult-only OR adults-only OR city OR cities OR town* OR rural OR village* OR hamlet*)) OR "physical environment*" OR "built environment*" OR "residential environment" OR "residential integration" OR "residential segregation" OR "communit* infrastructure*") W/4 (experienc* OR view* OR attitude* OR belief* OR believ* OR sentiment* OR feel* OR opinion* OR judg* OR perception* OR perceiv* OR reaction* OR response* OR story OR stories OR reflection OR narrati* OR voice* OR values OR perspective* OR impression* OR understand* OR concept* OR comprehend* OR comprehension OR meaning* OR context* OR belong* OR "sense of")))) OR (TITLE-ABS-KEY (("ag?ing in place" OR "ag?ing in communit*"))))) AND (LIMIT-TO (DOCTYPE , "ar") OR LIMIT-TO (DOCTYPE , "re"))
Results: 75

Cochrane Library (1993 - present) via Wiley
Date of search: April 5, 2023

ID Search Hits
#1 [mh ^aged] or [mh ^"aged, 80 and over"] 254817
#2 (elder* or geriatric* or gerontolog* or senior* or (old* NEAR/2 (aged or adult* or individual* or patient* or men or man or women or woman or person* or people*)) or centenarian* or nonagenarian* or octogenarian* or septuagenarian* or sexagenarian* or dottering or decrepit or tottering or overaged or "oldest old"):ti,ab,kw 100413
#3 [mh ^geriatrics] 397
#4 #1 or #2 or #3 322050
#5 [mh "emigrants and immigrants"] or [mh ^refugees] 471

#6 (immigrant* or immigration or emigrant* or emigration or refugee* or asylum seeker* or asylee* or displaced person* or "incomer*" or "in comer*" or "new comer*" or newcomer* or migrant* or resettler*):ti,ab,kw 2208
#7 (((cultur* or ethnic* or linguistic* or language*) NEAR/2 (divers* or differen* or varie* or variance* or assort* or mosaic or inclusiv*)) or pluralism or "cross cultural*"):ti,ab,kw 3892
#8 (raciali?ed or non-white or race):ti,ab,kw 12251
#9 (diverse NEAR/3 (population* or communit* or group*)):ti,ab,kw 1934
#10 (("non-western" or foreign) NEAR/2 (cultur* or heritage* or ethnicit* or background*)):ti,ab,kw 53
#11 ((asia* or africa* or "South America*" or "Central America*" or latin? or hispanic or black or "Middle East*" or muslim or islam* or foreign or "non-white") NEAR/3 (origin? or originat* or background* or heritage or ethnicit*)):ab 1745
#12 ((china or Chinese or india* or Indonesia* or pakistan* or Bangladesh* or Japan* or philippin* or vietnam* or turkey or Turkish or iran* or Thai* or Myanmar or Burma or Burmese or korea* or iraq* or Afghanistan* or "Saudi Arabia*" or Uzbekistan* or Malaysia* or yemen or nepal* or "Sri lanka*" or kazakhstan* or syria* or cambodia* or jordan or Azerbaijan or "united Arab emirates" or UAE or Tajikistan* or Israel* or laos or Laotian or Leban* or Kyrgyzstan* or Turkmenistan* or singapor* or Oman or Palestin* or Kuwait* or Georgia* or Mongolia* or Armenia* or Qatar or Behrain or timor-leste or cyprus or Bhutan or Maldives or Brunei or Taiwan* or "hong kong" or macao) NEAR/4 (origin? or originate* or heritage* or ethnicit* or background*)):ab 1475
#13 ((nigeria* or Ethiopia* or Egypt* or congo* or tanzania* or "South Africa*" or Kenya* or Uganda* or Algeria* or sudan* or Morocco or moroccan or Angola* or ghana* or madagascar or Cameroon or "cote d'ivoire" or niger or "burkina faso*" or mali or malian* or malawi* or zambia* or senegal* or chad or somalia* or Zimbabw* or guinea* or rwanda* or benin or Burundi or Tunisia* or togo or "Sierra Leone" or Libya* or Liberia* or Mauritania* or eritrea* or Namibia* or Gambia* or Botswana* or Gabon or Lesotho or "guinea-bissau" or Mauritius or mauritian or eswatini or djibouti or Comoros or "Cabo Verde" or "sao tome & principe" or seychelles) NEAR/4 (origin? or originate* or heritage* or ethnicit* or background*)):ab 324
#14 ((Brazil* or Colombia* or Argentina* or peru* or venezuela* or chile* or ecuador* or Bolivia* or paraguay or uruguay or guyana* or suriname or Guiana* or "Falkland island*") NEAR/4 (origin? or originate* or heritage* or ethnicit* or background*)):ab 63
#15 ((Belize or "Costa Rica*" or "El Salvador*" or Guatemala* or honduras or honduran or nicaragua* or panama*) NEAR/4 (origin? or originate* or heritage* or ethnicit* or background*)):ab 9
#16 ((mexico or mexican*) NEAR/4 (origin? or originate* or heritage* or ethnicit*)):ab 69
#17 ((French or Spanish or German or Russian or Slavic or dutch or Italian or Arabic or Chinese or mandarin or Hindi or Bangla or Bengali or Portuguese or polish or Japanese or

Punjabi or Nepali or Kashmiri or Romanian or Ukrainian or Turkish or foreign or "non-english") NEAR/4 speak*):ti,ab,kw 2511
#18 {or #5-#17} 24156
#19 #4 and #18 4360
#20 ((neighbo?rhood* or locale or locality or ((community or communities or residence*) NEAR/2 (planned or urban* or suburban or pocket or historic* or retirement or gated or adult-only or adults-only or city or cities or town* or rural or village* or hamlet*)) or "physical environment*" or "built environment*" or "residential environment" or "residential integration" or "residential segregation" or "communit* infrastructure*") NEAR/4 (experienc* or view* or attitude* or belief* or believ* or sentiment* or feel* or opinion* or judg* or perception* or perceiv* or reaction* or response* or story or stories or reflection or narrati* or voice* or values or perspective* or impression* or understand* or concept* or comprehend* or comprehension or meaning* or context* or belong* or "sense of")):ti,ab,kw 230
#21 [mh ^"residence characteristics"] or [mh ^"neighborhood characteristics"] 830
#22 [mh ^"independent living"] 942
#23 ("aging in place" or "aging in communit*"):ti,ab,kw 35
#24 #20 or #21 or #22 or #23 1962
#25 #19 and #24 64

Sociological Abstracts (1952 - present) via ProQuest
Date of search: April 5, 2023

S1 noft((elder* or geriatric* or gerontolog* or senior* or (old* NEAR/2 (age* or adult* or individual* or patient* or men or man or women or woman or person* or people*)) or centenarian* or nonagenarian* or octogenarian* or septuagenarian* or sexagenarian* or dottering or decrepit or tottering or overaged or "oldest old"))
S2 noft((immigrant* or immigration or emigrant* or emigration or refugee* or asylum seeker* or asylee* or displaced person* or "incomer*" or "in comer*" or "new comer*" or newcomer* or migrant* or resettler*)) OR noft((((cultur* or ethnic* or linguistic* or language*) NEAR/2 (divers* or differen* or varie* or variance* or assort* or mosaic or inclusiv*)) or pluralism or "cross cultural*")) AND noft((raciali#ed or non-white or race))
S3 noft(((neighbo#rhood* or locale or locality or ((community or communities or residence*) NEAR/2 (planned or urban* or suburban or pocket or historic* or retirement or gated or adult-only or adults-only or city or cities or town* or rural or village* or hamlet*)) or "physical environment*" or "built environment*" or "residential environment" or "residential integration" or "residential segregation" or "communit* infrastructure*") NEAR/4 (experienc* or view* or attitude* or belief* or believ* or sentiment* or feel* or opinion* or judg* or perception* or perceiv* or reaction* or response* or story or stories or reflection or narrati* or voice* or values or perspective* or impression* or understand* or concept* or comprehend* or

comprehension or meaning* or context* or belong* or "sense of"))) OR noft(("ag#ing in place" or "ag#ing in communit*"))
S4 S1 and S2 and S3
Results: 12
