# Supplementary material for: Immigrant Older Adults’ Experiences of Aging in Place and Their Neighborhoods: A Qualitative Systematic Review
Source: Int J Environ Res Public Health. 2024 Jul 10;21(7):904. doi: 10.3390/ijerph21070904 (PMC11277252; doi:10.3390/ijerph21070904)
Supplement: Supplementary file 1 [file ijerph-21-00904-s001.zip › Supplemental File S3 - Full Meta-Aggregation Table.pdf]

Supplemental File S3 – Full Meta-Aggregation Table

Findings

Walkability can be improved by enhancing safety features of neighbourhoods to address physical disability limitations that are exacerbated by winter weather conditions. Preferences about living in areas with warmer climate, clean spaces, and accessible greenspaces in the neighbourhood were important factors for older immigrants to feel comfortable frequenting areas around their home.

1.1 Physical accessibility and walkability

Author Statement	Illustration
the challenges older people faced navigating their local neighborhoods, bringing the physicality of places to the fore. This was particularly salient in Yorkshire where we observed how older people struggled with the hilly terrain. (p.837) [Lorinc 2022; U]	Millicent (83) remarked that the biggest improvement to the area would be to “flatten it” as she lived “on top of a hill.” We accompanied her on a bus ride and a short walk home from a shopping trip from the city center. During this journey, Millicent explained the various environmental challenges she faced when conducting her everyday mobilities, including the long steep hill and steps leading to her house, potholes and uneven surfaces, overhanging vegetation blocking the footpath, rubbish on the street, and having to change buses several times to reach her destination. (p.837-8) [Lorinc 2022; U]
environmental factors that aided her mobility, such as having access to good transport links close to her house and the handrail next to the steps (p.838) [Lorinc 2022; U]	“in the ice and the frost, you want something to hold onto.” (p.838) [Lorinc 2022; U]
The findings also highlighted barriers linked to the urban deprivation in the area, including the physical deterioration of infrastructure and the lack of outdoor seating and pedestrian safety, which prevented participants from moving between their homes, neighbourhoods, and public spaces… Such pressures may present particular challenges to	The roads are dirty and full of cigarette butts and cans. They throw everything on the floor. Despite cleaning our front door, they always make it filthy it again… And pubs are open all night round here, until the morning hours. They make a lot of noise. But where can we make a complaint? The municipality never does anything. There is no order and hygiene

maintaining a sense of home, especially when their person's capabilities to deal with these are restricted.<br>(p08) [Buffel 2017; U]	anymore in this neighbourhood' (64-year-old woman).(p.08)<br>**The pavements and streets are very narrow and dangerous. People get hit by cars when they try to cross the road' (71-year-old man).(p.09)**<br><br>**'The biggest problem in this area is the busy traffic and the fact that there are too many cars… There are no benches and there is hardly any green space around here' (66-year-old woman).(p.09) [Buffel 2017; U]**
Participants from London tended to have better access to transport, shops, and services; however, the traffic, noise, and agglomeration on the streets caused difficulties for them.<br>(p.838) [Lorinc 2022; C]	"On a Saturday I wouldn't venture out because it's too much ... too crowded for me," (p.838) [Lorinc 2022; C]
Older people who were already disadvantaged in terms of ill-health were especially vulnerable to such structural barriers. For example, some lived in houses that were poorly adapted for people with physical disabilities, and for those who lived in high-rise buildings that lacked an elevator, their housing conditions prevented them from leaving their home. (p. 26)<br><br>Similar issues were reported among some participants in England (p.27) [Buffel 2011; U]	A Moroccan woman in Brussels who cared for her husband since he underwent an operation, for instance, reported that:He can't go outdoors anymore because we are on the fourth floor and he can't cope with the stairs. I stay with him… So no, we don't see many people, except from the ones that visit us.<br><br>An older Somali man commented on the problems he experienced in his home:The stairs are a particular problem for me. There are times when I try to take a cup of tea upstairs and it is difficult for me. Also I need a new heating system because it is very cold and I need a house which will be much warmer. [Buffel 2011; U]
Participants said that stepstool and assistance with walkers may facilitate the use of transportation services. [Dabelko-Schoeny 2021; U]	Number one, they have to have some kind of a stepstool to get in. If it's too high,they can't ... Then all the walkers,they were flying all over. There was a very small space for a person to just sit, and they just burst into tears because they don't ask what people need. (Russian)."<br>(p. 6) [Dabelko-Schoeny 2021; U]

In addition to providing resting places, benches provide opportunities to socialise with others and contributed to the feeling of inclusion:<br>… It supported older Surinamese people's ability to go for walks and engage in active lifestyles.” (p. 8) [Jagroep 2023; U]	Having benches is also inviting to take a walk and then enjoy the weather. Sometimes you get talking to others. (Participant 1) [Jagroep 2023; U]
The extent to which communities supported older adults' ability to go outside and engage with community life during the pandemic differed among neighbourhoods. [Jagroep 2023; U]	Some interviewees indicated that they went for walks less often during the pandemic due to the lack of resting places, as public benches in their neighbourhoods had been removed or made unavailable. However, other interviewees indicated that benches with 1.5 m spacing of seats, according to the social distancing rule, were available in their neighbourhoods. (p. 8) [Jagroep 2023; U]
Older Surinamese adults valued having various destinations (e.g. different supermarkets, pharmacies, libraries, cafe ́s) at walking distance in their neighbourhoods, as they made going for walks appealing. They also noted that features such as place to rest and public toilets supported them going out and engagement with the community. [Jagroep 2023; C]	They reported that the closure of public toilets during the COVID-19 pandemic reduced their confidence and discouraged them from going outside, and indeed that they had spent less time outside during the pandemic than previously. They emphasised the importance of keeping moving, for their general health and especially when recommended by physiotherapists. (p. 9) [Jagroep 2023; C]

1.2 Winter weather challenges

Author Statement	Illustration
Weather and topography could be place-specific factors. [Ryan 2021; U]	Henrietta, a 79-year-old Jamaican-born woman, lived in a particularly hilly area in Yorkshire, noted the additional efforts required to navigate her neighbourhood in winter: ‘when the snow [comes] … we cannot come up and they can't come down, so you get stuck in the middle of the road. But still I move about because I can go two ways instead of coming down’. (p.06) [Ryan 2021; U]

Among immigrant older adults, walking was identified as a popular way of getting around in their respective countries of origin. However, participants walked less in their current community in the U.S. due to the lack of sidewalks, winter conditions, unsafe neighborhoods, and heavy traffic. Walkability of neighborhoods varied by geographic region. [Dabelko-Schoeny 2021; U]	They say because the winter was always good (in Somalia) they used to walk. They used to walk for local stuff that they can go. They say walking was healthy for us. It was good, but here we cannot walk. (Somali). (p. 8) [Dabelko-Schoeny 2021; U]
Samantha described having the biggest struggle during winter. [Schuster 2019; U]	She said that when the sidewalks were snowy and icy, she was too afraid to leave the house. On the one side, Samantha worried about becoming a burden to her family by hurting herself and the family having to take care of her. On the other side, she was not happy about making them drive her around. But being stuck in the house frustrated her: "Winter is hard for me. I must stay inside the house. And I cannot go outside. [...] I even stopped taking the kids to school. [...] I live almost every day just at home. Just at home. And it's very boring." (p. 58) [Schuster 2019; U]
All four participants were scared about falling on icy pathways, getting hurt, and then becoming a greater burden to their families. Therefore, they spent their days in their homes. [Schuster 2019; U]	Especially Samantha was frustrated about this situation, as being outside allowed her to socialize with people and being locked in the house made her feel isolated. Elena saw the winter as an opportunity to focus more on her self-care and exercise in the house rather than go outside to take walks or drive the car to pick up her grandchildren. For Oana, walking outside in the winter became a bigger issue after she was not able to stand and walk properly anymore after her health declined. "I must stay inside the house and I cannot go outside. [...] Because I can't at that time. I'm afraid I will have a fall and break a bone or something like that. [...] I live almost every day just at home, just at home... and it's very boring." (Samantha) "But when there is snow, I don't like to go out because I don't want to fall down. [...] I walk more on the treadmill."(Elena) "We caught some winters

	which were very difficult. Now I have problems going outside in the winter." (Oana)" (p. 82) [Schuster 2019; U]
Outside of their child's home, participants rarely engaged in their neighborhoods and communities. Sponsoring children shouldered the financial care and fulfilled community occupations on participants' behalf. Thus, outside of healthcare facilities, the Buddhist temple, and infrequent trips to a mall, post-office, or library, participants rarely engaged in their community. The lack of diversity of occupational spaces meant that participants social encounters were limited to their families, members of the ethnic community, healthcare providers, service providers (e.g., cashiers, pharmacists) and the occasional telemarketer. The limited interactions with the non-ethnic community did not meet the socialization needs of some participants, particularly males, who had previously enjoyed an extensive social life in Sri Lanka. [Wijekoon 2018; U]	I lived as a very sociable person there [in Sri Lanka]. [I had] a lot of connections. I would go to gathering places to engage in convivial socialization. I do not have the opportunity to continue that level of socialization here. I grieve that. It is during the winter that I feel very restricted, alone and isolated. I cannot stay in one place like this. – Bandula" (p. 165) [Wijekoon 2018; U]
Loneliness, isolation and sense of confinement reached its zenith during the winter. The winter, perceived as barren and unwelcoming, curtailed participants' outdoor mobility…<br>Moreover, features of the physical environment, including the desolation of neighbourhoods, particularly during the day time when people are at work and school, intensified participants' isolation and outsiderness in Canada." (p. 164) [Wijekoon 2018; U]	**From our house to the bus stop, you have to walk over ice. [I] have to wear three or four layers, wear gloves. This clothing is very heavy. [You] have to exert yourself. If the ice is frozen, then you could slip and fall. If I slip and fall, I will have to suffer. I will become a headache to my children. – Ranil [Wijekoon 2018; U]**
Surprisingly, the most frequently mentioned factor that makes older adults feel at home in Atlanta is the warm weather, listed before being with children. Thirteen out of 20 interviewees said that nice and warm weather came to the top of their list when choosing	Ms. Liao used to live in Boston. Upon retirement, she and her husband decided to move to Atlanta. When asked if her children are in Atlanta, she said.<br>"No, my children are not here, I have a daughter in Boston, she is my only child. I

Atlanta as their home to settle down in old age. [Zhan 2017; U]	came to Atlanta because my friend came here first and I visited them. I like the weather here, and the cost of living is low in Atlanta compared to Boston. So we moved to here and bought a house in Atlanta."<br>When asked if she would move back to Boston near her daughter when she was older and needed some help with daily living, she answered without hesitation, "No, Atlanta is my home now, I do not plan to move to Boston because it is cold there, very cold. I like to be warm. Even in the summer, my hands are cold. Also, my daughter has her life. I don't want to be in her way. I like it here very much. Atlanta is my home." [Zhan 2017; U]
Finding a place called home, based on the experiences of these Chinese seniors, appeared to have a lot to do with the weather, the natural and built environment, and the standard of living.<br>Similar to earlier studies weather is found to be an important factor in old age. Bad weather or winter seasons can be major confining factors for older adults.. This study also reveals that some seniors moved to the south for the warm weather to avoid cold winters like in Boston. In addition, seniors in this study echoed findings in earlier studies that emphasized positive emotions such as comfort, joy, and relaxation in their perceptions of making Atlanta their home. Clearly, the natural and built environment is directly linked to individual's emotional perception of the home. [Zhan 2017; U]	Beyond the warm weather, the natural built environment is another reason that adds to the Chinese seniors' sense of home. Ms. Qin described her sense of home in this way: "When I go out, I go inside the park, children's playing ground makes me very happy. I take a walk every day and enjoy the sunshine, which makes me feel good. Also, the library–when I was in better health, I used to borrow every kind of books to read. I also borrowed foreign DVD (meaning non-Chinese). So people in the library were very nice to me. There were Chinese books there too." [Zhan 2017; U]

1.3 Importance of Clean Spaces

Author Statement	Illustration

The findings also highlighted barriers linked to the urban deprivation in the area, including the physical deterioration of infrastructure and the lack of outdoor seating and pedestrian safety, which prevented participants from moving between their homes, neighbourhoods, and public spaces… Such pressures may present particular challenges to maintaining a sense of home, especially when their person's capabilities to deal with these are restricted.<br>(p08) [Buffel 2017; U]	**The roads are dirty and full of cigarette butts and cans. They throw everything on the floor. Despite cleaning our front door, they always make it filthy it again… And pubs are open all night round here, until the morning hours. They make a lot of noise. But where can we make a complaint? The municipality never does anything. There is no order and hygiene anymore in this neighbourhood' (64-year-old woman).(p.08)**<br>The pavements and streets are very narrow and dangerous. People get hit by cars when they try to cross the road' (71-year-old man).(p.09)<br><br>'The biggest problem in this area is the busy traffic and the fact that there are too many cars… There are no benches and there is hardly any green space around here' (66-year-old woman).(p.09) [Buffel 2017; U]
Cleanliness in public spaces was mentioned by both groups as an essential factor in their assessments of their communities. (p.23) [Luo 2016; U]	The individuals living in mainstream buildings seemed satisfied with the level of public hygiene maintenance, but those in the Chinese building stated they had concerns about the quality of cleaning, and they also commented on a few architectural and interior design issues, such as a lack of windows in the hallways that might allow fresh air to come in, and a lack of curtains on the existing windows. (p.23) [Luo 2016; U]

1.4 Availability of nearby greenspaces

Author Statement	Illustration
According to the data drawn from the travel diaries, nearly all (93%) the physical activities or exercises undertaken by this group were conducted in urban green spaces, while the remaining 7% of physical activities were	Fu reported: "I only do some simple exercises in the park near my home, such as walking or doing some stretching. Green spaces enable me to walk more. It's easy for me to walk 1 to 2 kilometers in the parks. It's good for our

conducted in community centers or churches. This result alludes to the essential role of green spaces, as these participants heavily rely on these spaces for their physical activities. [Gao 2020; U]	health. If I stay at home, I think I won't be able to go out anymore. That means my health may collapse. So, I go to the park every day." (p. 7) [Gao 2020; U]
The result indicates that green spaces offered a place for them to conduct activities independently [Gao 2020; U]	As Bei (female, 64 years old, living in Australia for 3 years) pointed out: "I can't drive or take buses here. The small park near my house is a place for me to visit independently. I can go to that small park at any time without asking my son to drive me there. I can enjoy time by myself." (p. 5) [Gao 2020; U]
The participants developed a sense of improved health when they visited green spaces, congruent with their values of maintaining health, and thus this also generated positive perceptions of green spaces. [Gao 2020; U]	As Hu (female, 70 years old, living in Australia for 9 years) pointed out: "I am happy to live here. The parks here make me feel close to nature. They provide an open space where I can sit and gaze into this landscape. I can feel the warm sunshine, see the beautiful scenery, and hear the birdsong, which helps me escape from annoyances. It is definitely good for my health!" (p. 7) [Gao 2020; U]
The high accessibility of green spaces encouraged the participants to visit these spaces and conduct more physical activities. [Gao 2020; U]	Yi (male, 71 years old, living in Australia for 1 year) noted: "The park is near my home, only 2 minutes' walk. It's really convenient. If I have time, I will go to that park and sit for a while." (p. 5) [Gao 2020; U]
They prioritized going to green spaces as a regular daily activity to spend leisure time and escape boredom. They perceived experiences with green spaces to be integral in experiencing a fulfilling day. [Gao 2020; U]	As Fu noted: "I go to the small park to walk and do some exercise twice a day. Once is in the morning and the other is after my dinner. In China, I have lots of physical or social activities, but in Australia I have no place to go. So, going to the nearby park has become a thing for me; otherwise, I have nothing to do and I am always at home." (p. 8) [Gao 2020; U]
"tell me what you like about Milwaukee and Wisconsin" the most common answer was the natural environment, specifically their neighborhoods, parks, and lakes:	"First and foremost – (Lake) Michigan" – Participant 4 (88 years old). She then sang a short song about Lake Michigan. michigan song.mp3

[Hawkins 2022; U]	Photos one and two – taken by participants one and two. The photos capture a park on the shores of Lake Michigan. “It is good because there is the lake. There is somewhere to go and look at the lake.” Participant 1 (81 years old”<br>“It is a good place...I feel invigorated here, you can go nearby and rest, your eyes will rest, and your soul will rest in the fresh air.” – Participant 2 (75 years old).” (p. 110)[Hawkins 2022; U]
Participants discussed their love of both wild animals in parks and their own pets as positive experiences in their lives. Animals, both wild and domestic, provided a sense of both enjoyment and comfort to participants. [Hawkins 2022; U]	“Photos three and four were taken by participant 8 (71 years old). She reiterated her love of the lake and parks particularly her daily walks in those parks, as well as her enjoyment of the animals…<br>“I really like this beauty...When I’m going home [to my apartment], this is [the beauty] I see [showing the photos]... I love them (the birds), I don’t know why... Once I’m back home, I’ll show my neighbors.”– Participant 8 (71 years old). Participant 8 also noted that seeing the animals reminded her of home: “here there are plenty (of animals), there (in Azerbaijan) there are a lot (of animals).”– Participant 8” (, p. 110) [Hawkins 2022; U]
Health promotive occupations, for the upkeep of the physical body and cultivation of the aging mind, were discussed primarily as ways to ensure the maintenance of independence, particularly in self-care, so as to lessen the undue and avoidable burden placed on the sponsoring child. To maintain their mental and physical health, participants resorted to familiar health promotive occupations. They went for walks within the home, and in their neighborhoods when weather permitted. p. 106) [Wijekoon 2018; C]	Many participants enthusiastically participated in the monthly exercise program facilitated by a seniors’ group held at the Buddhist temples. Involvement in household responsibilities within their sponsored child’s home was reported to actively engage the mind and body. Others maintained a nutritious diet and abstained from foods, such as meat and alcohol, deemed unhealthy or immoral. Convinced of its potency and effectiveness, participants continued the use of Ayurveda, an ancient Indian system of healing, albeit at odd with their dubious westernized children. (p. 106) [Wijekoon 2018; C]

Additional participants noted how the lakes and parks reminded them of home, their place of origin. [Hawkins 2022; U]	**Participant 15 (61 years old) said: "I love the beach...I am from Puerto Rico, surrounded by water...I love the water. Here I love to go to the water, the water by Downtown." Participant 15 selected a picture of the beach in Puerto Rico (photo five). Participant 21 (80 years old) took photo six: "I like where we live. Because it's very tranquil, very beautiful. Not many cars pass by, it's good, good, very beautiful." As you can see in her photo, there a few cars, ample walking area, and plenty of green (in the summer, the photo was taken in February)" (p. 111) [Hawkins 2022; U]**
The findings also highlighted barriers linked to the urban deprivation in the area, including the physical deterioration of infrastructure and the lack of outdoor seating and pedestrian safety, which prevented participants from moving between their homes, neighbourhoods, and public spaces… Such pressures may present particular challenges to maintaining a sense of home, especially when their person's capabilities to deal with these are restricted.<br>(p08) [Buffel 2017; U]	The roads are dirty and full of cigarette butts and cans. They throw everything on the floor. Despite cleaning our front door, they always make it filthy it again… And pubs are open all night round here, until the morning hours. They make a lot of noise. But where can we make a complaint? The municipality never does anything. There is no order and hygiene anymore in this neighbourhood' (64-year-old woman).(p.08)<br>The pavements and streets are very narrow and dangerous. People get hit by cars when they try to cross the road' (71-year-old man).(p.09)<br><br>'The biggest problem in this area is the busy traffic and the fact that there are too many cars… There are no benches and there is hardly any green space around here' (66-year-old woman).(p.09) [Buffel 2017; U]
More than one-half (57%) of the study participants reported that they could barely conduct the physical activities they preferred… Older Chinese immigrants had accessibility to visit green spaces near their homes. The fact that they were unable to visit the parks that they preferred negatively	For example, as Yue (female, 61 years old, living in Australia for 2 years) complained: "Actually, there is a park that has activities such as square dancing and Tai Chi. Many older Chinese people do these activities there. But I can't go there by myself. So, I can't join them." [Gao 2020; U]

influenced their perceptions on green spaces on the Gold Coast. (p. 6) [Gao 2020; U]	
Khmer cultural practices continue to dominate across most interactions within the village….Khmer families, such as Prahm's, reunited or newly formed, have striven to create a feeling of home, security, and meaning in Veluvanna Village. (Khmer community dominant rural village in the US) (p384) [Lewis 2009; U]	The architecture of the Buddhist temple, located in the center of the village, is decidedly Khmer style. Most homes have gardens where traditional Cambodian fruits and vegetables are grown. One family has dug deep pits for growing lotus, a flower with significant meaning in Cambodian culture, and other traditional aquatic plants. Herbs from Cambodia sprout under canopies of massive green leafy vegetables, squash, banana trees, and tomatoes. Fragrant flowers, purple and spiked, share space with lemongrass, several kinds of mint, basil, and many different herbs. The yard is filled with plants Mai, Prahm, and others had eaten and used as medicine during the "Pol Pot time." Plants for food or for medicine grow rampantly thanks to the rich fertile soil, the blazing coastal heat, and the near-daily afternoon showers. Family, friends, and neighbors greet one another as they move about the village. Some bring vegetables to elderly women who provide care to multiple grandchildren while their mothers are working; some come to purchase duck eggs or deliver a bit of news, enjoy some fruit, and hear me speak Khmer—a source of great pleasure and amusement for many of the older women in the community. Prahm, one of the original creators of the community, explained the importance of the village as a cultural space. He said," Here [Veluvanna Village] I can close my eyes at night and feel safe." (p384) [Lewis 2009; U]
The participants revealed that the activities that they preferred, such as Tai Chi and square dancing, were mostly clustered in the city parks on the Gold Coast. The participants' residences were scattered across the Gold Coast, which meant that few of the	As indicated in Figure 3, one-fifth of the participants performed Tai Chi within 7 days before the interview. Two participants, Dong and Han, a couple, played traditional Chinese instruments in the park near their homes… Figure 5 shows the travel maps of the 18 participants within a 1,000-m zone

participants were able to visit their preferred parks. [Gao 2020; C]	highlighted on each map. It showed that the participants' behavior in visiting parks and their travel tracks depend on the distribution of parks within a 1,000-m zone (see Figures 5A–C,E,F). They could walk to the parks near their home, which indicated that the accessibility of parks could partly meet the participants' needs. However, they had lower accessibility to their preferred park." (p. 6) [Gao 2020; C]
Social support was also linked to participant assessments of the physical environment. [Hawkins 2022; U]	Specifically, participants would go walking with their friends on sidewalks and through parks. In this way participants had physical and social benefits, even as they experienced age-related decline (photo one shows participant sitting on her walker after walking to the park.): "Now our difficulties are all connected with our health, we are already attached to out walkers. When I arrived 10 years ago, I didn't have a walker, and I walked by foot...45 minutes by foot, and we had the company of three people from our home (building) and we would walk (by foot)...(Now) we have fun with the walker, walking somewhere around the house (building)." Participant 2 (75 years old) Photo eight. Taken by participant 2 of participant 4, who walked every day." (p. 114) [Hawkins 2022; U]

Lack of accessibility and safety in transportation caused by discrimination based on language, race, and disability restricted access to valued amenities and social spaces within and beyond the neighborhood which exacerbated social isolation. Transportation is most critical to prevent isolation when culturally and linguistically familiar spaces are located outside neighbourhood boundaries.

2.1 Discriminatory Experience in using Public Transport:

Author Statement	Illustration
Many participants reported exclusion from basic services including amenities, healthcare, housing, and mobility, etc. Going to church or attending religious activities proved to be important components of immigrants' daily lives, especially for older people. However, limited physical mobility prevents them from attending religious or cultural activities. (p.7) [Xu 2023; U]	P6 (Female, 77) mentioned that she could not go to the traditional Chinese dance club because of her back pain or discomfort. Another participant mentioned that she and her friends could not go to church due to impaired physical ability. I don't go to any [religious meetings]. I am old. [The church] wants me to refer my friends, but they won't go. There is no transportation, they (friends) said their feet are not good anymore and couldn't walk. There is no point (to go or invite people to church). We are not able to do that. Where could they go? . . . You are too old to do such things. (P9, Female, 90) [Xu 2023; U]
Many participants who wanted to travel independently shared the difficulties they experienced with public transport, which was described as deeply unreliable with buses rarely run to schedule. Some participants felt they could not rely on bus drivers to help them get on and off the bus, which meant they often stayed home rather than risk embarrassment. [Morgan 2021; U]	A narrative example from the Chinese group, told in the course of a heated discussion about the wider prejudice older migrants experience, illustrates: y response: Old men like us came to this place, it seemed we were taking advantage of the government. This prejudice is added to our group. Society cannot have this kind of prejudice. After our children graduated from college, we agreed to come to New Zealand. We spent a lot of time and energy looking after them until they grew up. We applied for the visa and the government was willing to accept our applications. This is fair. People here cannot always think we are profit at another's expense. **Speaking of loneliness, there may be some because we are not familiar with**

	**the social systems, but the mainstream's view about us makes us feel even lonely. For example, they just talked about the bus. If it is a local Kiwi waiting in that place, the bus would stop. If they see a Chinese person waiting in that place, the driver would not stop the bus. Sometimes when we got on the bus, and ring the bell, they still keep driving and stop at the next bus stop. It took us a long time to walk a long way back.**<br>Z respond also: This happens a lot, and sometimes we are making jokes with each other, saying 'can you imagine how good their driving skill is?' Obviously they see us standing here, but keep driving to another place to stop. We are 70 or 80 years old, but the bus drove past us and stopped ahead where it was more than ten metres away. I was joking about it: it's testing my ability to walk. Is my driving skill so bad? (Nodding from all members of the group) (Chinese men, CG) [Morgan 2021; U]
Though senior-specific programs and activities were available through the local seniors' centre, some older adults were less mobile than others, making these difficult to access [Fang 2016; U]	one suggested that some older people would benefit from various 'in-house' activities. The main challenge was acquiring human capacity to organize and implement programs that "involve our hands and minds." Coordination and implementation of age-friendly activities required time, space, and place organization. One solution generated by participants was to raise funds to hire a program coordinator to organize activities and establish a tenant board. [Fang 2016; U]
Participants said that stepstool and assistance with walkers may facilitate the use of transportation services. [Dabelko-Schoeny 2021; U]	Number one, they have to have some kind of a stepstool to get in. If it's too high,they can't ... Then all the walkers,they were flying all over. There was a very small space for a person to just sit, and they just burst into tears because they don't ask what people need. (Russian)." (p. 6) [Dabelko-Schoeny 2021; U]
Some participants did not have access to a bus stop in their neighborhoods, did not have bus shelters or benches at their stops, or the bus stops close to their home were not covered. Therefore, in extreme weather conditions,	One English speaking participant commented "On the bus, if you go to the grocery store, technically—you are only allowed three bags". (p. 6) [Dabelko-Schoeny 2021; C]

they did not want to use the buses.In addition to bus stops and shelters, carrying and storing groceries on the bus also appeared to be challenging to older adults, particularly those with disabilities. [Dabelko-Schoeny 2021; C]	
Similarly, older adults from Somalia also identified the lack of alternative transportation options in the community [Dabelko-Schoeny 2021; U]	"One participant who previously lived in Europe said "the majority of us lived in city with excellent public transportation, subways, trolleys, etc. [blinded for review] is limited to bus" (Russian B)...<br>"... in Somalia there were buses ... There were taxis. See there were what they call tuk-tuk—with three legs." (p. 6) [Dabelko-Schoeny 2021; U]
Participants identified personalized services and coordination of services to be important. [Dabelko-Schoeny 2021; U]	One participant talked about a ride share company with trained staff and accommodations available for some older adults. They're very professional. They're on time. They don't mind helping you with packages, where others sit in the car. They don't mind givin' you a footstool,if you're handicap, to make your entry a lot easier.Let this companytrain the rest. Put them through some type of service periodically ... Don't let a [sic] accident happen before you correct something when we're sitting here tellin' you, as seniors who use the service, that we need. There's a company called Blessed Choice that should be training the rest of them. (Russian A). (p. 7) [Dabelko-Schoeny 2021; U]
Lack of subsidized housing and transportation for seniors with a disability [Brotman 2017; U]	"Birds can fly everywhere. They are free! That's what I want. But I have this. I have this disability. I can't go anywhere." After the accident that left Divine permanently disabled, she realized that she needed to reduce her housing costs and approached BC Housing for a subsidized suite. She was told that with her health challenges, an appropriately modified suite would be harder to find. When she finally got an offer the suites were located in municipalities that were 1-1.5 hours' drive away from her current

	location. Divine told us through an interpreter how her family doctor objected: "No, because she don't have any family. She don't drive. And she always come here every day. It's hard for her to go so far away." Divine could not afford to sacrifice the invaluable support of her friends who lived nearby and the family doctor who had been a critical advocate for Divine's care. Instead, she found a subsidized suite through a religious organization, but the building is not adapted for persons with disabilities. Divine's suite is on the second floor, the laundry is in the basement, and there is no elevator: "I fall down, but I stand up because I want to live, to get back to my life. Then the following week, I want to (small chuckle) go to the laundry because the laundry is down... Uh, it's 13 steps. I go there because I don't want somebody to get my clothes.... I fall down again from 13 to down. So another! This one I have some cuts here (gestures to arms), my glasses and this one here (gestures to face), the big cut....So I crawl to the stairs because there is a hall, we can hold the stairs... the railings." Divine gets impatient waiting for the caregiver to do everything because she always comes late. She worries about how long she can remain in this place. The rent has already increased from $365-$408 per month, but her disability pension did not. She also struggles to pay for transportation. HandyDart is not covered by the Compass Card she needs to purchase to ride public transportation. Divine does not believe that she could afford Assisted Living, and she is unsure about her eligibility because (ironically) "they asked me to get a doctor's certificate [to prove] that I can still take care of myself." (p. 64) [Brotman 2017; U]
The interviewees reported that the accessibility of public transport varied throughout their neighbourhoods, as some stops were lower than the vehicle entrances. [Jagroep 2023; U]	Especially for those who used walkers or wheelchairs, these stops were not accessible: Sometimes stops are very low, which makes it difficult to get on or off the tram. They have made some stops a bit higher now, but not all

	of them. And you do not always know where the stops are too low. (Participant 2) (p. 9) [Jagroep 2023; U]
Access to public transport was a salient issue in most interviews. (p.838) [Lorinc 2022; U]	Aine (82, Irish), who lived in rural Yorkshire, mentioned that bus routes were reduced to “one an hour. It used to be one every half hour.” Having her doctor’s surgery in the next village and only basic shops close to her house, Aine depended on public transport. (p.838) [Lorinc 2022; U]
Although some participants made enormous efforts to get out every day to socialise with friends and attend community associations, for others material obstacles hampered their efforts to remain mobile. Wider changes to infrastructure can have major implications for navigating place and this was especially the case for our participants in more suburban or rural areas.(p.06) [Ryan 2021; U]	Mandek and Gabriela, a Polish couple living in Yorkshire, explained that recent changes in bus routes had significantly impacted Mandek's (78) ability to get into town. By contrast, although London is well served by public transport, it was not always accessible to older users, especially those with mobility limiting conditions. Jadwiga (74, Polish, London) had difficulty walking and was unable to use the nearby tube station because it had no lift access.(p.06) [Ryan 2021; U]
Taking public transportation caused anxiety not only because of risking bus/metro routes or stops but also because of racial discrimination. Mrs. Yip was a business owner in Hong Kong but spoke little English or French. After immigration in 1968, she and her family ran a Chinese restaurant.p.337<br>… Living in multiethnic neighbourhoods might indicate success in objective integration for Chinese immigrants but monolingual seniors found these areas difficult. [Hsu 2014; U]	One of the participants Mrs. Yip said, “I used to take the bus to work.<br>However, my long hair smelt after a day in the kitchen. People on the bus wouldn’t let me sit nearby. They told me off or even asked me to get off the bus.” Decades after, Mrs. Yip refused to live with her daughter in an American suburb. “My daughter wants me to go to the U.S., but I don’t want to. The house there is huge and is surrounded by gardens, but it’s very inconvenient for me.” (Interview, 13 Dec 2008). P.337<br>Mrs. Tu retreated to the private sphere of the household because she felt scared and ignorant even facing road signs, the most mundane elements of public space. Mrs.Yip preferred to drive because the bus was a public space where bodily odor and practices from the Chinese kitchen were the target of face-to-face racism. Physical<br>and cultural boundaries invariably became racial edges for discrimination exacerbated

	by oral non-communication. To quote Mrs. Hui, who immigrated in 1990 at theage of 60 years, to navigate a new place without the local language(s) felt like “being deaf and being mute” (Interview, 16 Nov 2008). [Hsu 2014; U]

2.2 Having a car improves the quality of life:

Author Statement	Illustration
Access to a Car Is a Necessity for Most. Related to the theme above, having easy access to a car was a perceived to be a necessity. Twenty-one people either drove themselves or had access to a car when, for example, a child or grandchild would drive he person where they needed to go. (p.07) [Yen 2012; U]	A woman living in Oakland (72, Caucasian) said, “Well, we wouldn’t be able to stay here without being able to drive. I suppose we could use taxis, but that would be the only alternative.” A 69-year-old, African American man in Oakland said, “I would say that there are no stores. And that’s one of the major difficulties of living here is that if you don’t have an automobile you’re up the creek.” (p.07) [Yen 2012; U]
A strong theme running through our data was the ongoing efforts required to remain active. Moreover, the ability to do so was often shaped by particular material characteristics of places in which people lived and the necessary economic resources to navigate those places. With declining health and mobility, participants described the efforts required to negotiate places that were once easy and familiar. Several participants mentioned the importance of driving and being able to afford a car. (p.06) [Ryan 2021; U]	Samantha (65), in Yorkshire, originally from Jamaica, had a range of complex health issues including arthritis and asthma which impacted her mobility. Relying on her car, Samantha dreaded to think about how she would manage without it: ‘It's my independence’. However, several older participants were no longer able to drive. Mona, an 82-year-old Irish woman in Yorkshire, spoke about problems with her knees: ‘I do have a walking stick, because I'm not going to risk falling’. Although unable to drive anymore, Mona was determined to get around: ‘I'm a great bus woman’. She travelled by bus into the city-centre at least twice a week to meet friends for coffee or just window-shop. [Ryan 2021; U]
Out-of-home mobility is largely affected by the fact that only two women currently had a	Ka-Lee would like more activities at the community center closest to her home because she does not drive. Her husband drives but,

driver's license, and many reported never having driven. (p.644) [Tong 2020; U]	she explained, "he can't drive me everywhere ... it's too much for the old man, over 80 years. If [activities] were here then I would have the freedom to come anytime." (p.644) [Tong 2020; U]
Limitations, such as an inability to drive, has caused a shift toward elders being homebound, engaging in temple participation solely on weekends, and infrequent trips to visit family, friends, or markets because of the need to rely on younger family members who often are working….As her narrative shows, it is more difficult for elders to achieve the level of freedom they expect. Elders, such as the woman described above, limit their interactions with non-Khmers because of language difficulties and what they perceive as suspicious looks from neighbors. The critical aspect of neighborliness and belonging (Casarett, 1991) is missing.<br>(p387) [Lewis 2009; U]	One older woman, living in a small house with her daughter while caring for her young grandson, described how she had lost her freedom when she arrived in the United States. No longer able to walk to the temple, to markets, or to visit other elderly women, she feels trapped. She explained, "I am afraid to walk around my neighborhood. I don't know the people; they only speak English. They watch me when I walk so I am afraid." She stays inside her small frame home and only ventures into her fenced backyard an hour or so each day so her grandson can play. She is as suspicious of her non-Khmer neighbors as they are of her. She explained that, in Cambodia, she remembers her mother walking to visit other women, walking to the market, and feeling free to go into the forest to gather fruits. She is happy that she is in the United States but misses the ability to move about freely. She is acutely aware of a disconnection between her memories of place in Cambodia as connections across relationships and with the land and present-day discomfort with unfamiliar terrain and customs. "Here I have my grandson," she continued, "I make my daughter and her family happy; I pray to Buddha. I am peaceful inside [the house] with my small family. Next time [in her next life] maybe I can be free, too."<br>(p387) [Lewis 2009; U]
However, contributions to the household and provision of childcare was primarily limited to the confines of the home due to limited access to the community. [Wijekoon 2018; U]	For instance, Sujatha, who does not drive and has a limited understanding of her geographical surroundings, spoke of her inability to assist her grandchild in the event of a medical emergency. Without someone else present, it is difficult to care for these children in an emergency. If suddenly the

	child develops an illness, it would be problematic. Someone has to be present at home. She [granddaughter] was vomiting. Thankfully the daughter-in-law was here. It would be scary if I had to urgently take her to the doctor. – Sujatha (p. 143) [Wijekoon 2018; U]
Furthermore, in an automobile dependent culture, these suburbanite non-driving participants felt confined and excluded from broader society. (p. 164) [Wijekoon 2018; U]	...now when you enter your home, all the doors and windows are closed due to the cold. Once you enter the home, you are isolated. You cannot see your surroundings. If you look at the road, you rarely see any people walking. Now in Sri Lanka if I happened to step out from the yard, go out to the road and walk along the road, I see people. There are people everywhere, crowds everywhere. [I see] people talking, making jokes, screaming and shouting and engaging in various types of discussions. I would see familiar people, [they] will ask 'where are you going?', 'how are you doing?'. Worries and concerns within my mind would vanish. So, I did not feel lonely or isolated there. That is what I felt after coming here [to Canada] after spending 75 years [in Sri Lanka]. I couldn't bear it in the least. I initially wondered whether I could tolerate it. – Bandula [Wijekoon 2018; U]
Older immigrant participants shared that they relied on friends and neighbors for transportation, particularly when family members were not available, [Dabelko-Schoeny 2021; C]	"far friends are not comparable to close neighbors" according to a Chinese-speaking older adult." (p. 7) [Dabelko-Schoeny 2021; C]
Regardless of language preference, participants across focus groups unanimously agreed on the importance of having an automobile and being able to drive. The category of personal vehicle as a transportation facilitator was mentioned 22 times across all groups, a subtheme with the highest frequency. Considering the importance of automobiles, Somali older adults emphasized educating the younger	They say we focus as families for our children to learn how to drive and get a car. Because in America your legs is your car. That's what we put emphasis. Most of our families, we say most, we have good private cars. For our children maybe, for others most of the time. Yeah. If you find sometimes that one person living alone not having a car—yeah? - for him to get somebody's health aid if he doesn't have any car and he's alone ... Most of us, majority of us, of Somali families have car.

generations to drive as a strategy for family mobility. [Dabelko-Schoeny 2021; U]	We focus on teaching our children or those 16-years-old, 17-years-old, to learn how to drive, so that make our life easier and his life easy (Somali). (p. 8) [Dabelko-Schoeny 2021; U]
Participants expressed concerns with driving due to aging-related vision loss and difficulty navigating technology. [Dabelko-Schoeny 2021; U]	It is very hard for me to drive out of the state because I'm old. I have eye problem. Also, I can't read those number. I don't know how to use GPS, so it is very hard for me to drive out of the state or out of town. (Bhutanese). (p. 8) [Dabelko-Schoeny 2021; U]
Russian-speaking participants further explained perceived consequences of not being able to drive: [Dabelko-Schoeny 2021; U]	**"When we will have no transportation, we will buy a place in cemetery ... Because we will have no life. Without my car, I will not be able to walk to any stores" (Russian B) (p. 8) [Dabelko-Schoeny 2021; U]**
Similarly, participants also associated not having a car with the possibility of relocation. [Dabelko-Schoeny 2021; U]	At 80 years old, I am lucky to still be driving. Otherwise, I would have to move from my home" (Russian A). (p. 8) [Dabelko-Schoeny 2021; U]
Oana's health also did not allow her to visit Romania. She missed her friends from Romania the most and explained that the public transportation system in her current neighbourhood was insufficient in order for her to make and visit friends in Canada. Not having a driver's license, nor a car, made it difficult for her to get around the city independently. [Schuster 2019; U]	"Here, it is very difficult to have friends, because it is impossible to reach them. I don't have a driver's license and I depend on my daughter to drive me." "It takes me more than an hour to get to the first bus station. And there, if I go, I have to change the bus. [...] If I go somewhere, it would take me the whole day." (p. 68) [Schuster 2019; U]
An issue that all participants encountered was the location of their home and insufficient public transportation. [Schuster 2019; U]	Their houses were each embedded in large neighbourhoods that were distant from the city centres, malls, libraries, and other facilities. Only Elena had a driver's license, but she did not own a car, so none of the participants were able to drive to their desired destinations by themselves. Furthermore, the participants did not live close to public transport hubs which required them to change bus lines several times. Each of them reported that taking a trip somewhere by bus was time consuming, and therefore, they avoided it. In addition, Samantha, Elena, Gizella, and Oana

	said that they did not want to bother their families by asking them to drive them around, and therefore, came to terms with staying in the house. "I don't have a car, I don't drive. So, I must take the bus [...]. They [children] cannot drive me to all these activities. That's why I am here [at home]. [...] In my opinion, the bus has not very good service. So, it's difficult for me, but what can I do?!" (Samantha) "If you do something out of the house, you are going to stay out of the house the whole day. And you need a car. You need means of transportation. That in my age is difficult at some point. [...] But I don't like to take their car because they need their car, and I don't like to bother them." (Elena) "I don't own a car. [...] We don't use it [bus]. [...] I didn't have the need to [leave the house by myself]." (Gizella) "I don't have a driver's license, and I depend on my daughter. And when she comes home from work at 6 PM, how can I tell her: 'Bring me to my friend's house!' [...] I just think after she spent so many hours at work, she returns home tired, and then I would also give her my problems on addition." (Oana) (p. 81) [Schuster 2019; U]

2.3 Lack of access to public transportation

Author Statement	Illustration
Among many public facilities, the public transportation service was highlighted among many immigrants living in Brooklyn and Queens as a key to their aging place. Older adults can either visit the local store and facilities within distance or travel to other Chinatowns through public transportation. (p.384) [Chen 2022; U]	Older adults should live in Brooklyn as we can either walk or take public transportation to get to the place we want to visit. (P18, Female, 80). (p.384) [Chen 2022; U]
Participants shared that maintaining a car and using taxi services were "expensive."	One Bhutanese older adult said, We get very little money. With that, we have to pay rent.

Although the public bus was less expensive than other options, older adults noted a lack of financial resources to spend on bus fare. Having a bus pass was also identified as an important factor in using bus service. [Dabelko-Schoeny 2021; U]	We have to buy food or clothing. We don't have enough money to spend on the bus. If we get a bus pass, that would be a huge help (p. 5) [Dabelko-Schoeny 2021; U]
Lack of subsidized housing and transportation for seniors with a disability [Brotman 2017; U]	"Birds can fly everywhere. They are free! That's what I want. But I have this. I have this disability. I can't go anywhere." After the accident that left Divine permanently disabled, she realized that she needed to reduce her housing costs and approached BC Housing for a subsidized suite. She was told that with her health challenges, an appropriately modified suite would be harder to find. When she finally got an offer the suites were located in municipalities that were 1-1.5 hours' drive away from her current location. Divine told us through an interpreter how her family doctor objected: "No, because she don't have any family. She don't drive. And she always come here every day. It's hard for her to go so far away." Divine could not afford to sacrifice the invaluable support of her friends who lived nearby and the family doctor who had been a critical advocate for Divine's care. Instead, she found a subsidized suite through a religious organization, but the building is not adapted for persons with disabilities. Divine's suite is on the second floor, the laundry is in the basement, and there is no elevator: "I fall down, but I stand up because I want to live, to get back to my life. Then the following week, I want to (small chuckle) go to the laundry because the laundry is down... Uh, it's 13 steps. I go there because I don't want somebody to get my clothes.... I fall down again from 13 to down. So another! This one I have some cuts here (gestures to arms), my glasses and this one here (gestures to face), the big cut....So I crawl to the stairs because there is a hall, we can hold the stairs... the railings." Divine gets impatient waiting for the caregiver to do everything because she always comes late.

	She worries about how long she can remain in this place. The rent has already increased from $365-$408 per month, but her disability pension did not. She also struggles to pay for transportation. HandyDart is not covered by the Compass Card she needs to purchase to ride public transportation. Divine does not believe that she could afford Assisted Living, and she is unsure about her eligibility because (ironically) “they asked me to get a doctor’s certificate [to prove] that I can still take care of myself.” (p. 64) [Brotman 2017; U]
Given the ability of environmental factors, such as a lack of transportation services, to compound their experience of exclusion (Blanco & Subirats, 2008), Saskatoon’s Chinese-Canadian older adults rely on alternatives to the linguistically challenging process of taking public transit. Those relying on bus services for daily mobility expressed concern over their knowledge and access to specialized age-based services. [Herman 2021; U]	If your English is not very good … you cannot get information and that knowledge of how to use those [age-based transit] facilities. He mentioned an example, like someone in a wheelchair…you cannot walk around easily and you cannot drive. So he mentioned someone in his building who just calls some place, and there will be a bus that will take them…but for the [Chinese-Canadian] elderly people in his building, they do not know how to do that part. (Stated by interpreter, Participant 7: Male, 70–84 years old). [Herman 2021; U]
Language has become a transparent fence of the “place”. All the participants have highlighted the language barrier as a significant concern. Some participants felt restrained with limited English proficiency and could not travel on the subway or visit the general hospital independently. The insufficient language skill has limited their possibility of exploring the outside community and increased reliance on the enclaves. (p.383) [Chen 2022; U]	I think it is all a matter of language. I cannot leave Flushing with poor English and don’t know how to take the subway. (P9, Male, 84) (p.383) [Chen 2022; U]
Lower language proficiency was identified as a major barrier of public transportation use among older immigrants and refugees. [Dabelko-Schoeny 2021; U]	Language is the biggest issue to use public transportation because we cannot communicate with the bus drivers and we don’t know which bus goes where. We don’t know any bus routes. That is also an issue. (Bhutanese). (p. 7) [Dabelko-Schoeny 2021; U]

Transportation and service materials translated into different languages might be helpful to diverse older adults. [Dabelko-Schoeny 2021; U]	What they're saying is that yes, definitely,if it was in their language, it would be easier, but even if it's in their language, and it's a very complicated letter, and it's a very complicated financial explanation, still, they need to have help. If something is more simplified and translated, great. (Russian B). (p. 7) [Dabelko-Schoeny 2021; U]
Having transportation information in their own language was not only associated with older adults' ability to navigate transportation systems, but also with their comfort of getting around. [Dabelko-Schoeny 2021; U]	We were laughing and we were very happy because it is Nepal.We know the language and there is no any issues knowing about the bus systems. We used to walk. We used to communicate easily and we used to know about transportation and everything. That was easy ... Our grandparents were born in Nepal. When we entered Nepal, we used to feel like Nepal was our home because of the language, culture, and everything (p. 7) [Dabelko-Schoeny 2021; U]
Outside of the temple, participants' socialization with ethnic peers was limited. Friendships forged at ethnic spaces were maintained primarily via telephone due to geographical dispersion and lack of independent and accessible transportation. Apart from the temple, participants infrequently met their ethnic peers at family dinners, private almsgiving events, and cultural shows. Although infrequent, these meetings were reminiscent of their lives in Sri Lanka. [Wijekoon 2018; C]	Now when we go to the homes of the Sri Lankan families that live here, we all get together and sing Sinhala songs. I enjoy that very much. I am overcome with the feeling that I am back in Sri Lanka when I sing those songs and speak to them [in Sinhala]. There we are all the same. There we act in our [Sinhala] way. – Apsara" (p. 155) [Wijekoon 2018; C]
In terms of medical transportation, unpredictable pick up times hindered older adults from getting to doctors' appointments on time. Participants also experienced long wait times for pick-ups and drop offs. (p. 6) [Dabelko-Schoeny 2021; U]	Many times people are waiting to go to a doctor.They have a certain appointment, and they may have to cancel their appointment if they don't get there on time. They come and pick them up an hour, 2 h later.Then they have to, of course,go there, park, and they have to get in and so forth. That is a major problem (Russian B) [Dabelko-Schoeny 2021; U]
The interviewees perceived public transport as a key resource that helped them to remain	They were satisfied with the public transport in their neighbourhoods, as it was reliable and

independent and participate regularly in community life. [Jagroep 2023; C]	easy to use, with stops within walking distance from their homes and various options (e.g. tram, bus, metro). (p. 9) [Jagroep 2023; C]
Various travel options for older adults contributed to the age-friendliness of a neigh bourhood, as older adults could decide for themselves what was feasible for them. [Jagroep 2023; U]	You can take tram 1, 9, 15. And on the other side there are other options. You can also take the bus. (Participant 14) (p. 9) [Jagroep 2023; U]
Participants living further from city centers noted a lack of shops and other services in their local neighborhoods (p.838) [Lorinc 2022; U]	There's just one corner shop ... just one, and they turned ... part of it is a post office, because ... most of the big post offices are closing down, and they're putting them in the shop ... So, if you want anything that's more, you have to come out and go to the supermarket. (Millicent) (p.838) [Lorinc 2022; U]
A supportive social environment consists of the psychosocial domain ("knowing people," having a place to volunteer and connect), the environmental domain ("the bus is near," "temple is near"), and the cultural (a culturally familiar place of worship, where her first language, Punjabi, is spoken). (p.643)<br><br>They get out the door and engage with their local community because they have access to activities, shops, services, and social or religious gatherings that are culturally familiar.(p.644) [Tong 2020; U]	These few blocks, these are my village. Because I know those people. [The] bus is near. And my temple is near. [When] I'm not feeling good I go there. And on Sunday I go and volunteer there . ... When we bought this house, we thought the gurdwara (temple) should be near—every weekend we should go. (p.643) [Tong 2020; U]
Lefebvre (1991) has suggested that such productions of – potentially new – forms of social space can be seen as an integral part of group identity formation, which he considers as a fundamental right of all inhabitants of the city. Next to the use of specialised shops and communal spaces, access to the local mosque was also considered to be of major importance by many. (p.22) [Buffel 2011; U]	A Pakistani respondent in Manchester reported how he had contributed to the construction of such "an own place" for the community where people could meet each other:<br>Twenty years back we hired the halls and then we prayed there… I said why are we hiring this hall…? Why don't we make our own place? So… you gave according to your pocket, I gave according to my pocket. We were all people who were praying there; everybody contributed something to that kitty you know… We bought that place where this mosque is now. [Buffel 2011; U]

Sixty percent of participants who responded were inconsistent with calculated accessibility scores. These conflicting results provided insight into the problems with word of-mouth awareness rather active marketing to inform older immigrants of what is available to them. [Dorkenoo 2021; U]	One Spanish-speaking participant (SF2-P1) with a “high” calculated score in their census tract stated, “We only learn about these activities late. Perhaps we learn about specific programs a week later after these have taken place. So that’s a gap in the service that we can see.” On the other hand, a Spanish-speaking participant mentioned the use of the senior centre Nueva Era, despite a “low” calculated score. This again highlights the role of attractiveness in a service that reduces the friction of distance that an older immigrant may experience. This was confirmed through mentions of the ACCT by Arabic-speaking participants who resided in census tracts with “low” scores. Consistent with their calculated access, a participant (AF3-P5) stated, “As far as I know, they are not services in my area that support seniors in Arabic. However, I know that the Arabic Community Centre in Toronto... [provides] help for seniors and [accommodates] their needs.” (p. 49) [Dorkenoo 2021; U]
The study interviewees stated that they appreciated having shops that carried traditional Surinamese herbs and vegetables in their neighbourhoods, as they had previously had to travel long distances to such shops: (p. 8)<br>… They mentioned that the availability of a Surinamese toko in the neighbourhood contributed to their feelings of social inclusion and belonging. [Jagroep 2023; C]	It's nice to have a Surinamese toko nearby, sometimes you want to make a Surinamese dish. (Participant 6) (p. 9) [Jagroep 2023; C]
Transportation was the largest and most pressing concern that emerged during group interviews, without any questions or talking points made by moderators. As previously mentioned, transportation costs to and from social support services such as community centres and social activities for older adults were unaffordable for older immigrants.[Dorkenoo 2021; U]	Comparisons were made between Toronto and other municipalities, as well as participants’ native countries:<br>“And the transportation in Mississauga is $1 for seniors.” – SF5-P3<br>“In China, as long as you are 60-year-old, or above, the transport is all free.” – MF1-P2<br>“Especially in Toronto, the public transportation fare is so expensive, more expensive than any other [major] Canadian city. For example, in Vancouver, although the

	living costs are higher there, their public transportation fare is cheaper. This makes mobility for elders inconvenient… More people will actually want to take public transportation if it's cheaper." – MF2-P7 (pg 31-32) [Dorkenoo 2021; U]
The underlying cost for participating in social programs or using social support services is transportation. In many cases, older adults do not receive any subsidy or reimbursement for using public transportation to reach services [Dorkenoo 2021; U]	"That's my concern. I don't go to all those places because I have to pay $2 and something. And to come back, the same. So that's a bit more than $4. I go to three activities during the week - and that's more than $12, $13. I want to go every day because I am very active but I don't have enough money to pay for that. If at least transportation was $2 in total… but it's $4. So that's what hinders me. It makes me stay at home…That's our main constraint. So, if we take the car… there's the gas. And parking. If from here, they take us to the theatre or somewhere else, you've got to pay again." – SF5-P3 (p. 30) [Dorkenoo 2021; U]

2.4 Valuing co-ethnocultural spaces within neighbourhoods

Author Statement	Illustration
Other common themes in our data included "Staying mobile," "maintaining independence," and "impact of mobility on well-being." (p.837) [Lorinc 2022; U]	Despite her reduced mobility, Agnieszka made an effort to go out every day, to different activities organized by Polish NGOs, exercise classes for older people, church, to meet friends and family, and also for walks in the neighbor-hood and the nearby parks. (p.837) [Lorinc 2022; U]
People Try to Stay Busy<br>Many of the participants were very active with social activities, work, volunteering, classes, and leisure travel. Several people talked about being "on the go," wanting to get out every day. In some cases, people intentionally went	"Well, I don't have family, I don't have anyone.I am not going to sit around. I am involved in many things with the church and other things I do during the day. I don't stay here. "[Translated from Spanish](P.06)<br>"My husband is just the opposite, he likes people. He thinks I should be more active than

out to keep mentally and physically active.(P.06)<br>Activity levels seemed to be a result of personality (being more or less outgoing), physical functioning (ability to get out), and the desire to maintain a similar level of activity as when the person was younger (possibly to retain a younger outlook).(P.6-7) [Yen 2012; U]	I am, but I'm not. I remember that in my family, my mother was the extrovert. She was the one who knew everybody and their business. But I just don't. I'd rather stay home and read".(P.07) [Yen 2012; U]
People Able to Leave Their Homes Do Many Activities Outside<br>Their Immediate Residential Neighborhoods. Half of the participants went out of their neighborhoods at least once a week for a variety of reasons. In addition to the activities listed above, weekly activities included going to the movies, participating in hobby or social groups (e.g., bowling league and hiking group), food shopping, caregiving for a friend or relative, window shopping at the mall, or visiting family. Other regularly occurring (monthly or quarterly) events included going to doctor's appointments and picking up prescriptions.(P.07) [Yen 2012; U]	An 82-year-old man went to the movies twice a week, generally travelling on foot and by bus to go to his favorite theatre about three miles away from his home. A 69-year-old woman drove about seven miles from her home in Oakland to Oakland's Chinatown, two to three times per week, to volunteer activities and buy food.(p.07) [Yen 2012; U]
An insufficient supply of resources can also contribute to older Chinese immigrants' social exclusion from community services (p.7-8) [Xu 2023; U]	There are no community activities during holidays. This is the worst. For example, on Saturdays or holidays, these places are all closed. The older people will be like . . . they don't know where to go, because their children are mostly in other states. So, we elders can only stay at home during holidays. (P20, Male, 68) [Xu 2023; U]
Participants described both the mental and physical benefits, an overall sense of well-being that comes from getting outside and being active. Participants listed a range of benefits associated with their neighborhood walks: keeping the heart strong, keeping the brain fit, reducing stress, rais- ing their spirits, maintaining their weight, and preventing disease. (p.643) [Tong 2020; U]	Oh, I'm going for the fresh air and I want to meet the peoples and I want to make my feelings better ... I like the flowers and trees and weather. And I want to happy myself, you know ... It's good. And it's good because, you walk and your heart will be strong and your brain. Then you get thinking about that ... the brain is fresh all the time when you go out. If you sit there at home, you are sleeping and you will be lazy. (p.643)<br>[Tong 2020; U]

participants described the psycho- social benefits of getting out to see others on their walks, be it passersby, neighbors, or friends with whom they walk. Psychosocial benefits included the following: feeling more positive and uplifted when walking with friends; feeling "happy" to observe nature (trees, gardens, wildlife, etc.); feeling connected to strangers who simply say "hello" as they walk by; and feeling connected to familiar faces in the neighborhood. (p.643) [Tong 2020; U]	"I want to meet the peoples," and she went on to explain that she enjoys chatting with the 90-year-old Chinese widow who lives across the street and takes pleasure in hearing the laughter of children playing at the nearby playground. (p.643)<br><br>I feel stressed sometimes. But when I go out meet people, I forget about it. Talking to someone about here or India relaxes the mind ... So walking alone, I don't want to walk too long, I'm quiet. With friend it's—we are chat-ting and we—I don't even realize we've gone so far. That's how it happened, there's the difference. (p.643) [Tong 2020; U]
Participants repeatedly cited getting out the door for culturally familiar shops (e.g., the local Punjab—South Asian—market and mini Chinatown), restaurants, religious and social gatherings. (p.644) [Tong 2020; U]	They make the tea and everybody do something, any song, any stories, what happen there. They tell stories or sometimes sing songs. Functions for Mother's Day. We celebrate the Diwali there and Vaisakhi. [The interpreter added, these are festivals. Indian festivals that they celebrate there.] (p.644) [Tong 2020; U]
An added benefit of these culturally familiar activities, shops, and services is that they are typically linguistically accessible. Participants were getting out of their homes, moving their bodies, and engaging with a local environment that is linguistically accessible and culturally familiar. (p.644) [Tong 2020; U]	Ka-Lee, who has voluntarily run a Luk Tung Kuen class, in Cantonese, for more than 20 years, stated:<br>According to my experience that's [have an active life without speaking English] doable. That's why I also encourage people, I say, look at me, I don't even speak English but I can still be active and do the volunteer work and you can speak different languages. You can also do that, too. And I'm also volunteering for Red Cross right now. (p.644) [Tong 2020; U]
According to older persons, to prevent social isolation and facilitate participation and engagement, it is important that social activities are: held in convenient locations, are frequent, available at different times and accessible for persons of various cultural backgrounds.	It was expressed that many older people living in the new development had lived alone and "don't have family here." One individual suggested that to promote social participation, the management could arrange for a "band from time

… Equally important is the accessibility and availability of age-friendly programs, activities and social gatherings. [Fang 2016; U]	to time" and "once in a while, have a little barbecue." The desire for more social activities was echoed by several older persons. Some felt that if "older people can get together, it might make them feel less lonely and increase their sense-of-place attachment." For example, "they could set up a weekly event to bring people together to either sing, dance or just chat." [Fang 2016; U]
Finding a place called home, based on the experiences of these Chinese seniors, appeared to have a lot to do with the weather, the natural and built environment, and the standard of living.<br>Similar to earlier studies weather is found to be an important factor in old age. Bad weather or winter seasons can be major confining factors for older adults.. This study also reveals that some seniors moved to the south for the warm weather to avoid cold winters like in Boston. In addition, seniors in this study echoed findings in earlier studies that emphasized positive emotions such as comfort, joy, and relaxation in their perceptions of making Atlanta their home. Clearly, the natural and built environment is directly linked to individual's emotional perception of the home. [Zhan 2017; U]	Beyond the warm weather, the natural built environment is another reason that adds to the Chinese seniors' sense of home. Ms. Qin described her sense of home in this way: "When I go out, I go inside the park, children's playing ground makes me very happy. I take a walk every day and enjoy the sunshine, which makes me feel good. Also, the library–when I was in better health, I used to borrow every kind of books to read. I also borrowed foreign DVD (meaning non-Chinese). So people in the library were very nice to me. There were Chinese books there too." [Zhan 2017; U]
Some older migrants also create 'places of belonging' by participating in residents committees, community centres and religious associations. In general, we found that older women's participation in such activities tended to be rather limited, especially among Somali people. However, some women had important roles as volunteers in self-help organisations and community centres.(p.102) [Buffel 2013; U]	An older Moroccan woman in Brussels, for example, said:<br>I go to the community centre every day. I help with cooking and I'm involved in organising activities so that we can do things together . . . it's important to mix with people from different cultural backgrounds. (-year-old Moroccan woman, years in the neighbourhood, Old-Molenbeek, Brussels)(p.102) [Buffel 2013; U]

Connected to discussions of the stability of the area and of co-ethnic social networks, religion featured in a number of ways in the narratives of the Indian respondents. Participants described how having a temple within easy reach and going to the temple enabled them to maintain connections with their 'community' and also that others in the The temple provided friendship networks that lasted over long periods of time. Indian participants also identified the importance of being able to celebrate religious events and festivals to their well-being. In fact, religion appeared to take a very central position in many of the Indian participants' lives, so living in an area where they could feel connected to their religion, where their religion was present and could be celebrated, seemed to be very important to their well-being.(p.554) [Becares 2013; U]	We are friends with everyone in the temple. Because everyone is a Satsangee [attendee of prayer meetings] and we know each other for a long time. We meet every weekend at the temple for Darshan [a form or religious worship]. So we have good relations with all of them. (589, Indian man in his early 60s). [Becares 2013; U]
The importance of religion was also present in many of the interviews with Caribbean respondents, where Black, typically Pentecostal, churches were discussed. However, the centrality of this to experiences of their locality and in shaping friendship networks was more variable in their accounts compared with the Indian participants. The geographical proximity of churches was not discussed, nor were churches discussed when participants were asked about their local area. Some of the Caribbean participants discussed the friends they had at church, others did not, even though religion was very central to their daily lives.(p.554) [Becares 2013; U]	R: Oh I've got a lot of friends because I go to church on Sundays so I've got a lot of friends, church people you know. I've got local friends as well you know, we get on quite well, we<br>phone each other and things like that you know. So I'm not bad, could be worse. It could.<br>I: How often do you kind of meet up with friends? Would you see them every day or a couple of times a week?<br><br>R: No I don't meet up with them really we more talk on the phone. And probably if we're going shopping and they are, something like that. But not very often.<br>I: And church ones obviously you see at...?<br>R: Oh yes.<br>I: How often do you go?<br>R: Well I go Sunday, I go Tuesday night class and then Friday night, Thursday night prayer meeting.<br>I: So you see them...<br>R: Oh yes I see them quite often. And they're quite good. If I'm sick and I take the phone up

	oh god you would see a host of them come in. (816, Caribbean woman in her late 60s/ early 70s). [Becares 2013; U]
While these discussions of stability, co-ethnic neighbours and religion point to some differences in the experience of their local areas that Caribbean and Indian participants had, the sharpest contrasts between their accounts emerged when they were asked directly about the area in which they lived. As might be implied by the accounts above, Indian participants discussed the positives of their residential areas and the amenities they have access to, particularly in relation to the presence of temples, community centres and the fact that there were other residents of the same ethnicity.(p.555) [Becares 2013; U]	There are a lot of our people around here. No one fights, everyone is nice and friendly ... everyone is nice to me. They always say hello to me... Yes it is all right. It is safe. There are many of our people here so it feels safe... The Gurudwara [Sikh temple] is only 10 minutes and the temple is 15mins. I can walk there ... There are buses near the street. It is only a 5 minutes walk. The service is good. I take a bus to go to the city. (958, Indian man in his late 60s/early 70s). [Becares 2013; U]
Older adults across various age, income, and socioeconomic status perceived that their place of congregation not only was a source of support but also provided them opportunities to be involved in their communities. Congregant memberships represented social connection with a larger group (i.e., social cohesion) that bolstered opportunities for civic engagement. As such, older Hispanic adult participants in our study who resided in low-income sections of the city believed that building a much-needed church would greatly help them develop a sense of community, provide resources based on this social network, enhance intergenerational relationships, and further motivate them to participate in various activities within the community (p.501-502). [Parekh 2018; U]	A 62-year-old participant reiterated:<br><br>We are trying to start a church here. The church is very important in this community and is the first place for the children.... so that the children can grow up in the church. ...in a Christian atmosphere and the elderly people of the church are helping us take care of children and teach and do different things. [A focus group participant] [Parekh 2018; U]
The participants interviewed for the study also commented on how they had worked to transform their current physical environment into a meaningful place, one where they feel at home and to which they felt attached to varying degrees. Such a sense of home was itself both created and reinforced through the	**'I wouldn't want to [move away from here] because I don't want to live far away from all the Turkish people. When I go out here I always meet family and friends on the street; and that gives me a sense of relief. I am already in a foreign country… if I would live somewhere far away from the**

proximity of members of their own cultural community, creating opportunities for common social bonds. (p.05) [Buffel 2017; U]	**Turkish community, it would feel as if I'm moving to a foreign country for the second time' (69-year-old woman).(p.05) [Buffel 2017; U]**
The Turkish community had become more visible with the building of mosques, the establishment of ethnic and religious associations, the celebration of religious holidays, and the opening of ethnic businesses. This community is often referred to as 'Little Turkey' as it has formed a self-sufficient enclave, where Turkish restaurants, halal butchers, teahouses, groceries, jewellers, and bookshops serve their predominantly Turkish clientele. Access to such 'third places' where 'imagined communities' can be sustained<br>was considered to be of major importance by many…(p.07) [Buffel 2017; U]	'Everything we need is close by: the mosque; the hospital; the pharmacy; Turkish markets; halal butchers… My children live nearby and I have Turkish neighbours. For me, a good neighbourhood is one with good neighbours and good streets where you feel safe… What else do we need? (64-year-old man)(p.07)<br>'We found peace here…I got to know my nextdoor neighbours well…people are warm, there is a good atmosphere and there are plenty of Turkish markets and shops… I can walk to the grocery round the corner to get fresh vegetables… And we also have a mosque nearby'<br>(63-year-old woman).<br>'I love this neighbourhood. It feels a bit like Turkey. It's like "back home". We have a mosque; there are many Turkish shops where we can buy cheap products; there are Turkish restaurants, and we know all the owners' (63-year-old man).<br>(p.07) [Buffel 2017; U]
Ethnographic observations demonstrated that Turkish older men tend to have more informal gatherings with friends outdoors than women, for example in the mosque or teahouses. During the interviews, there was a tendency among women as well as men to refer to these places as 'male spaces'. Teahouses in the neighbourhood were exclusively geared towards the need of Turkish male residents, offering opportunities to play games, gamble, watch Turkish football and socialise with other older men, who in most cases, originated from the same village of origin.(p.07) [Buffel 2017; U]	'I usually wake up quite early, I have breakfast, and then I go to see [his friends] in the Turkish teahouse around the corner… What do we do? We drink tea, just chat about general things… play tavla [backgammon]… And on Friday I go to the mosque. They [his friends] go as well.'(p.07) [Buffel 2017; U]

In contrast, the women in this study visited the mosque less frequently (in most cases only on important religious days), and often reported a lack of ‘female spaces’ or family-friendly places in the neighbourhood. [Buffel 2017; U]	‘There are no activities around here, especially for the women. What we need here is a social space where we can take our grandchildren and have a chat with other women: A place for the women to get together.’ (p.07)<br>Context: Islamic prescriptions, outlining that men and women should keep sufficient physical distance and should not meet in private, may further support ‘male territoriality’ in this neighbourhood, because women should not enter a space in which men are already present (Peleman, 2003: 159). The capacity to be ‘mobile’ in public space, both physically and socially, thus has a clear gender dimension. This was illustrated in our study through a comment made by an older Turkish woman in Brussels who avoided a particular place as she was worried that men would spread gossip about her, which could damage her reputation in the community: ‘I only go shopping in the shop at the corner; I don’t go to [the supermarket] because I have to pass that square then where all the men are’ (63-year-old woman). [Buffel 2017; U]
Friends and neighbors were also identified as other important sources for networking and support. (p.26) [Luo 2016; U]	One male senior’s fishing buddies frequently offered him transportation to the grocery store and doctors’ appointments.<br>Most elders’ friends, however, were the same age they were. They might gather to play mah-jong if they lived within walking distance or in the same building. However, they were hardly able to help one another meet tangible and practical demands, such as transportation or translation of English mail into Chinese. [Luo 2016; U]
The third discrepancy about the familial ideal and immigration reality the interviewees needed to work out was the generation gap. Mrs. Liu explained how she went to Chinatown everyday in order to break the isolation once her role as caregiver was less in demand by the children and then by her sick	I had a monthly metro pass before, but the commute was still one hour one way from Ville St. Laurent to Chinatown. When it snowed, my daughter asked me not to go. She couldn’t stop me once she left home for work [laugh] …The application to Yi Kang [the semi private housing] needed a signature from

husband who eventually passed away: [Hsu 2014; C]	the family. I begged her to endorse my application (Interview, 6 Jan 2009).<br>Mrs. Liu then emphasised the generation gap in her choice of living alone: I am doing well here. I cook whatever I want. If I don't cook, I get food from downstairs...I am very satisfied with my life here. I am happy after living alone. Why? Young people cannot live with the elderly. For example, every Saturday they get up at 11AM. I get up at 5 or 6AM! I already had two meals by the time<br>they are awake. Should I prepare their meals? I don't need to cook for them here (Interview, 6 Jan 2009). p.339 [Hsu 2014; C]
Monolingual seniors found Chinatown attractive not only for convenience and autonomy but also for sociability and daily normalcy. This further required them to negotiate the space of everyday life in Chinatown. The grandiose Chinese arches might be important for tourist development or urban diplomacy with Shanghai, but They played a trivial role in Chinatown residents' life. In contrast, social and exercise space in Chinatown was important for them to cultivate a sense of belonging, social relations and individual wellbeing P.339.<br>In addition to public or semi-public areas, the respondents in this research also took advantage of common areas in subsidised housing complexes and Chinese organisations. They socialised, took language or exercise classes and participated in leisure activities such as Mahjong, Tai chi, choirs and Chinese opera, among others. P.340 [Hsu 2014; U]	Despite the accelerated development for tourism, Montreal's Chinatown continues to provide a vibrant social life for seniors. A long waiting list of 4 years on average did not prevent monolingual seniors from applying for a subsidized one or two-bedroom apartment in one of the five Chinese-run housing complexes. Construction began during the 1980s to accommodate the rising amount of monolingual single senior dwellers. About 400 units reduced the number of seniors living in deplorable rooming conditions. Nonetheless, these seniors did not confine themselves to their apartments. It is common to see Chinese seniors using hallways or food courts in nearby public buildings and shopping malls for socialisation. They gather together to chat, to play Chinese chess or simply to sit alone reading community newspapers. It is<br>equally common for them to spend a whole day in the YMCA in the Guy Favreau building. While some fitness classes are specifically designed and instructed in Chinese, Ping Pong and badminton courts are popular among older immigrants.<br><br>Mrs. Yip recounted her everyday life:<br>I can walk to every corner in Chinatown. Friends or relatives take me out of town for BBQ or to the countryside on the

	weekends…I go to the YMCA every day. Tuesday and Thursday I study [French] at the elderly club. I also stay there for extra two hours or so just to chat…I volunteer in the Chinese hospital, as I can chat with those who have nobody to talk to in Teochewnese (Interview, 13 Dec 2008).P.340<br>Chinatown was mapped out by Mrs. Yip according to sociability and peer support.Her narrative also singled out how comfort and a general sense of satisfaction were negotiated out of the paradox of autonomy and dependence. Even though she did not confine everyday life to the ethnic quarter, her autonomy from family and children nonetheless depended on ethnic functions in the small ethnic community, ranging from the convenience of grocery shopping, the ease of mobility, the availability of peer support, to meaningful habitual practices. [Hsu 2014; U]
It is important to note, however, that despite such pressures, most participants commented on how they strived to transform their current physical environment into a meaningful place; a place that comforts them, where they feel at home and to which they have become attached to varying degrees. Such a sense of local attachment to place was especially evident in people's narratives about memories and experiences that had accumulated about their neighbourhood, with two recurring themes emerging across all study areas: first, the proximity of members of the own cultural community which offered opportunities for developing social networks and realising common social bonds; and, second, the proximity of (ethnic) amenities. (p.21) [Buffel 2011; U]	I like this neighbourhood. My children have grown up here, and I know everyone here; they are like family. All my dreams lie here (Moroccan woman, Brussels).<br>It's a good area… because everywhere is pretty close, you know, especially the community and the mosque and that's the important thing (Somali woman, Liverpool).<br>The best thing about living here is that there is a large Turkish community. I don't feel like a stranger here, we are all the same (Turkish man, Brussels). It is the only other place I've known as home other than Pakistan. So I would say that I feel very at home in this neighbourhood. Your home is what you make, not what people think it to be (Pakistani woman, Manchester) [Buffel 2011; U]
Many older Moroccan women also made reference to a particular park which they regularly visited with their grandchildren and where they met with other women. The park was seen as a place where they could escape from the social control of men. The	A Pakistani woman in Manchester, for example, argued<br>"We need somewhere for the men to get together and a place for the women to get together. We need a lot more services and

importance attached to such “female spaces” was also evident in comments about a lack of meeting places in the neighbourhood. (p.25) [Buffel 2011; U]	facilities for us elderly people here.” [Buffel 2011; U]
Participants primarily ventured out into the community during the warmer seasons. These trips allowed participants to explore their neighbourhoods, seek socialization opportunities, and engage in health promotive occupations. It was often during community mobility that participants were exposed to aspects of the new culture that allowed them to learn about Canadian norms and customs. Simply being amongst others at places of congregation, such as mall food courts and bus terminals, alleviated and distracted from the feelings of loneliness and isolation. (, p. 167) [Wijekoon 2018; U]	For years now, I ride the bus to purify my mind and to see the country. To erase that [loneliness and isolation] I spend time at crowded [bus] stations. [I observe] the patterns of behaviours of different people, listen into the various conversation that happen on the bus and, even in my age, I watch the romantic worlds of young people – a freedom that you do not find in Sri Lanka. So, I go to those [places] to lighten my inside. – Ranil (p. 167) [Wijekoon 2018; U]
Alone and experiencing racism in social housing… values residence’s proximity to local churches [Brotman 2017; U]	“Chan Mi has been able to secure a suite in a low income housing unit (Habitations à Loyer Modique, or HLM). She is very appreciative of the convenient transportation system located nearby which allows her to reach various churches and markets that maintain her social connection and independence. Recently, however, she has been distressed by the deafening noise from the community room next door in her HLM. She told us through an interpreter that her physical and emotional health has been affected “to the point that [her] life has been in jeopardy.” Though she has spoken numerous times to the perpetrator, she faced blatant racism, and felt unheard and ignored. To address the situation, she wrote a formal letter of complaint to the administration office, which she had to have translated into French from Korean, imploring the office to resolve the noise and move her to a unit farther away. These communication efforts are difficult in light of her limited capacity to speak French or English. This attempt and the many others following have not yet made a change which has left Chan Mi frustrated and exhausted. However, she has

	not given up; she continues to actively resist the racism and exhibit resilience in her determination to stop the noise." (p. 65) "Today, Chan Mi lives in a low income housing unit which has a convenient transportation system nearby. Though her home is subject to significant noise (to the point of Chan Mi lodging formal complaints), she values her residence's proximity to her local churches. As a single woman without children, Chan Mi finds strength and stability through her Catholic faith "because God is everything. Everything is from God." She has a strong social network of friends at church with whom she shares "story [about] how they've healed." She keeps herself healthy by going to mass every week and monitoring her food intake. She exercises at the community center gym and frequents the local markets on her way home from church." (p. 36) [Brotman 2017; U]
About half of participants were consistent with calculated accessibility in their census tract. Arabic-speaking participants largely referred to the Arabic Community Centre of Toronto (ACCT) in Scarborough as their primary hub for Arabic services (AF3-P3, AF3-P5). [Dorkenoo 2021; U]	**For instance, one participant (AF3-P3) stated, "I went to the Arabic Community Centre in Toronto... what I like is that the services provided are for free and this makes my life easier." This participant had "high" access in their census tract. A similar sentiment was shared by Spanish-speaking participants for the Centre for Spanish-speaking Peoples in North York (SF3-P6, SF4-P1). These are two major cultural hubs for these communities. Smaller community centres were seldom referred to by name by participants. This indicates that the level of services offered at ACCT and the Centre for Spanish-speaking Peoples were favoured by participants, and they held other centres that may be in their area to a higher standard as a result. This could discourage the use of closer services by participants in their census tract, and prompt them to travel further. This was the case with a Spanish-speaking participant (SF3-P2) who, although they had "good" access,**

	**regularly traveled from Scarborough to the west end of North York to utilize services. These cross-tabulation results reveal the underlying issues of competition and attractiveness of community centres, or any social support service, that impacts why a participant may want to utilize a service and how far they are willing to travel to reach it. (p. 45) [Dorkenoo 2021; U]**
In the first years of Samantha's immigration, her family lived in the south part of the city. She explained that she liked the neighbourhood they were living in. A Catholic church, mall, and nice parks were all within walking distance from their house. Samantha mainly spent her weekdays taking care of her grandson. She felt happy and socially connected in the neighbourhood, mainly through her church: [Schuster 2019; U]	"And... I had more friends [...]. So, I was going with them to church. My church was just two blocks from the house. [...] It was very nice. Certainly, I am missing that." (p. 55) [Schuster 2019; U]
The interviewees' reported social participation in the neighbourhood varied, and included activities such as meeting for coffee, playing bingo, practicing yoga and being busy in an allotment garden. [Jagroep 2023; C]	The interviewees indicated that these activities provided opportunities to be physically active and socialise with other people and functioned as a platform for the meeting of new people and making of friends. They also stated that such activities provided opportunities to remain engaged with and informed about their neighbourhoods.Older Surinamese adults reported that it was their own responsibility to maintain inclusion in the community by going to these neighbourhood activities, as it had a positive impact on their well-being. Most of the activities mentioned took place in interviewees' own neighbourhoods." (p. 6) [Jagroep 2023; C]
A supportive social environment consists of the psychosocial domain ("knowing people," having a place to volunteer and connect), the environmental domain ("the bus is near," "temple is near"), and the cultural (a culturally familiar place of worship, where her first language, Punjabi, is spoken). (p.643)	**These few blocks, these are my village. Because I know those people. [The] bus is near. And my temple is near. [When] I'm not feeling good I go there. And on Sunday I go and volunteer there . ... When we bought this house, we thought the gurdwara (temple) should be near—every**

They get out the door and engage with their local community because they have access to activities, shops, services, and social or religious gatherings that are culturally familiar.(p.644) [Tong 2020; U]	**weekend we should go. (p.643) [Tong 2020; U]**
Geographic proximity to places that provided opportunities for community engagement such as libraries, cultural centres and community centres was reported to help reduce social isolation. [Fang 2016; U]	One person revealed that “the main reason I chose to live here is because it is close to places that I always go to. [Fang 2016; U]
Although the study participants cited Riversdale for its history, and in some cases denser communities of Chinese-Canadian older-adult residents, the participants also explained that the area lacks the institutional and organizational support characteristic of larger enclave communities one might find in big cities like Vancouver, Edmonton, Calgary, Toronto, or Montreal. [Herman 2021; U]	What I am thinking when I came here, everybody said that there is a Chinatown here on 20th Street [the commercial main street of Riversdale]. But actually, no! Only the several Chinese running the business there. Not the Chinese gathering there, it's important. Cultural gathering. Food gathering. Grocery Gathering. Activity gathering. Yah, it [would be] an ideal condition. (Participant 19: Female, 55–69 years old) [Herman 2021; U]
This highlights a failure in services being created alongside awareness of these services increasing. Unfortunately, there is a failure to inform participants of what is available in their area (p. 45) [Dorkenoo 2021; U]	An Arabic-speaking participant stated, “I sincerely don’t know any organizations in my area that provide [community] services,” despite a “good” (z = 0.99) calculated accessibility score in their census. Here, the calculated score and perception conflict with each other. [Dorkenoo 2021; U]
The participants revealed that the activities that they preferred, such as Tai Chi and square dancing, were mostly clustered in the city parks on the Gold Coast. The participants’ residences were scattered across the Gold Coast, which meant that few of the participants were able to visit their preferred parks. [Gao 2020; C]	As indicated in Figure 3, one-fifth of the participants performed Tai Chi within 7 days before the interview. Two participants, Dong and Han, a couple, played traditional Chinese instruments in the park near their homes… Figure 5 shows the travel maps of the 18 participants within a 1,000-m zone highlighted on each map. It showed that the participants’ behavior in visiting parks and their travel tracks depend on the distribution of parks within a 1,000-m zone (see Figures 5A–C,E,F). They could walk to the parks near their home, which indicated that the accessibility of parks could partly meet the

	participants' needs. However, they had lower accessibility to their preferred park." (p. 6) [Gao 2020; C]
However, some older Surinamese adults indicated that they travelled long distances to community centres where activities were organised especially for older adults with Surinamese backgrounds (e.g. playing Surinamese games, singing traditional songs). [Jagroep 2023; C]	The interviewees indicated that taking part in cultural activities, such as Deepavali (the festival of lights) or Surinam's Independence Day, was important to them, and that they travelled outside of their neighbourhoods when necessary to do so. (p. 6) [Jagroep 2023; C]

Racial discrimination, ageism, neighborhood deprivation, and linguistic barriers result in reports of poor social cohesion. Positive neighborhood perceptions were characterized by strong social cohesion via trust, reciprocity and sense of belonging which was facilitated by having lived in a neighborhood for a long time, knowing one's neighbors well (irrespective of cultural and linguistic similarities), and the presence of co-ethnolinguistic community.

3.1 Safety and neighborhood deprivation:

Author Statement	Illustration
Some participants reported territorial exclusion, such as a reduced geographic living area and an unsafe neighborhood. A few participants felt lucky to live in Los Angeles because of a high-density Chinese population. However, some participants also reported that they sometimes feel unsafe in their own neighborhood. (p.10) [Xu 2023; U]	For example, **P2 (Female, 74) mentioned that the senior apartment she lived in used to be safe, but not anymore, because a TV set was stolen from a common room in the basement.**<br>**Sometimes, there are people who want to break in. There is no safe place nowadays. It depends . . . there is no safe place. Even now you go onto the street, older people need to be very careful when you go to the market to buy stuff. You need to carry your handbags on your shoulder, don't lift it [in your hand]. Otherwise, bad guys will rob you. (P18, Female, 69) [Xu 2023; U]**

More significant element of the accounts given by Caribbean participants of their localities, which were around crime and fear of crime.(p.556) [Becares 2013; U]	**Oh yeah, we had a stabbing. And they had a shooting. Yeah. Drug related things… Otherwise it's not too bad … I didn't know about the stabbing until I saw it on the telly and it was two houses from me. And the shooting thing, I was pulling the curtains in the front room and I saw all these Policemen out there down on their hands and knees and what not, and I thought 'What's going on?' so I saw [Friend] in the yard and I asked him what happened. And then he told me that somebody shot somebody and they were looking for the bullets and what not.**<br>**I thought 'Oh' … you sort of feel scared really in'it, to see something like that happen so close to home, you know. (802, Caribbean women in her mid-60s) [Becares 2013; U]**
Such account was not were not present in the Indian participants' interviews, where mentions of experience or fear of crime were coupled with characterisations of the local area as nice. [Becares 2013; U]	The area is very nice and the people are very nice. Our neighbours opposite who are white are very very nice people. If I call them at 12 o'clock at night they will come. The kids who Live opposition is very bad. They are youngsters and they are very bad children ... They are always creating problems. So now it is all right. They broke our glass in the window. (588, Indian woman in her early 60s) [Becares 2013; U]
Apart from the emotional bond and belonging, the cultivation of a SOC [sense of community] also encompasses the overcoming of negative experiences…<br>For Tian, this incident represented a significant event that ruptured his sense of belonging in the neighbourhood…<br>Tian's narrative suggested that negative experiences and incidents could provoke fears and emotional insecurity (Brodsky, 1996). Consequently, his positive emotional connection with the neighbourhood was eroded. It also showed how broader negative discourses around migration can shape local	In the following extract, Tian, a 69-year-old man, described his negative relationship with the neighbourhood at the time when eggs were thrown at his daughter's car. When the first author arrived at Tian's home for the second interview, Tian was cleaning up his daughter's car and told the first author:<br>Kids threw chips and eggs at my daughter's car. It's frightening. I will remind myself to be careful in the future. For example, lock my doors and windows when I go out, and not to walk closely to a stranger. [Li 2014; U]

actions, in this case leading to the throwing of eggs. (p.31) [Li 2014; U]	
Hong's account describes a disruption to her positive relationship with the neighbourhood, while illuminating the impact of negative experiences on older Chinese migrants. All people may experience negative neighbourhood interactions at different times. Older migrants are more likely to spend time in their neighbourhoods, partly because their limited social networks and English language abilities constrain their social lives. As a result, they may suffer greater exposure to stressful neighbourhoods, especially when they become the targets of attacks.<br>(p.31) [Li 2014; U]	Such experiences were evident in other participants' accounts. For instance, Hong said<br>Several of us were attacked by kids in our neighbourhood. Some girls threw eggs at us. A group of young men threw stones at an older Chinese couple who were waiting by the bus stop near their home. The couple had been afraid to leave their home since then. We didn't feel safe when we walked on the street even at as early as seven o'clock in the evening. [Li 2014; U]
Through the culture of civility, powerful and positive sentiments were produced in addition to strong emotional connections with the community where participants aged. Through such neighbourhood, building a SOC is cultivated. As such, SOC does not just happen; it requires collective work that establishes relations between peoples and the community and significantly expands inter-subjectivity beyond the person to the neighbourhood, community and society as a whole. Research suggests that neighbourhoods where negative experiences occur are as significant as neighbourhoods where needs are met and succour is found in terms of their ability to influence residents' wellbeing, particularly when people put an effort into turning a negative neighbourhood into a positive one (Manzo, 2005).<br>(p.31-32) [Li 2014; U]	Ming, a 76-year-old man, reflected how older Chinese immigrants and the larger community collectively worked to improve their neighbourhood. This invoked processes of bonding in opposition to discrimination and the threat of crime:<br>We organised an informal meeting to discuss our worries about our safety. Most victims of the attacks didn't report to the police. Inability to speak English and the belief that the police would do nothing stopped them from reporting the attacks. We decided to act collectively. We approached newspapers. The reporters disclosed the attacks. The police then set up a call centre for Asians in our community. A Chinese-Kiwi Friendship Programme was also established to help Kiwis and the older Chinese to better know each other and to keep our neighbourhood safe together.<br><br>Ming portrayed a picture representing a 'culture of civility' (Godfrey, 1988), where residents regarded multicultural diversity as a civic resource and not as a dangerous threat. For example, the police initiative offered older Chinese people a place where they felt safer. The Chinese–Kiwi friendship

	programme provided a platform to foster friendship, to share experiences and for cultural exchange. These collective efforts based on civility and mutual interest provided a model for settling neighbourhood conflicts and contributed to the construction of a SOC and a sense of empowerment for those Chinese elders who inhabited these multicultural contact zones. [Li 2014; U]
Among other interviews, also provided insights into some of the structural barriers, which prevented some older migrants living in deprived areas from engaging in informal and formal social relationships and from creating a sense of home. Poverty, poor housing conditions, language barriers, perceived vulnerability to crime and lack of access to services and facilities were among the factors which discouraged some people from engaging in community life. (p.26) [Buffel 2011; U]	These spatial conditions – the situational contexts of older migrants' lives (Becker 2003) – figured prominently in their comments on how they experience place: Basically I can only describe this area as an area that has been forgotten by the authority. It is an area where there are a lot of drugs and violence and it seems the situation is getting worse…There are always children doing anti-social behaviour, breaking windows and throwing missiles against elderly people (Somali man, Liverpool).<br><br>I live here because the rents are affordable, but it's not a good area: lots of thefts, lots of noise, and very filthy (Moroccan woman, Brussels). You can manage [financially]… I don't go to entertainment; I don't go now here (Somali man, Liverpool).There are no activities here. here is one place where people come together, but I don't know what it is. Language is a major problem. As we cannot speak the language, we cannot understand the relevant information (Turkish woman, Brussels). [Buffel 2011; U]
An unsafe community environment was identified as a barrier for transportation. [Dabelko-Schoeny 2021; U]	There are some neighborhoods, which are difficult neighborhood, like having drugs, booze, violence, gangsters. There is that ... especially drug neighborhood. (Somali). (p. 7) [Dabelko-Schoeny 2021; U]
Participants indicated that it used to be common to introduce yourself to the neighbours, but not currently, which has	Older Surinamese adults indicated that this was essential to know the neighbours, for example, in case of an emergency or to share

negatively affected the social cohesion of the neighbourhood. [Jagroep 2023; U]	information about the neighbourhood. The interviewees had mixed experiences with respect towards older adults in their neighbourhoods. For example, addressing someone regarding their behaviour was not always appreciated: They [boys at the bus stop] started berating me. Say some words that you cannot say. I was with my walker and looked at them, but I said nothing. Otherwise, they would beat me up. Yes, I am afraid of those things. (Participant 8) (p. 7) [Jagroep 2023; U]

3.2 Racial discrimination and ageism:

Author Statement	Illustration
"Social distance" with people from "Other Group"<br>A common experience was that the participant would recall that when their children were young, they knew neighbors who also had school-aged children. Over time, households would relocate as children moved out. The newer neighbors might be working aged, away during the day, busy with their own young children, and less available for intermittent neighbourhood socializing. Sometimes, the lack of familiarity caused uncertainty or insecurity. People's discomfort was frequently a result of perceived social distance from the neighbours, being far apart in age or of a different ethnicity.(p.05) [Yen 2012; U]	"Cause you know Chinese people don't. . . Some of them don't talk to Black people. And that was unusual for them when I was walkin' the hall she spoke to me and talked to me and asked me how I was doin". And I was just surprised that she would talk to me.(p.05)<br><br>A man (70, Latino) commented on young people hanging around in the neighborhood, giving him a feeling of insecurity: "In the outskirts of our neighborhood there's been more kind of young kids congregating on corners. On the business district there's more young Black and Latino kids, maybe sometimes a White kid, too, but Black and Latino kids, kind of acting rowdy, loud." Later, in response to a question of whether there was anything he did not like about his neighborhood, he added: "What I don't like is the sense that it's become a little more dangerous, you know, in terms of reading about assaults, and seeing kids acting out, you know, on the street. You know, fifteen year olds, acting crazy".(p.05) [Yen 2012; U]
There are different cultural values in Chinese and American society, which can contribute to	**For example, based on the participants' understanding or expectation, social**

both perceived and experienced social exclusion among Chinese American older adults. (p.10) [Xu 2023; U]	**relationships with neighbors are closer in China, while in the U.S. these relationships are more distant. This may make older Chinese immigrants feel it is difficult to integrate into the neighborhood. For example, P2 (Female, 74) mentioned, "I've been here for five years.**<br>**Neighbors don't interact with each other very much. Americans are like this". Similarly, other respondents identified this: I lived in three or four places [and my] relationship [with my neighbors] was only a greeting. . . . People come out of their homes at the same time. You don't look at me and I don't look at you. That is, an interpersonal relationship is the only greeting. (P12, Male, 68) [Neighbors] They have their own life . . . do their own things, have small family . . . . This is American style . . . . You don't bother people and they don't bother you.**<br>**Americans are like this, which is different from our Chinese values. People help and support each other in traditional Chinese value . . . a system of big family. (P21, Male, 68) [Xu 2023; U]**
Exclusion from the neighborhood is also seen in older Chinese immigrants' absence of a close relationship with neighbors. Participants feel excluded from the neighborhood they live in, especially when where there are a large number of Westerners (p.10) [Xu 2023; U]	Some Chinese people feel low self-esteem when they go to westerners' neighborhood, or they feel different from them . . . I heard from my friends sometimes that if there is only one or two Chinese [in the neighborhood], they live very uncomfortably. (P17, Female, 65) [Xu 2023; U]
The lack of safety in the neighborhood could also be related to racial discrimination (p.10) [Xu 2023; U]	A batch of people lives nearby our house. They are Whites, so they look down on us. They sometimes throw some plates to my roof, climb up on our roof, and step on our house. (P10, Male, 75) [Xu 2023; U]
Participants described a range of experiences with their neighbours, from detachment to friendships. Having detached or limited social relations with neighbors was common. In general, they expressed satisfaction with the way things were. At times, people noted that over several years, the turnover in neighbors had created a situation where they were not	I don't know anybody who lives up here. We did know somebody who lived up here, but they moved away, so I guess we don't know anybody else. I know [name], who lives right behind us. . . . The other people I just wouldn't recognize if I bumped into them on a street. (p.4)

familiar with their neighbors. In these instances, they also pointed out that the newer neighbors were working age and busy during the day. The differing schedules between the older adults and the working adults meant lower likelihood of running into each other coming and going.<br>[Yen 2012; U]	"Yeah, and so I enjoy them, because it's like I said, they don't bother me and I don't bother them. . ."(p.04) [Yen 2012; U]
Groups of young people were seen as a threat or a nuisance. There was a sense that older adults and young people belonged to separate groups while occupying the same space. People mentioned a sense of vulnerability in part due to being older with less capacity to defend oneself. [Yen 2012; U]	A man (70, Latino) commented on young people hanging around in the neighbourhood, giving him a feeling of insecurity: "In the outskirts of our neighborhood there's been more kind of young kids congregating on corners. On the business district there's more young Black and Latino kids, maybe sometimes a White kid, too, but Black and Latino kids, kind of acting rowdy, loud. Later, in response to a question of whether there was anything he did not like about his neighborhood, he added: What I don't like is the sense that it's become a little more dangerous, you know, in terms of reading about assaults, and seeing kids acting out, you know, on the street. You know, fifteen year olds, acting crazy".(p.05) [Yen 2012; U]
Hong's account describes a disruption to her positive relationship with the neighbourhood, while illuminating the impact of negative experiences on older Chinese migrants. All people may experience negative neighbourhood interactions at different times. Older migrants are more likely to spend time in their neighbourhoods, partly because their limited social networks and English language abilities constrain their social lives. As a result, they may suffer greater exposure to stressful neighbourhoods, especially when they become the targets of attacks.<br>(p.31) [Li 2014; U]	Such experiences were evident in other participants' accounts. For instance, Hong said<br>Several of us were attacked by kids in our neighbourhood. Some girls threw eggs at us. A group of young men threw stones at an older Chinese couple who were waiting by the bus stop near their home. The couple had been afraid to leave their home since then. We didn't feel safe when we walked on the street even at as early as seven o'clock in the evening. [Li 2014; U]
Given the feeling of Outsiderness, participants stressed that relations between communities in Southeastern Wisconsin needed to improve.<br>[Hawkins 2022; U]	When answering the aforementioned questions, Participant 12 (98 years old) said that the relationship between the Spanish-speaking and non-Spanish-speaking communities needed to improve, through,

	“more understanding, more communication, if there is no communication, there is nothing.” This was echoed by sentiments of a lack of community cohesion, which contributed to feelings of Outsiderness. Participant 10 (76 years old) said that she would have more support in a more united community, “with a united community, yes, but here no, I don’t see that...I don’t know, everyone is doing their own things, I don’t know, this union, no (isn’t there).”” (p. 117) [Hawkins 2022; U]
In Canada, participants were deprived of the informal daily social interactions and pleasantries that took place between neighbors in their home country. In describing this lack of regular interactions, which did not provide sufficient opportunities to ‘use’ his mouth, Saman stated “I have cobwebs in my mouth from not talking”. Specifically, Canadian culture was perceived to promote privacy and social distance between persons in society. Visual markers such as closed doors of neighbours’ homes, in contrast to the open-door policy of Sri Lanka, led to participants’ perceptions of their Canadian neighbours as uncongenial, unconcerned, and unwelcoming. These perceptions reinforced participants’ sense of exclusion in their neighbourhoods and communities…<br>The social distance and privacy of Canadian culture engendered a feeling of anonymity and invisibility within their neighborhoods.” (p. 166) [Wijekoon 2018; U]	In Sri Lanka, we spoke to all our neighbours. They come over [to our house], we go over [to their house]. That does not exist here. A neighbor does not know whether there is an ill person next door, whether there is an argument, or whether there is an assault. What happens in this house is not known by the neighbor next door. That is the nature of this country. As I said before, the culture of Sri Lanka operates according to a certain method. You are encouraged to give a helping hand to anyone in your vicinity. There is no such thing in this country. If there is a person on the street, they do not even look you in the eye. At least in Sri Lanka, if you speak to someone they would say ‘ok bye’. There is no concern for your neighbor, whether it is genuine or insincere. Because of that we do not have a relationship [with the neighbours]. – Ranil (p. 166) [Wijekoon 2018; U]
The social distance and privacy of Canadian culture engendered a feeling of anonymity and invisibility within their neighborhoods. [Wijekoon 2018; U]	In speaking about their inconspicuousness, Kamal expressed “Halloween...during that time only we are moving with the neighbors. During that time, they smile and are happy about us. That is when they get to know we are from this place [this neighbourhood]”. (p. 166) [Wijekoon 2018; U]

Noting the cultural dissonance, participants abandoned familiar ways of connecting with people, and strived to adopt the perceived Canadian values of maintaining a social distance between themselves and their neighbors. [Wijekoon 2018; U]	For example, Geetha spoke of her hesitation to share her homegrown vegetation with neighbors. Even if I have a tree bearing fruit, even [if they are] vegetables that we grow in our backyard, we can't share [with the neighbours]. We are scared to give it to anyone. There's a grape vine. But I don't give anyone because they may not accept [it]. – Geetha" (p. 167) [Wijekoon 2018; U]
The study participants indicated that they were infrequently involved in decision making about their neighbourhoods, which they viewed as a pity, especially as they were getting older and had different needs: [Jagroep 2023; U]	And we older adults we grow older. What we need is, to live as comfortably as possible. You notice it when you get older, you will get other needs. (Participant 1) They mentioned that they would like to raise some points about neighbourhood issues, but that they did not know how or where to do so" (p. 10) [Jagroep 2023; U]
Some interviewees felt that such raising of issues was pointless because they had already been 'written off': [Jagroep 2023; U]	We [the participant and partner] have made some suggestions in the past, but they did nothing with them. People from the municipality do not even look at it I guess. They have their own ideas. (Participant 5) (p. 10) [Jagroep 2023; U]
Most participants arrived in the 1940s–1960s, through different migration routes, largely differentiated by nationality: from Ireland as labour migrants, from the Caribbean as British subjects, many Polish interviewees were WW2 refugees; a few arriving through marriage visas. Upon arrival, most participants encountered new localities as strange, unfamiliar and often hostile. Participants from the Caribbean and Ireland, in particular, recounted incidents of discrimination, especially in relation to employment and housing. Many of these participants described seeing the infamous signs 'No Blacks, No Irish', especially in boarding housing in the 1950s–1960s. (p.05) [Ryan 2021; U]	Henry from Jamaica recalled: 'you remember, no Irish and no dog and no Blacks'. Similarly, Ronan, an Irishman now living in Yorkshire, recounted his shock at first seeing these signs in London, his original destination: 'you walk past a house and you might see 'vacancies' but "no Blacks, no dogs, no Irish". That was shocking … terrible, hurtful.' Hannah, originally from Guyana, recalled: 'There was a lot of racism … Accommodation was horrible, you couldn't get accommodation. You have doors slammed in your faces: "No coloured".' Our Polish participants were not immune from discrimination. Mandek, a Pole who arrived in Britain in 1947 and trained as a coal miner, explained that during the post-war period, there was considerable hostility towards Polish workers: 'obviously they wouldn't take any

	Poles or foreigners'. Marjorie, from Jamaica, remembered people shouting: "Get back, you blackie, to your country" and two fingers would go up.' Howard, from Guyana, described a racially motivated physical assault in London in the 1960s when he was left with several fractured ribs. However, like many participants from the Caribbean, Howard suggested that racism had changed over time: 'you get racism… all the time … But thankfully, now it's not as bad as it used to be'.(p.05) [Ryan 2021; U]
Surinamese Creole participants indicated that they had felt discriminated against in the past due to their Surinamese backgrounds, but that the multicultural compositions of their neighbourhoods contributed to their feeling of connectedness with their neighbours:" (p. 7) [Jagroep 2023; U]	A lot of people from the first batch are gone. Now I do not notice any discrimination here anymore. More migrants have also come to live here. The Dutch have made way for the migrants. It is also easier to make a connection. (Participant 3) (p. 8) [Jagroep 2023; U]
Alone and experiencing racism in social housing… values residence's proximity to local churches [Brotman 2017; U]	"Chan Mi has been able to secure a suite in a low income housing unit (Habitations à Loyer Modique, or HLM). She is very appreciative of the convenient transportation system located nearby which allows her to reach various churches and markets that maintain her social connection and independence. Recently, however, she has been distressed by the deafening noise from the community room next door in her HLM. She told us through an interpreter that her physical and emotional health has been affected "to the point that [her] life has been in jeopardy." Though she has spoken numerous times to the perpetrator, she faced blatant racism, and felt unheard and ignored. To address the situation, she wrote a formal letter of complaint to the administration office, which she had to have translated into French from Korean, imploring the office to resolve the noise and move her to a unit farther away. These communication efforts are difficult in light of her limited capacity to speak French or English. This attempt and the many others following have not yet made a change which has left Chan Mi

	frustrated and exhausted. However, she has not given up; she continues to actively resist the racism and exhibit resilience in her determination to stop the noise.” (p. 65) “Today, Chan Mi lives in a low income housing unit which has a convenient transportation system nearby. Though her home is subject to significant noise (to the point of Chan Mi lodging formal complaints), she values her residence’s proximity to her local churches. As a single woman without children, Chan Mi finds strength and stability through her Catholic faith “because God is everything. Everything is from God.” She has a strong social network of friends at church with whom she shares “story [about] how they’ve healed.” She keeps herself healthy by going to mass every week and monitoring her food intake. She exercises at the community center gym and frequents the local markets on her way home from church.” (p. 36) [Brotman 2017; U]
While most felt reasonably comfortable with ambiguous boundaries and had achieved a certain degree of feeling at home in both places, the ways in which participants negotiated their multiple sense of home also revealed expressions of ‘placelessness’ among some. [Buffel 2017; U]	Such a sense of exclusion was evident, for example in the following comment made by a man:<br>In Turkey we are viewed as Europeans, and here we are viewed as foreigners’, this reflecting his feelings of not-fully belonging to either place (see also Bolzman, in this issue). Further in the interview, he explained that ‘European Turks’ are often portrayed as ‘gâvur’ by Turks in Turkey, a derogatory term denoting infidelity or immorality:<br>‘Our village has also changed. The people… I don’t know why, but it feels less welcoming. They are less sincere perhaps. When we arrive in the village, they point at us: “look, the gâvurlar”’ (67-year-old man). (p09) [Buffel 2017; U]
I noticed that Samantha compared her life in the old neighbourhood with her life in the new neighbourhood. She expressed her sadness about having left her church and her friends from the south behind. Although she liked her	“[...] most of the people here are young people with kids. And my relationship with them is just through the kids. [...] But in the other neighbourhood, I had a lot of friends [...]. I think I was maybe having a better life because

current neighbours, she said that she has difficulties finding friends among them: [Schuster 2019; U]	[...] there were many things nearer." (p. 58) [Schuster 2019; U]
The only social contact they had outside of their family were with people who they connected with through their family. However, often they did not consider these friendships to be particularly meaningful. [Schuster 2019; U]	"Not very great social relations. Because [...] most of the people here [in the neighbourhood] are young people with kids, and my relation with them is just through the kids." (Samantha)" (p. 79) [Schuster 2019; U]

3.3 Linguistic barriers

Author Statement	Illustration
Those who continue to reside in the remnants of Saskatoon's spatial ethnic enclave may have a lower degree of cultural and linguistic integration, part of the continued impacts of past and present systemic discrimination. A participant reflected that while younger generations of Chinese-Canadian residents seem to rely less and less on the confines of a Saskatoon-based spatial enclave, older adults are often among those remaining. [Herman 2021; U]	I think [Saskatoon is] pushing at the Chinatown concept, it's been deteriorating. It's simply that younger people move in and they move over. They don't concentrate in one area like in the old days when the immigrants gathered in one place for communication purposes … they would gather in one place so they can get along and help each other. But now that they're settled, the young people learn to fly. (Participant 18: Male, 70–84 years old) [Herman 2021; U]
Chinese-Canadian older adults who continue to prioritize residence in Riversdale, despite its lack of age related institutions, noted that they do so primarily for the opportunity of proximity to linguistically and culturally homogeneous social networks. Particularly For those with increased indicators of linguistic and cultural difference from the larger Saskatoon community, Riversdale remains an important social resource in older age. [Herman 2021; U]	So he … came here [Riversdale], because this is the Chinese area and he lives here. And other public spaces in this neighbourhood, he [has] never go[ne] there before. (Stated by interpreter, Participant 13: Male, 55–69 years old) Because his English is not very good…even though he is working full time, he cannot communicate with his colleagues and his co-workers … he cannot imagine a life without the Chinese neighbourhood here. (Stated by interpreter, Participant 14: Male, 55–69 years old). [Herman 2021; U]
By contrast, those participants who were longer-term or native-born residents of Saskatoon were less likely to place the same emphasis on residential proximity	They still gather around, and they feel more comfortable too I believe, more at home … in the company of their own people. (Participant 18: Male, 70–84 years old) People have some

to Riversdale. Though less reliant on the linguistic support of day-to-day spatial proximity to other Chinese-Canadian residents, a broader social network of older-adult peers remains fundamental for access to important age-related institutions and recreational activities. As was broadly evident in our research, the city's Chinese-Canadian community itself remains the key source of social connection, entertainment, and general age-related support among many of the group's older adults. [Herman 2021; U]	problems with the English language, [so the] seniors only socialize among themselves, they don't really mingle around with the Caucasians or anything … they always stick to themselves. (Participant 1: Male, 55–69 years old) [Herman 2021; U]
Our discussion begins with housing, in which Saskatoon's Juniper House features prominently. The Juniper House, located in Riversdale, is a community-owned non-profit housing development for Chinese-Canadian older adults. Residents have access to a community kitchen area, outdoor garden, and sizable activities space. The Juniper House offers a culturally and linguistically homogeneous housing environment that operates as the spatial core to the community's social enclave. It is an important resource to many Chinese-Canadian older adults who are seeking additional support in older age, but feel either uncomfortable or unwelcome in more heterogeneous retirement community environments. [Herman 2021; U]	It's hard to have Chinese go to the care home…they don't like it … but they come here [Juniper House], and it's okay. If they come here. (Participant 12: Female, 55–69 years old) [Herman 2021; U]
Although the Juniper House itself offers no direct age related support to its residents, physical proximity among members of the social enclave aids in a number of daily activities and supports aging in place. [Herman 2021; U]	The convenience to live here [Juniper House], [is that] if you wanted to go out and buy something, somebody else will come [to help translate], and you can go with them. And you could help others buy something ... .Sometimes he will ask someone … who wants to go buy things together, and [he] gives them a ride. (Stated by interpreter, Participant 14: Male, 55–69 years old) Some elderly, they live [at the Juniper House] and they always can get help because they are surrounded by Chinese. So if they want help, always neighbours or friends who live around

	here can help them. (Participant 19: Female, 55–69 years old) [Herman 2021; U]
While half of the participants reported being hospitalized within the past year, they have frequently visited bilingual primary care physicians in Chinatowns. (p.384) [Chen 2022; C]	There are family doctors and clinics available in the community. You can immediately make an appointment with them if you are sick. Many of them speak Chinese, and if you are good at English, you can see an English-speaking one. (P12, Male, 70) (p.384) [Chen 2022; C]
Language barriers were the major obstacle that hampered Chinese elders from making connections with people outside the Chinese community. (p.27) [Luo 2016; U]	Some elders expressed that they were open to diverse activities in their apartment buildings or in the city, such as coffee parties, games, or free concerts. However, when they attended the activities, they found they could not understand what fellow attendees, who were Canadian and English-speaking, were trying to communicate with them (e.g., jokes or instructions of games). (p.27) [Luo 2016; U]
Participants who were late-life migrants (all Asian and most Pacific participants) highlighted just how exclusionary not being able to speak English proficiently was in their everyday life. Asian participants lamented the catch-22 that not being able to speak English made it even more difficult to figure out how to get lessons to learn English and their reliance on the pension made it difficult to pay for them. Not speaking English also left late-life migrants in particularly precarious situations when anything happened to their existing social support network: [Morgan 2021; U]	When we first moved here, if there are Korean people we all became friends, then now, since we have been here long and since this [admission to hospital] happened to my husband [we] all disconnected. And besides, there is not a single Korean per-son living around this neighbourhood. No one in this area, I am alone ... there is no one I can talk to. It's very much futile. Living is. It's only my sons, no one else I know. Even the neighbours, I can't talk to them, I can't talk [means she can't speak English]. (AF01) [Morgan 2021; U]
In essence, enhanced ELP decreased feelings of being an outsider. [Hawkins 2022; U]	The impact of lack of ELP was further explicated by Participant 8: "It's bad that I don't know that language." (вот плохо то что я языка не знаю) The lack of ELP increased feelings of isolation, "I am alone" (я одна) – Participant 8. Yet, for participants who had learned English, not at an advanced level, but at a level which allowed them more freedom, they expressed increased comfort going out-and-about, using public transport, and engaging with others.

	"Therefore, I now feel like a fish in water, that is to say, I can (go to) any building... All I have to do is ask for help... Before I was afraid to speak [English]...Now, I feel great in any place, on a bus, in a store, in a hospital." – Participant 3 (80 years old)" (p. 118) [Hawkins 2022; U]
All (100%) the participants expressed the difficulties of interacting with their neighbors because of the language issue. They seldom start a conversation with their neighbors and often do not establish stable and deep relationships, [Gao 2020; U]	**as expressed by Xin (male, 76 years old, living in Australia for 9 years): "I sometimes see some neighbors in the park. But we don't communicate much. It's annoying because I have many words to say but I can't express myself. I have learnt some simple sentences to communicate with them, but that's not enough. It's a pity that we don't have any in-depth communication." (p. 9) [Gao 2020; U]**
They felt that the consideration of cultural norms and values was essential to show respect, as all of them lived in multicultural neighbourhoods. [Jagroep 2023; U]	They noted that in general, older adults with, for example, Turkish or Moroccan backgrounds do not master the Dutch language well, making communication with them difficult. The interviewees indicated that they and such people greeted each other, but could not converse more (e.g. ask how the other is doing), which made connecting difficult. Some interviewees indicated that connecting with younger neighbours with Turkish and Moroccan backgrounds was easier, given their better mastery of the Dutch language: I do feel that it is easier to get in touch with young people than older ones. Especially because the language is a barrier. Older adults who do not speak the Dutch language move a bit in their own group. (Participant 2) (p. 7) [Jagroep 2023; U]
Services for housing and transportation are important aspects of daily life. Some participants reported that they experienced difficulties receiving sufficient services for housing maintenance in their senior apartment, because they had limited ability to take care of	If there is an agent—because people are all old, you know, people like us knowing little English—sometimes it's better to have a person helping us with this [housing stuff], but there is no such person. (P11, Male, 73)

the outdoor spaces of a residence. Moreover, this is exacerbated by the language barrier. (p.7) [Xu 2023; U]	Sometimes my car would break down. It was troublesome when it happened. Because my English is not good—that is, I didn't know how to call [for assistance] and I couldn't find anyone to help. So I stood for very long time and it didn't get solved until the police came. (P10, Male, 75) [Xu 2023; U]
Language may have been a factor in this dynamic around social distance. Another Latina (age 65) was concerned that storeowners in her neighborhood were taking advantage of the residents by overcharging their merchandise. (p.05) [Yen 2012; U]	The only problem we have with the stores, is that almost all are run by Arabs. They are, how can I say this, abusive, because they have checkout machines. The items have very small labels and the prices are always faded so it's not possible to see how much something costs. After you pay, they give you a receipt, but there's no ink, so you can't see what the price was. This is a problem for us. [Translated from Spanish](p.5-6) [Yen 2012; U]
Limitations, such as an inability to drive, has caused a shift toward elders being homebound, engaging in temple participation solely on weekends, and infrequent trips to visit family, friends, or markets because of the need to rely on younger family members who often are working….As her narrative shows, it is more difficult for elders to achieve the level of freedom they expect. Elders, such as the woman described above, limit their interactions with non-Khmers because of language difficulties and what they perceive as suspicious looks from neighbors. The critical aspect of neighborliness and belonging (Casarett, 1991) is missing. (p387) [Lewis 2009; U]	One older woman, living in a small house with her daughter while caring for her young grandson, described how she had lost her freedom when she arrived in the United States. No longer able to walk to the temple, to markets, or to visit other elderly women, she feels trapped. She explained, "I am afraid to walk around my neighborhood. I don't know the people; they only speak English. They watch me when I walk so I am afraid." She stays inside her small frame home and only ventures into her fenced backyard an hour or so each day so her grandson can play. She is as suspicious of her non-Khmer neighbors as they are of her. She explained that, in Cambodia, she remembers her mother walking to visit other women, walking to the market, and feeling free to go into the forest to gather fruits. She is happy that she is in the United States but misses the ability to move about freely. She is acutely aware of a disconnection between her memories of place in Cambodia as connections across relationships and with the land and present-day discomfort with unfamiliar terrain and customs. "Here I have my grandson," she continued, "I make my daughter and her family happy; I pray to Buddha. I am peaceful

	inside [the house] with my small family. Next time [in her next life] maybe I can be free, too."<br>(p387) [Lewis 2009; U]
the overall view of community was moderate because older adults in both the Chinese-speaking environment and non-Chinese-speaking environments could not enjoy the convenience and facilities to their full extent. (p.23) [Luo 2016; C]	Older adults living in an environment that had not been designed to accommodate people from different cultures, although happy with the physical environment, were unable to take full advantage of available services and activities in the community, despite their high quality and quantity, due to language obstacles. (p.23) [Luo 2016; C]
When older monolingual immigrants came to reunite with their families, they stayed in different neighbourhoods, and many of them lived in multiethnic suburbs like Brossard and Ville St. Laurent. Getting into and around the city posed a challenge and exacerbated the sense of isolation. (p.338) [Hsu 2014; C]	Mrs. Tu moved to Montreal in 1981 at the age of 55 years. She expressed that she was frightened at the beginning because she "did not speak the language and could not read road signs." Even though Mrs. Tu stayed with her daughter's family, she repeated that "I wanted to leave [Montreal]. I didn't dare go out alone. I didn't know the roads. I knew nothing at all. It was very difficult at the beginning" (Interview, 2 Oct 2008).<br>(p.338) [Hsu 2014; C]
Other participants echoed this sentiment of enhanced ELP facilitating a greater sense of ability to navigate transport, appointments, and other outings. [Hawkins 2022; U]	Participant 1 took a picture of a bus to demonstrate her appreciation of public transport, and Participant 16 expressed that ELP enhanced her confidence when working with her physician. [Hawkins 2022; U]
Perception of place: although most of the participants mention the poor sanitary, noise, and dense population of, they recognized its safety and convenience. Most important, within the place they lived in, they spoke the same language and dialects, which enabled them to complete daily routines such as grocery shopping, exercising, seeking primary medical service and participating in community activities and cultural events. Many older Chinese immigrants have experienced emotional attachment and security within this place where they	I think Chinatowns are a better place for us to live because we can't communicate with others if we go out. I can't read or speak English, so I will not know where I am if I go out. Living in Chinatown is good; at least you can communicate with each other in Chinese. It's more convenient for us to buy some stuff, and we can chat with the neighbors. I can visit the Chinese-speaking doctor here as well. (P10, Male, 83) (p.383) [Chen 2022; U]

shared a similar cultural background. (p.383) [Chen 2022; U]	
The participants interviewed for the study also commented on how they had worked to transform their current physical environment into a meaningful place, one where they feel at home and to which they felt attached to varying degrees. Such a sense of home was itself both created and reinforced through the proximity of members of their own cultural community, creating opportunities for common social bonds. (p.05) [Buffel 2017; U]	‘I wouldn’t want to [move away from here] because I don’t want to live far away from all the Turkish people. When I go out here I always meet family and friends on the street; and that gives me a sense of relief. I am already in a foreign country… if I would live somewhere far away from the Turkish community, it would feel as if I’m moving to a foreign country for the second time’ (69-year-old woman).(p.05) [Buffel 2017; U]

3.4 Civic Participation and reciprocity

Author Statement	Illustration
Older adults who participated in organized neighborhood civic action felt that their neighborhood provided them with opportunities and support to stay engaged and informed. Regardless of race, gender, and socioeconomic status, residents collectively came together for a common cause such as advocating for neighborhood parks or other structural and/or social resources that would enhance the well-being of the residents. The community’s neighborhood associations provided residents with opportunities to gather as a group and organize. Local leaders often spear- headed the efforts and encouraged individuals to participate. In essence, the neighborhood associations and local leaders were a part of the social network that garnered opportunities for bonding, bridging, and linking to promote civic participation. (p.499-500) [Parekh 2018; U]	A 64-year-old African-American community leader discussed how her “neighborhood came together” for a common cause of importance to resi- dents from her neighborhood:<br>We now have a park as a result of our neighborhood coming together with the [city] Conservation Council to form and make sure that the park was happening because they were going to take it off the plans. And we said, no, we moved here for this park. You’re not taking our park away. So, we now have a park, and we’re excited about it, and we participated. We bought benches for it, and we’ve gone out once a month and mulched and did things we didn’t even know how to do with our green thumbs, our brown thumbs, our striped thumbs, whatever we have. [A focus group participant]<br>According to a long-standing member of a local Hispanic political and social organization:<br>I’m a member of a Hispanic political organization. ... [city] was a little tiny ranch when I first came here. In 1980 the

	immigrants started arriving. ... Everybody started to say that they needed to return to Mexico, and then I started working in the schools. In these were the years that all these Hispanics started arriving here that don't speak English, and that is my passion to help those people understand what resources are available to them. This week about 5 ladies came to my door, they knocked and bringing me [sic] problems in their lives/community. This week I am working in the schools and right now I am working at [local High School]. I serve there because I have a daughter that teaches there. However, I volunteer at various schools. We want to make it known to those that don't speak English what their rights and what resources [are] available to them. [A focus group participant] [Parekh 2018; U]
They reported that asking neighbours whom they did not know for help was less appealing, even during the pandemic. [Jagroep 2023; U]	Some interviewees did not have broad neighbourhood social networks during the pandemic, and contact with neighbours was an important means of overcoming the barrier to asking for help. The interviewees described the current pandemic situation as difficult and lonely, and stated that they expected neighbours to keep an eye on each other, especially on the older population, as they were all in the same boat.<br>"Also, in this situation[the COVID-19 pandemic], no one will come by to ask, 'How are you doing?'. And you are living in a residential community. I would like it if people would ask how I am doing or if they could do something for me." (Participant 11) (p. 6) [Jagroep 2023; U]
Older Surinamese adults appreciated this attention from familiar people in their neighbourhood, which made them feel like a part of the community. [Jagroep 2023; C]	During the pandemic they missed this attention as they had spent less time outsideduring the pandemic than previously. The participants indicated that greetings made them feel respected, and that one receives respect when one gives it to others." (p. 7) [Jagroep 2023; C]

“The interviewees reported that their main source of information about neighbourhood activities and events was other people. [Jagroep 2023; C]	They indicated that they received less information of this type during the pandemic, as they had less contact with other older adults whom they had previously met at neighbourhood activities (e.g. at community centres). (p. 9) [Jagroep 2023; C]
The men in this study talked about their engagement in religious activities organised by the mosque.(p.06) [Buffel 2017; U]	A 71-year-old man, said:<br>‘Because I’m active in the mosque association, I know many people. I have connections with almost everyone around here. We organise language courses, trips, and activities for young people. I meet my friends in the mosque, we go for tea after or we meet each other in our homes.’(p.06) [Buffel 2017; U]
Civic participation indicates individuals’ involvement in influencing decision making in their domains, individually or as a group, and whether or not they have taken action in an attempt to solve problems in their communities. The management issues in the ethnically homogeneous residence caused some older adults to take initiative to speak up and pursue collective well-being (p.24)… Such civic participatory efforts, however, were evident only among the elders who had experienced management problems in their building. The majority did not report any significant involvement in local, municipal, provincial, or national affairs.<br>(p.25) [Luo 2016; U]	They demonstrated strength and resourcefulness in civic participation and reaching out for aid, despite numerous difficulties, such as a lack of supportive resources and information at the beginning of their appealing process, and poor physical mobility of some members. As a female focus-group participant in her 60s said, “Therefore, we, this group of people, had made many efforts [to ensure everyone’s voice was heard] to elect the Tenant Club Executive Committee. . . . Because we cannot stand watching older elders being deprived of welfare and benefits. I could not stand [the injustice], so I stood up.”<br>Their allies included a local Chinese community leader, a local Member of the Legislative Assembly (MLA), the Elder Abuse Prevention Services at a senior centre, the Residential Tenancies Branch of the provincial government, and two lawyers.<br>(p.25) [Luo 2016; U]
Participants also described relationships with neighbours at the other end of the spectrum where they looked out for each other’s homes, had keys to each others’ houses, and called	**One man (78, African American) talked about keeping an eye out on a neighbor’s home when she leaves for a few days: “Well, like the lady across the street, she’s a widow, she’s about eighty-three. Whenever she goes**

regularly to check on each other (p.05) [Yen 2012; U]	**to see one of her daughters she'd let me know, "I'm going to be gone three days, four days." I watch the house to see if anybody's coming around or what have you.(p.05). Another participant (84, Caucasian woman) explained that a group of her neighbors have each others' house keys: "Interviewer: When any of your neighbors go away for trips or anything, do they ever ask you to look in on their place? Participant: Well, yeah.We do that for each other. The corner house, myself and the other house, we all have keys to each others' homes. They have my key, I have their keys. Which makes it nice. Helps." (p.05) [Yen 2012; U]**
Hence, through active processes of place-making, including building relationships with other ethnic groups, participants had overcome feelings of unfamiliarity and managed hostility to begin embedding in place. However, embedding, unlike the rather static, achieved notion of embeddedness, highlights dynamism and the need for continual effort over time.(p.05) [Ryan 2021; C]	Importantly, all participants had worked and lived in diverse environments and established relations with colleagues and neighbours from varied ethnic groups, including white English people, who became friends. Moreover, most participants had ethnic diversity in their extended families through intermarriage. Gabriel, originally from Jamaica explained: 'all my kids are mixed race … the country is mixed.'(p.05) [Ryan 2021; C]
A supportive social environment consists of the psychosocial domain ("knowing people," having a place to volunteer and connect), the environmental domain ("the bus is near," "temple is near"), and the cultural (a culturally familiar place of worship, where her first language, Punjabi, is spoken). (p.643)<br><br>They get out the door and engage with their local community because they have access to activities, shops, services, and social or religious gatherings that are culturally familiar.(p.644) [Tong 2020; U]	These few blocks, these are my village. Because I know those people. [The] bus is near. And my temple is near. [When] I'm not feeling good I go there. And on Sunday I go and volunteer there . ... When we bought this house, we thought the gurdwara (temple) should be near—every weekend we should go. (p.643) [Tong 2020; U]
Many respondents in England also took particular precautions when collecting their pension from the Post Office. A number of women reported that they would never take their purse with them when going outdoors	For example, some commented on their shared willingness with neighbours or other residents to intervene in local social control, keeping an eye out for risks to each other's homes:

and some deliberately dressed down, avoiding the wearing of jewellery or similar. A further strategy, however, was to develop collective responses to issues of community safety, embracing activities ranging from informal social control to collective actions supported by local organisations.(p.101) [Buffel 2013; U]	If I see a car out there and the car is there for a good while I will go out and say 'Who you come visiting for?' . . . Like my neighbour next door – he went away on holiday and I see two lads and I ask them. Maybe if I didn't ask them, maybe they break in. (60- year-old Black Caribbean woman, years in the neighbourhood, Longsight, Manchester) (p.101) [Buffel 2013; U]
Some older migrants also create 'places of belonging' by participating in residents committees, community centres and religious associations. In general, we found that older women's participation in such activities tended to be rather limited, especially among Somali people. However, some women had important roles as volunteers in self-help organisations and community centres.(p.102) [Buffel 2013; U]	An older Moroccan woman in Brussels, for example, said:<br>I go to the community centre every day. I help with cooking and I'm involved in organising activities so that we can do things together . . . it's important to mix with people from different cultural backgrounds. (-year-old Moroccan woman, years in the neighbourhood, Old-Molenbeek, Brussels)(p.102) [Buffel 2013; U]
Khmer cultural practices continue to dominate across most interactions within the village.…Khmer families, such as Prahm's, reunited or newly formed, have striven to create a feeling of home, security, and meaning in Veluvanna Village. (Khmer community dominant rural village in the US) (p384) [Lewis 2009; U]	The architecture of the Buddhist temple, located in the center of the village, is decidedly Khmer style. Most homes have gardens where traditional Cambodian fruits and vegetables are grown. One family has dug deep pits for growing lotus, a flower with significant meaning in Cambodian culture, and other traditional aquatic plants. Herbs from Cambodia sprout under canopies of massive green leafy vegeta-<br>bles, squash, banana trees, and tomatoes. Fragrant flowers, purple and spiked, share space with lemongrass, several kinds of mint, basil, and many different herbs. The yard is filled with plants Mai, Prahm, and others had eaten and used as medicine during the "Pol Pot time." Plants for food or for medicine grow rampantly thanks to the rich fertile soil, the blazing coastal heat, and the near-daily afternoon showers. Family, friends, and neighbors greet one another as they move about the village. Some bring vegetables to elderly women who provide care to multiple

	grandchildren while their mothers are working; some come to purchase duck eggs or deliver a bit of news, enjoy some fruit, and hear me speak Khmer—a source of great pleasure and amusement for many of the older women in the community. Prahm, one of the original creators of the community, explained the importance of the village as a cultural space. He said,” Here [Veluvanna Village] I can close my eyes at night and feel safe.” (p384) [Lewis 2009; U]
While these discussions of stability, co-ethnic neighbours and religion point to some differences in the experience of their local areas that Caribbean and Indian participants had, the sharpest contrasts between their accounts emerged when they were asked directly about the area in which they lived. As might be implied by the accounts above, Indian participants discussed the positives of their residential areas and the amenities they have access to, particularly in relation to the presence of temples, community centres and the fact that there were other residents of the same ethnicity.(p.555) [Becares 2013; U]	There are a lot of our people around here. No one fights, everyone is nice and friendly ... everyone is nice to me. They always say hello to me... Yes it is all right. It is safe. There are many of our people here so it feels safe... The Gurudwara [Sikh temple] is only 10 minutes and the temple is 15mins. I can walk there ... There are buses near the street. It is only a 5 minutes walk. The service is good. I take a bus to go to the city. (958, Indian man in his late 60s/early 70s). [Becares 2013; U]
Such account was not were not present in the Indian participants' interviews, where mentions of experience or fear of crime were coupled with characterisations of the local area as nice. [Becares 2013; U]	The area is very nice and the people are very nice. Our neighbours opposite who are white are very very nice people. If I call them at 12 o'clock at night they will come. The kids who Live opposition is very bad. They are youngsters and they are very bad children ... They are always creating problems. So now it is all right. They broke our glass in the window. (588, Indian woman in her early 60s) [Becares 2013; U]
For the participants, ageing in place is associated with emotional bonds and belonging to the neighbourhood. Although many participants reported that language was a barrier to full involvement, many established good relationships and emotional	Hong, a 75-year-old woman, told the interviewer that she and her husband provided instrumental assistance to their neighbours. This assistance included collecting letters and newspapers and watering gardens for holidaying neighbours. Hong also told the interviewer a story about how she and her

bonds with their neighbours through their engaging in helping practices.<br>(p.30).<br><br>Hong's account showed that through specific acts, people could become members of a place and come to trust that their needs would be met. This process came to the fore through supportive interactions among neighbours. Supportive social interactions led the members of the neighbourhood to attach meaning to the local setting and foster belonging and familiarity. As a result, the neighbourhood became a place where the person could gain membership and establish emotional connections foundational to the development of social capital and social cohesion. In the aforementioned example, Hong, her husband and their neighbour spoke different languages yet were able to support each other<br>(p.31)<br>…This finding suggests that the resettlement experience of migrants is more complex than is presented in migration research, which usually regards language as one of the sole key indicator of successful resettlement and ignores the roles of social interactions and means of communication that are beyond languages and can span cultures. In this sense, our participants engaged in unique practices, compared with other migrant groups, because they did not rely solely on spoken language to cultivate a sense of Community (SOC).(p 30-31) [Li 2014; U]	husband utilised their language skills to help a neighbour:<br>I learnt English when I was young. My husband studied in Russia for five years. One day, our Russian neighbour lost her wallet in a supermarket and came to us for help because she failed to communicate with the staff member in the supermarket. We went to the supermarket with her. She talked to my husband in Russian. My husband translated what she said into Chinese to me. I explained to the staff member in the supermarket in English. The lady got her wallet back. (Smiles.)<br>Hong was very proud of herself when she told the story. She told the interviewer that being able to help her neighbour made her feel that she was part of a larger group, rather than an isolated person. [Li 2014; U]
A cross-account examination suggested that satellite television provides not only older Chinese migrants an opportunity to virtually participate in their home community but also the local residents in New Zealand to participate in their Chinese neighbour's home community. (p.33) [Li 2014; U]	Ming's (a 76-year-old man) narrative embodied the personal, the political and the cultural threads that, when woven together, fostered Ming's SOC for both his home and host countries:<br>After a large earthquake struck Sichuan, China on 12th May 2008, we learnt from the Chinese TV news that tens of thousands of

	people died. We organised fundraising activities to support the victims of the earthquake. Our New Zealand neighbours showed great compassion and donated money to the earthquake survivors. We really appreciated that.<br>The participation of Ming's New Zealand neighbours in supporting Chinese people sustained Ming's multiple SOCs and enhanced his emotional ties to China as well as New Zealand. [Li 2014; U]
Hispanic and Vietnamese older adults discussed engaging in informal civic activities within their ethnic communities, such as advocating for residents with parking tickets, connecting at risk children and families with social services, and serving as public notaries in their community, members of these ethnic communities were often disconnected with mainstream society and relied on each other to meet their basic needs. The degree of reliance on the small community social group indicated that while decreased level of social cohesion within larger groups and communities affected civic participation, normative goals of social capital that help individuals create social identities foster civic participation in many non-formal ways within the social network.(p.501) [Parekh 2018; U]	A 72-year-old Hispanic male in our study shared how he gives back to his ethnic community:<br><br>Sometimes people in the community get a traffic ticket, so I help them with their needs, so I help them with the process with that or litigation. I am not an attorney but I know the process. That's how I give back to the community. I'm a notary public for the community but I don't charge them. People can make in-kind donations especially the immigrant community giving them guidance and support.<br>[A focus group participant] [Parekh 2018; U]
Older adults across various age, income, and socioeconomic status perceived that their place of congregation not only was a source of support but also provided them opportunities to be involved in their communities. Congregant memberships represented social connection with a larger group (i.e., social cohesion) that bolstered opportunities for civic engagement. As such, older Hispanic adult participants in our study who resided in low-income sections of the city believed that building a much-needed church would greatly help them develop a sense of community,	A 62-year-old participant reiterated:<br><br>We are trying to start a church here. The church is very important in this community and is the first place for the children.... so that the children can grow up in the church. ...in a Christian atmosphere and the elderly people of the church are helping us take care of children and teach and do different things.<br>[A focus group participant] [Parekh 2018; U]

provide resources based on this social network, enhance intergenerational relationships, and further motivate them to participate in various activities within the community (p.501-502). [Parekh 2018; U]	
women's narratives about social support appeared to be centred on relatives, sick or vulnerable people in the community, and the looking after, or being looked after, by neighbours.(p.06) [Buffel 2017; U]	'I am very happy with my neighbours. They helped me a lot when I was having a difficult time after my husband died. May God reward them' (74-year-old woman).<br>'They [neighbours] are very important to us… Even in the smallest of matters one neighbour can help another […] "Komsu komsuya muhtaçtır" [loosely translated as: It's better to have a good neighbour than a distant friend]' (64-year-old woman).(p.06) [Buffel 2017; U]
As reflected in these quotes about the role of neighbours, the interviews revealed a range of Turkish expressions about the value of 'neighbourliness', both among women as well as men. Some participants made this point by using the Turkish proverb 'find your neighbour, choose your house', suggesting that trustful and supportive neighbours are the most important criterion for determining the choice of a home. (p.06) [Buffel 2017; U]	Other expressions included: 'neighbours are like family in our culture' and 'neighbours have indisputable rights to each other'. Typical examples of such 'rights' were found in the reciprocal support, both emotional as well as practical, provided to one another, in terms of 'helping each other financially' when needed (71-year-old man) and 'visiting neighbours when they have a problem or when they are ill'(74-year-old woman).(p.06) [Buffel 2017; U]
Monolingual seniors found Chinatown attractive not only for convenience and autonomy but also for sociability and daily normalcy. This further required them to negotiate the space of everyday life in Chinatown. The grandiose Chinese arches might be important for tourist development or urban diplomacy with Shanghai, but They played a trivial role in Chinatown residents' life. In contrast, social and exercise space in Chinatown was important for them to cultivate a sense of belonging, social relations and individual wellbeing P.339.<br>In addition to public or semi-public areas, the respondents in this research also took	Despite the accelerated development for tourism, Montreal's Chinatown continues to provide a vibrant social life for seniors. A long waiting list of 4 years on average did not prevent monolingual seniors from applying for a subsidized one or two-bedroom apartment in one of the five Chinese-run housing complexes. Construction began during the 1980s to accommodate the rising amount of monolingual single senior dwellers. About 400 units reduced the number of seniors living in deplorable rooming conditions. Nonetheless, these seniors did not confine themselves to their apartments. It is common to see Chinese seniors using

advantage of common areas in subsidised housing complexes and Chinese organisations. They socialised, took language or exercise classes and participated in leisure activities such as Mahjong, Tai chi, choirs and Chinese opera, among others. P.340 [Hsu 2014; U]	hallways or food courts in nearby public buildings and shopping malls for socialisation. They gather together to chat, to play Chinese chess or simply to sit alone reading community newspapers. It is<br>equally common for them to spend a whole day in the YMCA in the Guy Favreau building. While some fitness classes are specifically designed and instructed in Chinese, Ping Pong and badminton courts are popular among older immigrants.<br><br>Mrs. Yip recounted her everyday life:<br>I can walk to every corner in Chinatown. Friends or relatives take me out of town for BBQ or to the countryside on the weekends…I go to the YMCA every day. Tuesday and Thursday I study [French] at the elderly club. I also stay there for extra two hours or so just to chat…I volunteer in the Chinese hospital, as I can chat with those who have nobody to talk to in Teochewnese (Interview, 13 Dec 2008).P.340<br>Chinatown was mapped out by Mrs. Yip according to sociability and peer support.Her narrative also singled out how comfort and a general sense of satisfaction were negotiated out of the paradox of autonomy and dependence. Even though she did not confine everyday life to the ethnic quarter, her autonomy from family and children nonetheless depended on ethnic functions in the small ethnic community, ranging from the convenience of grocery shopping, the ease of mobility, the availability of peer support, to meaningful habitual practices. [Hsu 2014; U]
Although social gerontologists have tended to focus on the social isolation and exclusion of older urban dwellers, increased attention must also be given, as Becker notes, "to the wealth and complexity of social relationships in the lives of many ethnic elders, for these relationships are key to understanding what keeps these elders rooted in place" . (p.22) [Buffel 2011; U]	This point is clearly expressed by an older Pakistani man living in Manchester who argued that moving away from his neighbourhood would be "the biggest mistake ever, because my wife, children and myself get a lot of support from the people in this area." [Buffel 2011; U]

Many interviewees also contributed to social capital through maintaining "good and trustful relationships with neighbours," these providing them with a sense of security. (p.23) [Buffel 2011; U]	A Pakistani respondent commented: "They [the neighbours] are there if you need them and you don't have to worry about anything. We always help each other out." Several Turkish people made a similar point by using the Turkish expression "find your neighbour, choose your house," suggesting that trustful and supportive neighbours are the most important criterion for determining the choice of a home [Buffel 2011; U]
For some, the importance attached to neighbours was also inspired by cultural views and Islamic beliefs. (p.23) [Buffel 2011; C]	This is illustrated through the following expressions: "neighbours are like family in our culture"; "Allah evaluates our efforts to maintain neighbourly relationships"; and "neighbours have indisputable rights to each other." Typical examples of such "rights" were found in the support provided to one another, in terms of "helping each other financially" when needed and "visiting neighbours when they have a problem or when they are ill." [Buffel 2011; C]
Finally, despite feelings of separation with the non-English-speaking community, participants described a sense of pride in their own communities that they thought Wisconsinites should be aware of: [Hawkins 2022; C]	We're hard workers, and very intelligent...We're humble people." – Participant 17 (80 years old)" (p. 118) [Hawkins 2022; C]
When social relationships were developed outside the ethnic community, they were often with culturally similar others in their communities, particularly those from Asian or Buddhist backgrounds. [Wijekoon 2018; U]	Ravi interacted with Indian passengers on the bus, Vijith made friends with Tamil Sri Lankans at the mall, and Kamal acquainted a man from Thailand during his walks. Interactions with persons with comparable values, beliefs and interests, who akin to themselves were 'outsiders', bred a sense of familiarity in the host country." (p. 166) [Wijekoon 2018; U]
Tight neighborhood connections with neighbors of the same ethnicity served as a safety net for older adults with no access to public transportation. [Dabelko-Schoeny 2021; U]	A Somali older adult emphasized: "I live alone,but my neighbors and the Somalis,they come to me. They ask me how I am, if I want anything, if I want to go anywhere". (p. 7) [Dabelko-Schoeny 2021; U]

As participants aged, they developed a range of relationships through which they gained support in their communities, facilitating interdependence and helping them develop resilience and a sense of inclusion. [Nasir 2022; U]	For example, Aya repeated several times a quote from the Quran, which helped her reach out to cultivate interdependence by forming strong social connections with her neighbors. She said "Prophet Muhammed, Peace Be Upon Him, said to be good and kind to your neighbors and you're more than a neighbor" and narrated how she and one of her neighbors had built a strong connection that had lasted for over two decades. Aya considered this neighbor as her own sister, explaining "She's the one who used to take me and sit with me [pause], not my own blood sister. She's the one. And my sister wasn't that far away from me [geographically]". She expressed that her life would be different if it did not include being socially connected and regularly engaging with her neighbors, for example, by exchanging cultural meals and recipes with them, and looking after each other's homes (e.g., collecting mail) when they were on vacation, which provided feelings of safety and social inclusion." (p. 9) [Nasir 2022; U]
Within these green spaces, older Chinese immigrants have the opportunity to contact neighbors regularly, such as by greeting, smiling, or recognizing faces. Close to three-quarters of the participants (77%) expressed that, through this regular contact with their neighbors, they gradually developed an attachment to the neighborhoods, which positively influenced their well-being, [Gao 2020; U]	as denoted by Dong (male, 67 years old, living in Australia for 4 years): "I go to the nearby park every day, and I can see some familiar faces. They look very friendly and warmhearted. They show me how to use fitness equipment; our dogs can play together. I feel very happy that I can live in that neighborhood. I feel I am a member of that place now." (p. 9) [Gao 2020; U]
What Samantha enjoyed most about her current living situation were the countless children from the neighbourhood who visited her house almost every day to play with her grandchildren. [Schuster 2019; U]	During those visits, Samantha usually watched the little ones and made sure that nobody got hurt or picked a fight: "[Laughs]. Sometimes it's a big mess. But I like them, because they can play together. [...] I am always with them or just taking a look. [...] It's good for me." Samantha said that not only did the children appreciate this kind of caring attitude towards them, but also their parents

	were happy about having such a thoughtful grandmother in their neighbourhood. Laughingly, she explained that everybody called her “abuela”, which means “grandmother” in Spanish: “And when they all call me like ‘abuela’, I am feeling like I am their ‘abuela’, too… (Schuster, 2019, p. 56) …“The Canadian neighbour told us one day: ‘Since you came here, the block is always full of kids playing, running. So, I think that you are [...] attracting all these kids.’” (Samantha)” (p. 78) [Schuster 2019; U]
Participants reported that they received practical and emotional support from their (grand)children and their neighbours…<br>So the neighbours have an essential role in keeping an eye on each other. Receiving support from neighbours and keeping an eye on each other contributed to participants being valued and part of society, increasing their social connectedness. [Jagroep 2023; U]	Whether (grand)children lived in the same neighbourhood (at walking distance) varied among participants; those who did offered more practical support, described as letter reading, the filling out of forms, grocery shopping, cooking and the collection of medication. Participants indicated that trusted people (e.g. their children), rather than others such as neighbours, read personal letters out loud and filled out forms related to their finances. One participant reported that her neighbour had a spare key in case of emergency. One participant reported that her neighbour rang the bell to see how she was doing and to have a chat when she had not seen her for a few days, which she liked very much. Another participant indicated that he kept an eye on a neighbour because she was in her nineties.<br>“I also have a Surinamese Javanese neighbour. If she has not seen me for a day, she will tap on my window,‘Oh neighbour, I have not seen you. I have missed you’. Then we have a little chat. She keeps an eye on me. I like that she still does it.” (Participant 9) (p. 5) [Jagroep 2023; U]
The interviewees reported receiving different degrees of emotional support from their neighbours during the pandemic… [Jagroep 2023; C]	Some older Surinamese adults indicated that their neighbours asked them how they were doing more often than before the pandemic, whereas others indicated that they expected more emotional support from their

	neighbours. Participants received support from neighbours with whom they had regular contact before the pandemic, a majority of the participants indicated that these neighbours also had a Surinamese background. They indicated that support from neighbours is essential when one is vulnerable, such as during the pandemic." (p.6) [Jagroep 2023; C]
The interviewees' reported social participation in the neighbourhood varied, and included activities such as meeting for coffee, playing bingo, practicing yoga and being busy in an allotment garden. [Jagroep 2023; C]	The interviewees indicated that these activities provided opportunities to be physically active and socialise with other people and functioned as a platform for the meeting of new people and making of friends. They also stated that such activities provided opportunities to remain engaged with and informed about their neighbourhoods.Older Surinamese adults reported that it was their own responsibility to maintain inclusion in the community by going to these neighbourhood activities, as it had a positive impact on their well-being. Most of the activities mentioned took place in interviewees' own neighbourhoods." (p. 6) [Jagroep 2023; C]
Although participants could not communicate well with some of their neighbours who do not speak Dutchwell, they felt it was important to share cultural practices with them, by sharing food. [Jagroep 2023; C]	They stated that such experiences contributed to their feelings of connectedness and inclusion in the community." (p. 7) [Jagroep 2023; C]
Some study participants reported that they communicated with their neighbours through Whatsapp,<br>… However, not all study participants had smartphones enabling neighbourhood app use, suggesting that they would be excluded from relevant information about their neighbour hoods. (p. 9) [Jagroep 2023; U]	for example, to inform each other about neighbourhood activities, to alert neighbours of a bicycle in the way or to report noise nuisance: We also have a groupapp with neighbours in it. If there is anything or when we want to give information about occasions in the neighbourhood, we forward it to each other. (Participant 7) Participants appreciated this way of communication because wider groups of people could be reached. (p. 9) [Jagroep 2023; U]

Another factor which had an influence on Samantha's ability to participate in activities was her health. In order to minimize this limitation, Samantha made sure she contributed to her health by eating healthy and exercising regularly. However, she explained that she missed her church which she needed in order to retain her health: [Schuster 2019; U]	"I need to go to my church. But now, I cannot go because it's very far. Catholic churches are not near [...]. I cannot change my religion. I cannot do anything about that." I noticed that Samantha compared her life in the old neighbourhood with her life in the new neighbourhood. She expressed her sadness about having left her church and her friends from the south behind." (p. 58) [Schuster 2019; U]
The interviewees emphasised the value of community centres, which offer neighbourhood activities and a place where older adults can volunteer. [Jagroep 2023; C]	Many interviewees reported that they volunteered to keep themselves busy and in touch with others… "They described the community centres where they volunteered as open to everyone and as places where people could meet, learn, and socialise,which contributed to their well-being and their feeling of being valued. (p. 10) [Jagroep 2023; C]

3.5 Long time tenure in current neighbourhoods

Author Statement	Illustration
when asked about the experience of aging in place, a majority of participants connected the concept to the broader community where they resided and had daily activities instead of the concept of a house or home. Some immigrants have been living within the Chinatown for a decade. (p.383) [Chen 2022; C]	I have been living in Chinatown for 13 years. Although I have moved several times, it's always in the same area. (P4, Male, 70) (p.383) [Chen 2022; C]
Participants reported little interest in moving outside and had a vague impression on the outside areas and other Chinese enclaves (p.383) [Chen 2022; U]	I don't know much about other Chinatowns in the city and have no interest in going outside. It is great for people living in 8th Ave because we know the language and can buy things within walkable distance. (P20, Female, 67) (p.383) [Chen 2022; U]
People would often express strong emotional feelings about their neighbourhood. Even those who held the strongest views about the 'loss of community' still reported a	I don't fancy another area for living. (-year-old Black Caribbean man, years in the neighbourhood, Moss Side, Manchester)(p.97) [Buffel 2013; C]

close identification with their neighbourhood. Many residents expressed their attachment though a reluctance to move to a new location. (P.97) [Buffel 2013; C]	
Indian participants talked about the importance of the stability of the population in their local area and of knowing other local residents with similar ethnic backgrounds. A central and repeated element of these narratives was about the positives of having known neighbours for a long time, because both they and their neighbours had lived in their houses for a long time, and this was contrasted in some accounts with the negatives associated with migration into and out of the area and of changing communities.(p.553) [Becares 2013; U]	**Here we have many close friends irrespective of their caste. Both our natures are such that<br>We have many close friends. There has never been a day when I have not had visitors. One<br>leaves and the other one comes in. It's like that all day. So we don't feel lonely. Neighbours come or someone else comes, some friends come to visit ... We have been living here for 23 years and our neighbour next door has been here for 10 years, They are Baniyas and the Katchis opposite arrived earlier than them. They have all been here for some years ... They all know us. (601, Indian woman in her late 60s/early 70s). [Becares 2013; U]**
The meaning of home; she has lived in the reasonably priced three-bedroom apartment for 25 years. To Ann, it's home and forms a significant part of her identity [Brotman 2017; U]	Ann lives on a low income, and people have been trying to persuade her to move into an HLM, but Ann is resistant. She has lived in the reasonably priced three-bedroom apartment for 25 years. To Ann, it's home and forms a significant part of her identity: "I don't say I moving out from [my neighbourhood], trust me, even somebody give me a free house somewhere (everyone laughing). I don't want to go. You know everybody say like 'get a low income' [apartment], I don't want a low income, I don't want to live poor. I want to be me." Ann points out that in an HLM, she would only be eligible for a one bedroom, because she lives alone, but that would not give her room to host her three children and several grandchildren when they come to visit. She would also have to get rid of many of her things that provide continuity of identity: "part of me is in this stuff you know? It's like, I tell them when I die, whatever. But until

	then ...my mother have lots of things. And my mother never get rid of nothing." She also speaks to how she likes the neighbourhood: it's not too quiet, like the neighbourhoods she's seen with HLMs, and she feels safe walking there, even late at night. She has the sense that everybody knows her, and friends of her children recognize her and tell her, "I come up at your house and you cook the goods that I like." She is a part of the community and the community is part of her (p. 66) [Brotman 2017; U]
Participants described not only their connections to their neighbors, but also within their neighborhoods more generally. [Nasir 2022; U]	Ali had lived in his current home for nearly a decade and talked about how much he liked the nearby amenities, making it was easy for him to drive to a café every morning where he met his friends to socialize about the community and politics. Ali said, "It's a nice quiet neighborhood. Everything is nearby, Tim Hortons, restaurants, Food Basics, shoppers drug mart, you have a lot of services". The café was a meaningful place for Ali, where he could enact important social activities with his friends and relatives, which promoted his level of social engagement. He stated, "I'm retired now I don't do anything . . . , except having coffee and playing cards. Having coffee everyday with friends"." (p.9) [Nasir 2022; U]
The study participants indicated that they had lived in their current neighbourhoods for decades and knew their neighbourhoods well. [Jagroep 2023; U]	They reported sharing joys and sorrows with neighbours whom they had known for decades, which contributed to a sense of belonging to the community. They stated that they knew the local people and stores and recognised familiar faces, which they perceived as inviting greetings and chatting (e.g. with shopkeepers): You go to shops where the employees know who you are and know what you come for. We always have a chat. At home I am alone. So when you go outside, you can chat with someone whom you see regularly. You do not have to tell everything, but you can have a chat. (Participant 15) (p. 7) [Jagroep 2023; U]

3.6 Changing Neighbourhood Composition

Author Statement	Illustration
Twenty of the 38 participants had lived in their neighborhoods for over twenty years. These people often observed that there had been a lot of change in the composition of the neighborhood population and that they used to know more of their neighbors. A common experience was that the participant would recall that when their children were young, they knew neighbors who also had school-aged children. Over time, households would relocate as children moved out. The newer neighbors might be working aged, away during the day, busy with their own young children, and less available for intermittent neighborhood socializing. Sometimes, the lack of familiarity caused uncertainty or insecurity. People's discomfort was frequently a result of perceived social distance from the neighbors, being far apart in age or of a different ethnicity. [Yen 2012; U]	A man (70, Latino) commented on young people hanging around in the neighborhood, giving him a feeling of insecurity: "In the outskirts of our neighborhood there's been more kind of young kids congregating on corners. On the business district there's more young Black and Latino kids, maybe sometimes a White kid, too, but Black and Latino kids, kind of acting rowdy, loud." Later, in response to a question of whether there was anything he did not like about his neighborhood, he added: "What I don't like is the sense that it's become a little more dangerous, you know, in terms of reading about assaults, and seeing kids acting out, you know, on the street. You know, fifteen year olds, acting crazy".(p.05) [Yen 2012; U]
People who were once newcomers became locals, observing their neighborhood changing its familiar ethnic identity. This common experience, however, prompted different reactions. (p.838) [Lorinc 2022; U]	**"When I moved in, there were lots of Afro-Caribbean people in the road and lots of White people, and they moved out and Asian people have moved in ... The whole area has changed in the last 20 years," Marjorie described. Millicent highlighted that "all nationality eats different," and with increasing Eastern European arrivals, food shops in her neighborhood started catering for them: "when you go in looking for something that you're accustomed to, sometimes they don't have it…<br>While Marjorie felt isolated and lonely in her now predominantly South-Asian neighborhood, Weldon, living in a place with similar ethnic dynamics, said: "We do get along very well ... I have good neighbors. They don't speak good English but we can**

	**still have a good conversation." (p.838-839) [Lorinc 2022; U]**
As Hickman and Mai (2015) observed, changing demographics can be a factor in how older people perceive neighbourhood. Long term residents can begin to feel like strangers as neighbourhoods change around them… Furthermore, as May and Muir (2015) highlighted, it is all too easy to simplify older residents' sense of belonging in place through a lens of 'race' and racism. Older residents, including migrants of any ethnicity, may begin to feel displaced from their local neighbourhood when the demography of that place changes. (p.07) [Ryan 2021; U]	Originally from Jamaica, she lived in a Suburban area of London and owned a house with a nice garden on a quiet street. Marjorie recounted, however, that the neighbourhood had changed considerably in recent years and she felt that her ethnicity had become a marker of difference so that she no longer fitted in the area. As we observed when we walked around with her, the area had a large South Asian population, reflected in the local shops including clothing stores and food markets… [Ryan 2021; U]
As noted earlier, places can be imbued with markers of ethnic identity. Particular neighbourhoods can be associated with ethnic clubs, shops, pubs, and places of worship that underline a sense of home, belonging and local attachments, especially for migrants. [Ryan 2021; U]	Like Marjorie, several participants remarked on processes of transformation that changed the ethnic identity of some neighbourhoods. Barry, a 92-year-old Irishman in London, noted how Cricklewood was changing: 'there aren't as many Irish around here as there used to be. The older generation now are all passing away and their children move off and move out. They don't stay.'<br>Nowhere was this transformation more pronounced than in Kilburn, North London, an area with long historical associations to the Irish community (Hickman & Mai, 2015), which has changed beyond recognition. As it becomes more diverse, Irish shops, pubs, and cultural associations are no longer prevalent. For some older Irish migrants, this led to a sense of disembedding. Cathleen, a 90-year-old widow, lived in Kilburn since 1942 when she arrived aged 14 from Ireland. Although she had lived in the area for over 70 years, it now felt different mainly because her old networks were no longer there: 'Kilburn has … it's not Kilburn anymore … Well, everybody was very friendly years ago … My sister lived here and my other sister lived in the East End … they're all gone.' Cathleen lived alone in a tower block and felt no connection to her neighbours and no sense of community anymore, highlighted by a recent incident. During the weekend prior to our

	interview, the lift in her tower block was out of order:<br>"I sat outside for nearly a half hour last Saturday with the trolley … to see if I could see someone to take me up to the fourth floor and nobody came … I asked one, a young boy, I said, 'are you going in my lift?' And he said no. And that was it … there's nobody to help you here." (p.07) [Ryan 2021; U]
Some of the Caribbean participants responded in similar ways, positively mentioning things like access to shops, transport, community centres and churches, although their accounts did not contain the same positives around living where there were other residents of the same ethnicity (P.555). [Becares 2013; U]	I: Is the church that you go to local to here as well?<br>R: Yes. Yes.<br>I: And are there like a lot of shops and stuff around for you?<br>R: These shops round here is not for really West Indies people. Them more cater for them own.<br>I: The Asian community?<br>R: Yes. Yes. But if you want fruit you can get a lot of fruits but if you aren't full provision not one here what them sell is halal chicken, halal [pause] sheep meat or something like that.<br>I: Halal meat isn't it?<br>R: Which I don't think is not for you really but if it's fruit you can get an awful lot of fruit.<br>(765, Caribbean woman in her late 60s/early 70s) [Becares 2013; U]
Older Surinamese adults reported that the compositions of their neighbourhood had changed over the years, with the loss of neighbours due, for example, to relocation or death. They stated that connecting with new neighbours was not always easy. .. [Jagroep 2023; U]	For example, some interviewees indicated that their neighbourhoods now contained many students or young families, who are often busy. They stated that they would appreciate new neighbours' coming to introduce themselves because they feel it is important to know who lives where: I am not very active in the neighbourhood; however, it is important to know your neighbours. Knowing you belong to the neighbourhood. When people see me walking on the street or see me standing at the bus stop, then they know 'Oh yes, that lady lives nearby'. (Participant 12)" (p. 7) [Jagroep 2023; U]

Thus, the walking interviews revealed the erosion of serv- ices and public places (p.838) [Lorinc 2022; U]	Weldon took us on a “tour” of his neighborhood, showing us the sites of pubs and clubs he used to visit. Most of them were gone, along with the local post office, shops, and cafes: “You see, everything is closed here. If I want something, I go to town.” A nearby park was also left to deteriorate: Once upon a time ... there was a lot of flowers, beautiful flowers, well-manicured grass. It was beautiful, now it’s a wreck ... They got rid of the park-keeper and it gradually ran down ... This here used to be the park-shop and that over there the park-keepers’ office, and it’s all now closed and boarded up. [Lorinc 2022; U]
As a long- term resident in the neighborhood, Lohendra recounted how the area had transformed through successive waves of migrants and his changing relationship with other ethnic groups. (p.838) [Lorinc 2022; U]	Lohendra (82, London), a Hindu man with Indian back- ground from the Caribbean, also pointed out places that previously were pubs, betting shops, and bank branches, giving an account of his past activities and daily life: “You see that building there [now Islamic community centre] ... it was actually my local pub ... a big change.” When he moved to his neighborhood decades ago, his “White” neighbor “wasn’t that happy,” and Lohendra attributed this animosity to him being a “foreigner.” However, “in the end we became the best of friends ... when they get to know you ... nobody seems to have any problems with anybody,” he explained. (p.838) [Lorinc 2022; U]
As Buffel and Phillipson (2019) note, neighbourhood change, such as gentrification, may undermine place attachment for long term residents, especially older people, but may also introduce some benefits such as improved services. (p.07) [Ryan 2021; U]	Maeve and Matthew, an Irish couple, had lived in the same street in London for 50 years. Whereas in the past the neighbours owned their homes, now houses were mostly rented out in flats:‘The people changed the place because the majority of the people in our street, in most of the streets around, are not responsible for the houses … Because they're rented … It makes a big difference’ (Matthew). As a result, Maeve and Matthew felt their street was less friendly and less neighbourly.(p.07)<br>Elwira's neighbourhood also changed but for different reasons. Originally from Poland, Elwira and her husband, Jakub, owned a house in a quiet street in London. However, during

	the walking interview, she indicated how the area had transformed dramatically in recent years. This transformation involved both socio-economic and generational shifts as the neighbourhood had attracted a large inflow of young, affluent families. As we walked around, we observed the number of amenities targeting young families. As an elderly couple, Elwira and Jakub felt there was little in the neighbourhood to meet their needs. Other participants felt more positively about their changing neighbourhoods. For example, Aine, an Irish woman in Yorkshire, appreciated the influx of young families because local services and shops could be sustained as a result… [Ryan 2021; U]
The Turkish community had become more visible with the building of mosques, the establishment of ethnic and religious associations, the celebration of religious holidays, and the opening of ethnic businesses. This community is often referred to as ‘Little Turkey’ as it has formed a self-sufficient enclave, where Turkish restaurants, halal butchers, teahouses, groceries, jewellers, and bookshops serve their predominantly Turkish clientele. Access to such ‘third places’ where ‘imagined communities’ can be sustained<br>was considered to be of major importance by many…(p.07) [Buffel 2017; U]	‘Everything we need is close by: the mosque; the hospital; the pharmacy; Turkish markets; halal butchers… My children live nearby and I have Turkish neighbours. For me, a good neighbourhood is one with good neighbours and good streets where you feel safe… What else do we need? (64-year-old man)(p.07)<br>‘We found peace here…I got to know my nextdoor neighbours well…people are warm, there is a good atmosphere and there are plenty of Turkish markets and shops… I can walk to the grocery round the corner to get fresh vegetables… And we also have a mosque nearby’<br>(63-year-old woman).<br>‘I love this neighbourhood. It feels a bit like Turkey. It’s like “back home”. We have a mosque; there are many Turkish shops where we can buy cheap products; there are Turkish restaurants, and we know all the owners’ (63-year-old man).<br>(p.07) [Buffel 2017; U]
Puerto Rican and other Latina and Latino older adults in subsidized housing may have some of the most secure housing in a gentrifying neighborhood, but they	For Ana, whose mortgage is paid and property taxes frozen, and for Evelyn, as a resident in low-income subsidized housing for seniors, there is less anxiety about their own physical displacement in the storm of development

nonetheless feel insecure about their ability to make use of the local amenities that have been created to exclusively attract a younger, more able-bodied, racial, and class demographic. (p.52) [Rua 2017; C]	happening in Logan Square. But fear of socio-spatial displacement looms large, such as the disorientation older residents feel as the built environments that have marked their way-finding strategies and practices vanish. As the city transforms around them, with little imprint left of their former dwellings or social spaces, it is obvious that city life, politics, and policies have not been kind or concerned with the needs and desires of an aging population. [Rua 2017; C]
While some Puerto Rican older adults express doubt and displeasure about the future of the neighborhood, none are anathema to change, especially if it stymies the physical deterioration of the built environment and enriches the amenities and resources in the community. [Rua 2017; U]	"Ay sí, me encantan los cambios que están haciendo. Me fascina. Pasar por allí, decir, ay que bello pusieron este sitio." ["Oh yes, I love the changes that they're making. I'm fascinated. To walk over there [and] say, 'oh how beautiful they've made this place.'"] So spoke Cuca Ruiz, another Reverend Alvarez resident, when asked about her neighborhood. [Rua 2017; U]
Similar to many of her Reverend Alvarez neighbors, Cuca has lived in a series of under-resourced and underserved neighborhoods with few amenities. For this reason, signs of investment in the community and the convenient services proposed as part of new development projects are valued and appreciated by some of these Puerto Rican older adults, even though they themselves may not frequent these establishments due to comfort or cost. [Rua 2017; U]	"For those kinds of places, I'm very shy" Cuca confessed. "I like more humble spots." One such "humble spot" was the Discount MegaMall. [Rua 2017; U]
Once a bustling indoor flea market, the MegaMall was where many of the Reverend Alvarez residents spent their afternoons bargain shopping, eating, and socializing. [Rua 2017; U]	"There was a time [you could say] that I almost lived there," Cuca said. She would go to the MegaMall after work with friends or take her grandchildren to eat and walk around to browse the toys and other merchandise. Visits were less frequent after the fire that reduced the vending space to less than a third of its original size, and she noticed fewer and fewer customers and "window-shoppers." Troubled by the state of disrepair and neglect, she joined the ranks of former MegaMall frequenters. Despite numerous building code

	violations, a yearlong shutdown, an attempted eminent domain seizure by the city, and the fire, the MegaMall had been a fixture in Logan Square for more than twenty years. In the recent past, there had been talk of converting the site into an upscale commercial center, a grocery store, a year-round farmers market, or a cultural center, but none of these proposals gained traction. [Rua 2017; U]
The future of the MegaMall site was a regular topic of conversation at Reverend Alvarez when I conducted fieldwork. Residents sat in the community room or on the benches of the main entrance, with partial view of the MegaMall through large glass windows, discussing rumors of what would replace it, as well as what they would like to see as part of the new development. Cuca is in favor of a frutera (a basic grocery store that carries Puerto Rican/Caribbean and Mexican fruits, vegetables, and other imported food items) and a beauty salon. Others made their opinions known during the February election season when a candidate for the local ward seat held a meet and greet in the community room. [Rua 2017; U]	Two or three of the Puerto Rican residents present inquired about the redevelopment of the Discount MegaMall site: Was it true that a grocery store would replace the MegaMall? Will a medical facility be part of the new development, as some had heard? All in the community room approved of such developments. The candidate seemed puzzled and unprepared to address questions about new development and neighborhood amenities that would take into account aging residents. She explained that even though the MegaMall is across the street from their building, it is outside of the ward for which she was campaigning. Unsatisfied with the response, residents respectfully told the candidate that she should tell whomever is making those decisions that they want a grocery store and a doctor's office. [Rua 2017; U]
The arrival of bright green flyers announcing a PUBLIC MEETING about a proposal to redevelop the MegaMall site a few weeks later prompted a conversation about displacement. The Logan Square Neighborhood Association created and distributed the bilingual notice to inform residents of an upcoming community meeting organized by the ward alderman to discuss a zoning change requested by the new owners of the MegaMall site. [Rua 2017; U]	"the neighborhood is changing, becoming too expensive"; "the rents are going up, all the new people are white." In agreement with her neighbor's objections, Pura Santiago, a tiny, fair-complexioned Puerto Rican with a gruff voice, who most mornings joined Septimo on the main entryway bench to people-watch, added, "Puerto Ricans are tired of being pushed out and pushed around." "Los blancos se están quedando con todo esto" [The whites are staying with all of this]. [Rua 2017; U]
Reverend Alvarez residents murmuring	"It gave me such sorrow and pain to know that none of that is for us," Pura told me. "I

among themselves that they approved of the grocery store and wondered if they would be able to use the proposed gym. They were also curious about how many of the apartments would be set aside as affordable,<br>and if any would be designated for seniors. As the presentation [by the architect] progressed, however, the realization set in<br>that they absolutely were not an imagined demographic of this new development [for MegaMall]. Evidently, Pura had heard enough. After the architect's presentation, she announced to her Reverend Alvarez neighbors (and those seated around them) that she was leaving and walked out with another resident. (p.55)<br>…<br>In attending the meeting at the MegaMall, Reverend Alvarez residents re-entered a place that they once considered a welcoming and vibrant social space, a sense of place lost because of neglect and disinvestment. Conversely, reinvestment and redevelopment will likely ensure that they will not experience it as a renewed space of social engagement. As a result, older Puerto Ricans, such Pura, express feelings of dispossession precipitated by a lived history of serial displacement. Others, like Cuca, appreciate the investment in the neighborhood built environment, even though she does not feel that new investments are developed for her or her neighbors use. Though the proposed Logan Crossings project, as a new-build development, will not directly displace low-income and long term residents, Reverend Alvarez residents, by and large, feel that they have become as expendable as the aged structures they once inhabited and frequented. (p.56) [Rua 2017; U]	had to get out of there." "Puerto Ricans" she reminded "are always pushed out." Unlike Pura, Evelyn Santos had harbored no illusions that the new development would be for people like them. "It's for the yuppies," she said a few days later when the redevelopment of the MegaMall came up once again. Although her mobility is limited due to severe rheumatoid arthritis, which prevented her from attending the meeting, Evelyn keeps herself up to date on local debates and considers herself an engaged community member. She was adamant that she would "not set foot" in the proposed grocery store. "I have my store," she snapped. "It's a general store open to everybody."<br>Accordingly, Evelyn is committed to shopping in a place where she feels comfortable, where "la gente" [the common people] buy their food. [Rua 2017; U]
Many participants had strong local networks, built up over many years in particular neighbourhoods, including extended family networks that provided practical and emotional support… However, although many had	Phyllis (86), originally from Barbados, had a large family living in London. This proximity of family was crucial in creating a sense of home: 'London is home. With the children being born here, the grandchildren, the great

extended families living close-by, most participants lived alone or with a partner… (p.06) [Ryan 2021; U]	grandchildren… it is home. Yes, the family makes it home'.(p.06)…Nonetheless, the proximity of family did not necessarily mitigate loneliness. Phyllis lived alone. Her daughters worked full time and had their own families to care for. Although they contacted her regularly and visited at weekends, Phyllis spent most of her days alone and identified her biggest challenge as loneliness: there was one time there was a lot of friends coming in and out, and I would go and visit them. But now it's not the same. As you get older, your friends they die out or they, like myself, are all too old to travel. So, that's part of getting older, I suppose. [Ryan 2021; U]
the social inequalities faced by our participants in their daily lives (p.838) [Lorinc 2022; C]	Pointing to a newly opened expensive-looking cafe, Geraldine noted that "people like me don't use it." (p.838) [Lorinc 2022; C]
Asian respondents were less forthcoming about the topic of social distance though they commented about ethnic composition of their neighborhoods. An 80-year-old Asian man described that the proportions of Chinese neighbors changed over the 50 years he lived in the neighborhood. During some periods, there were more and during other periods, there were less. (p.06) [Yen 2012; U]	A 74-year-old Asian man said it made no difference to him if his neighbors were Asian or not. Yet, he was clear that some of his closer neighbors were Chinese, while he himself was Filipino. He did say that he liked to go places to engage with Filipinos, "I will always try to go to the place where there are so many Filipinos. To me, it's enough. But when I see Filipinos, I talk to them."(p.06)

Experiences over their life course and migration history influence their feelings of belonging in their current neighbourhood. Beyond attachment to where they are living, their sense of home is determined by family circumstances and support, and engagement with community spaces

4.1 Home is Family:

Author Statement	Illustration
Most participants referred to "home" as being with family. Most participants did not spontaneously use the term house (casa) or home (hogar) in referring to their sense of place, and this was true when talking about their early life in their native country as well as their later life in the USA. When asked, however, what the concept of home meant to them, the majority responded that home is family.(p.05) [Curtin 2017; U]	For Mauricio "…my home is my wife and children… home is where love is and the union of the family…" Sofia talked about home as "… the house where my husband and children live… where we can raise our children with love, affection and understanding of one another." (p.05) Tomás stated that "For me home is not the country, it is where I have formed my family and where my children have been raised and they learned culture, values and principles." (p.05) [Curtin 2017; U]
…other residential strategies have arisen because of circumstances surrounding life in the United States. There is a shift from multigenerational housing to clustering in neighborhoods or purchasing adjacent houses…Although the climate in the United States is too mild for the plants to make fruit, they provide a visual reminder of the landscape of Cambodia and a reduction in feelings of aging out of place. Her feelings of comfort, connection, and satisfaction occurred once she and her family devised a way to create an environment that felt more familiar, more like Cambodia and has allowed the entire extended family to insulate themselves through an ecological system of physical and socioemotional space. (p388) [Lewis 2009; U]	One elderly couple described how first their oldest son had purchased a house, then a son-in-law purchased the adjacent house, next a niece and her family bought a house behind the rest. After a few years, the entire extended family could travel from house to house without leaving the common yard. The wife explained, "We come here [to the United States] with so little . . . one house, then another house . . . we build our own village. My grandchildren . . . I hear playing and I feel comfort." She and her family also have planted banana plants along the fence that surrounds their family compound. (p388) [Lewis 2009; U]

Most participants, however, had adjusted to the idea of growing old in their second homeland. Their wish to 'return home' had become increasingly unlikely because of a combination of emotional and practical reasons. (p.05) [Buffel 2017; U]	'I can't move away from my neighbourhood. My roots are here now because my children live here… Hospitals are good, and we have insurance and social rights. Brussels has a lot of advantages' (74-year-old woman) (p.05)<br><br>'I would definitely prefer to grow old in my country [Turkey]… But I don't think I would be able to manage that. My children live here. I go back to Turkey for 3 to 4 months a year, but then I miss my grandchildren too much. I also don't have as many friends in Turkey as I have here. People are different there… Sometimes I feel like a stranger in my own country'<br>(63-year-old man). [Buffel 2017; U]
Findings suggest that the social network of most of the respondents was strongly oriented towards the (extended) family. The majority had their children and grandchildren living in fairly close proximity to their homes and had a regular and intense level of family contact. societies were not only important for the older person, in the sense that they lower the risk of being socially isolated, but also reflected the social roles older migrants take on in their families. (p.23) [Buffel 2011; U]	Typical examples were: providing support based on common experience; keeping the family together (especially among women); taking care of grandchildren; taking on an advisory role for the young; and promoting their culture and religion<br>I'm always busy in the home babysitting my grandchildren (Pakistani woman, Manchester).<br>When someone of the family is in trouble, they always come to me. They ask for my advice because I'm the eldest (Turkish man, Brussels). [Buffel 2011; U]
These two participants, who were surrounded by people via their daily interactions at a senior center, still reported feeling isolated, primarily due to separation from their families. While this finding was not reflected among the rest of participants, it is notable because it suggests further nuance to the relationship between social support and wellbeing." (p. 115) [Hawkins 2022; C]	Photo nine was taken by participant 24. She selected this picture because it was the beginning of her memories, "from this moment on I remember myself" – Participant 24 (68 years old) The picture was significant for participant 24 as it reminded her of memories of herself and her family during that time. Participant 21 (80 years old) summarized: "My family, I love them so much." (Mi familia, la amo tanto) [Hawkins 2022; C]
Feelings of mutual interdependence did not only stem from teaching grandchildren	For instance, **Ali expressed how much he enjoyed spending leisure time with his**

important aspects of the Muslim Lebanese culture, but also involved engaging in leisure activities and having fun together. [Nasir 2022; U]	**grandchildren, playing with them in the backyard and going on daily walks with them around the neighborhood, which kept him "involved seeing how they are doing". Ali had several grandchildren under the age of five and he explained how these connections filled him with gratitude because he was still physically able to play and build social bonds with them. Ali said, "Yes actually this is the most important thing in life, your children. You feel happy when you did what you are supposed to do. Like money or anything, I don't care about that, it can be reachable. But I care about their ethics and that we have raised decent people. So, I am happy for that, I think that is the main reason why I am quite satisfied". He also enjoyed helping his sons with their businesses by giving them advice when they need it." (p. 8) [Nasir 2022; U]**
Like Ali, Nabila moved to London in her old age to be among her relatives. (p. 9)	She said, "The people who stay happy live among those from their village. You reminisce about the past together, the children of your country, you live together, weddings you are dancing together, you are happy. We play cards, we play with everything, and we are all happy is what I mean. See the child of my country, my village, we were raised together and drank from the same water, and we walked on the same road". Despite being among relatives, Nabila mentioned that she has yet to build relationships with her neighbors." (p. 10) [Nasir 2022; U]
Most of them expressed a general sense of discouragement with regard to their neighbourhood, and some reported feelings of depression and loneliness. These narratives generally reflected a discrepancy between desired and actually achieved intimate social relationships (Scharf and De Jong Gierveld): (p.25) [Buffel 2011; U]	I feel alone; I don't have anyone to fall back on (Pakistani woman, Manchester). The fact that I have no family here is extremely bad and depressing; no good things come to my mind (Pakistani woman, Manchester). [Buffel 2011; U]

4.2 Gender shapes participation

Author Statement	Illustration
Ethnic Differences in Living Arrangements Affect Activities and Activity Locations. Latina participants tended to live with other family members, in particular adult children, more than did the White, African American, or Asian participants. Four of the Latinas lived with adult children. In two of these instances, their primary activity was to look after grandchildren during the work week.(p.06) Living arrangements somewhat affected use of services,in combination with an individual's gender and type of neighborhood (i.e., whether there were retail services close by and/or easy access to public transport). Living in close proximity to family was important for all participants. (P.06) [Yen 2012; U]	One African American man (age 66) lived with two of his grandsons who would drive him to places, because he had chronic health problems and some difficulty walking. An Asian couple (both aged 74-years) had retired from work in the Philippines and were living with an adult son. Apart from these four people, all the other African American, White, and Asian participants lived alone (n = 14) or with spouses. Similar to the Latina women, one of the Asian women (age 81) had been living with one of her sons looking after her grandchildren (e.g., taking them to school in the morning, picking them up in the afternoon, and cooking dinner for the family). As the grandchildren grew up and became involved in more activities, she then moved into anapartment in a nearby city that had been purchased for her by her children. (P.06)<br>An 85-year-old Latina woman was an avid gardener; her son would help her carry large bags of soil or mulch.(P.06)<br>A Latina woman (age 66) who lived with her daughter and the daughter's family might accompany her daughter to the store; however, most of her activity day during the week was at the senior center near her daughter's work and her granddaughter's school.(P.06) [Yen 2012; U]
Limitations, such as an inability to drive, has caused a shift toward elders being homebound, engaging in temple participation solely on weekends, and infrequent trips to visit family, friends, or markets because of the need to rely on younger family members who often are working….As her narrative shows, it is more difficult for elders to achieve the level of freedom they expect. Elders, such as the woman described above, limit their interactions with non-Khmers because of	One older woman, living in a small house with her daughter while caring for her young grandson, described how she had lost her freedom when she arrived in the United States. No longer able to walk to the temple, to markets, or to visit other elderly women, she feels trapped. She explained, "I am afraid to walk around my neighborhood. I don't know the people; they only speak English. They watch me when I walk so I am afraid." She stays inside her small frame home and

language difficulties and what they perceive as suspicious looks from neighbors. The critical aspect of neighborliness and belonging (Casarett, 1991) is missing.<br>(p387) [Lewis 2009; U]	only ventures into her fenced backyard an hour or so each day so her grandson can play. She is as suspicious of her non-Khmer neighbors as they are of her. She explained that, in Cambodia, she remembers her mother walking to visit other women, walking to the market, and feeling free to go into the forest to gather fruits. She is happy that she is in the United States but misses the ability to move about freely. She is acutely aware of a disconnection between her memories of place in Cambodia as connections across relationships and with the land and present-day discomfort with unfamiliar terrain and customs. “Here I have my grandson,” she continued, “I make my daughter and her family happy; I pray to Buddha. I am peaceful inside [the house] with my small family. Next time [in her next life] maybe I can be free, too.”<br>(p387) [Lewis 2009; U]
It Is Unusual to Plan for a Future When Mobility Might Be Limited.<br>We asked people if they had made plans for a time when they might not be so mobile or able to get out so easily (e.g., if they could no longer drive). Five participants were living with an adult child. One couple who had retired in the US from another country,assumed that at a certain point when they were frailer, they would move back to their home country for access to affordable support services. A Latina woman, widowed, originally from Nicaragua, mentioned a similar plan.(p.07) [Yen 2012; U]	I think I’m just going to rely on my daughters,or my granddaughter by that time. . . . I have a couple of girlfriends who live up here in the same situation I am, and, you know, we have talked about that, how maybe we can help each other drive or something. But, no, I have to admit I have not given it a lot of serious thought. (69, African American woman)(p.7-8) [Yen 2012; U]
The third discrepancy about the familial ideal and immigration reality the interviewees needed to work out was the generation gap. Mrs. Liu explained how she went to Chinatown everyday in order to break the isolation once her role as caregiver was less in demand by the children and then by her sick husband who eventually passed away: [Hsu 2014; C]	I had a monthly metro pass before, but the commute was still one hour one way from Ville St. Laurent to Chinatown. When it snowed, my daughter asked me not to go. She couldn’t stop me once she left home for work [laugh] …The application to Yi Kang [the semi private housing] needed a signature from the family. I begged her to endorse my application (Interview, 6 Jan 2009).

	Mrs. Liu then emphasised the generation gap in her choice of living alone: I am doing well here. I cook whatever I want. If I don't cook, I get food from downstairs...I am very satisfied with my life here. I am happy after living alone. Why? Young people cannot live with the elderly. For example, every Saturday they get up at 11AM. I get up at 5 or 6AM! I already had two meals by the time they are awake. Should I prepare their meals? I don't need to cook for them here (Interview, 6 Jan 2009). p.339 [Hsu 2014; C]
Mrs. Chen's story reinforced the idea that monolingual seniors had no fantasy about intergenerational cohabitation. She immigrated in 1987 to rejoin her parents who had been separated from the civil war since 1949. To ensure enough savings for her children's immigration in 1992, Mrs. Chen worked double shifts. Two years after her children's immigration, she was paralysed from the waist down in a car accident.<br><br>By the time of interview, her children had already left for career development. Mrs. Chen stayed in an apartment accessible by wheelchairs, a nurse came every morning for routine check-up; a live-in aid helped her with daily activities, and volunteers were requested from time to time to accompany her for medical appointments.Mrs. Chen supported the individual pursuit of wellbeing for each generation: career opportunities for her children and an easy access to Medicare and everyday requirements. Most importantly, she chose to stay alone without an emotional sense of sourness, abandonment or self-pity. [Hsu 2014; U]	When asked about living alone, she stated that "my daughter wants me to stay with her, but I prefer to stay here …I feel more grounded in Montreal. There is a security net here, and I can do lots of things alone …I don't want to bother them. Young people have their own lives. They don't want to abandon me, and I don't want to be their burden" (Interview, 15 Oct 2008) P.339 [Hsu 2014; U]
The desire to get out of the house was also influenced by their views on their current living situations. Some participants expressed a desire to socialise outside the home because it was too	Interviewer: And do you meet your friends often? AF07: Yes, I have many friends. In the past I played mah-jong twice a week at my home. Friends come to me to play mah-jong

much energy to host people at their own space: [Morgan 2021; U]	[which] could be very troublesome. Like some men need to use the toilet, they need to pass through my bedroom. After they left, I had to mop the floor, and I was really tired. Until in March this year I stopped playing it, not in my house anymore. [Morgan 2021; U]
Many of them commented on the 'togetherness' of the Turkish community in that everyone was connected with each other. It was also evident that they tended to use public space collectively, rather than going out alone. Some Turkish women, for example, reported that their husbands or other family members accompanied them when leaving the home, providing them with a sense of safety.(p.98) [Buffel 2013; U]	If I go out I'm usually with other people. I don't go out alone, so why would I be scared? (67-year-old Turkish woman, years in the neighborhood, Brabantwijk,Brussels(p.98) [Buffel 2013; U]
Analyses of the interviews with different migrant groups in the three English cities and Brussels also highlighted differences between men and women in terms of the use of public space. For example, older migrant men tended to have more informal gatherings with friends outdoors than migrant women.<br>The mosques, cafes or teahouses, where the men met each other, were described as 'male spaces' in the interviews – by male as well as female participants. In this respect, our study points at the role of Islamic prescriptions, stipulating that men and women keep sufficient physical distance in public.(p.100) [Buffel 2013; C]	A number of older migrant women in our studies, for example, avoided particular places in public space because they were afraid that the men would spread gossip about them and hence damage their reputations. For example, some women met to attend language courses or literacy programmes in community centres and did voluntary work such as cooking for students. Many Moroccan women also referred to a particular park, where they met other women and regularly took their grandchildren. They attached great importance to this park because it was seen as a 'female space', free of male control.(p.100) [Buffel 2013; C]
Some older migrants also create 'places of belonging' by participating in residents committees, community centres and religious associations. In general, we found that older women's participation in such activities tended to be rather limited, especially among Somali people. However, some women had important roles as volunteers in self-help organisations and community centres.(p.102) [Buffel 2013; U]	An older Moroccan woman in Brussels, for example, said:<br>I go to the community centre every day. I help with cooking and I'm involved in organising activities so that we can do things together . . . it's important to mix with people from different cultural backgrounds. (-year-old Moroccan woman, years in the neighbourhood, Old-Molenbeek, Brussels)(p.102) [Buffel 2013; U]

The men in this study talked about their engagement in religious activities organised by the mosque.(p.06) [Buffel 2017; U]	A 71-year-old man, said:<br>'Because I'm active in the mosque association, I know many people. I have connections with almost everyone around here. We organise language courses, trips, and activities for young people. I meet my friends in the mosque, we go for tea after or we meet each other in our homes.'(p.06) [Buffel 2017; U]
women's narratives about social support appeared to be centred on relatives, sick or vulnerable people in the community, and the looking after, or being looked after, by neighbours.(p.06) [Buffel 2017; U]	'I am very happy with my neighbours. They helped me a lot when I was having a difficult time after my husband died. May God reward them' (74-year-old woman).<br>'They [neighbours] are very important to us… Even in the smallest of matters one neighbour can help another […] "Komsu komsuya muhtaçtır" [loosely translated as: It's better to have a good neighbour than a distant friend]' (64-year-old woman).(p.06) [Buffel 2017; U]
Ethnographic observations demonstrated that Turkish older men tend to have more informal gatherings with friends outdoors than women, for example in the mosque or teahouses. During the interviews, there was a tendency among women as well as men to refer to these places as 'male spaces'. Teahouses in the neighbourhood were exclusively geared towards the need of Turkish male residents, offering opportunities to play games, gamble, watch Turkish football and socialise with other older men, who in most cases, originated from the same village of origin.(p.07) [Buffel 2017; U]	'I usually wake up quite early, I have breakfast, and then I go to see [his friends] in the Turkish teahouse around the corner… What do we do? We drink tea, just chat about general things… play tavla [backgammon]… And on Friday I go to the mosque. They [his friends] go as well.'(p.07) [Buffel 2017; U]
In contrast, the women in this study visited the mosque less frequently (in most cases only on important religious days), and often reported a lack of 'female spaces' or family-friendly places in the neighbourhood. [Buffel 2017; U]	'There are no activities around here, especially for the women. What we need here is a social space where we can take our grandchildren and have a chat with other women: A place for the women to get together.' (p.07)<br>Context: Islamic prescriptions, outlining that men and women should keep sufficient physical distance and should not meet in

	private, may further support 'male territoriality' in this neighbourhood, because women should not enter a space in which men are already present (Peleman, 2003: 159). The capacity to be 'mobile' in public space, both physically and socially, thus has a clear gender dimension. This was illustrated in our study through a comment made by an older Turkish woman in Brussels who avoided a particular place as she was worried that men would spread gossip about her, which could damage her reputation in the community: 'I only go shopping in the shop at the corner; I don't go to [the supermarket] because I have to pass that square then where all the men are' (63-year-old woman). [Buffel 2017; U]
In comparison with older migrant women, the men in this study also tended to have more informal gatherings with friends outdoors, for example in the mosque, cafes or teahouses; these regarded as "male spaces," both by some men and women. Islamic prescriptions,outlining that men and women should keep sufficient physical distance and should not meet in private, may further support such "male territoriality," since women should not enter a space in which men are already present (Peleman :). is was illustrated in our study through a comment made by an older Turkish woman in Brussels: (p.24) [Buffel 2011; U]	**"I only go shopping in the shop at the corner; I don't go to the Aldi [supermarket] because I have to pass that square then where all the men are." This woman was especially worried that these men, "who kept an eye on all passers-by," would spread gossip about her which could damage her reputation in the community, and therefore she avoided this particular place. [Buffel 2011; U]**
Many older Moroccan women also made reference to a particular park which they regularly visited with their grandchildren and where they met with other women. The park was seen as a place where they could escape from the social control of men. The importance attached to such "female spaces" was also evident in comments about a lack of meeting places in the neighbourhood. (p.25) [Buffel 2011; U]	A Pakistani woman in Manchester, for example, argued<br>"We need somewhere for the men to get together and a place for the women to get together. We need a lot more services and facilities for us elderly people here." [Buffel 2011; U]

4.3 Pre-migration neighbourhood experiences

Author Statement	Illustration
When asked to describe growing up in their native country, most participants started by talking about their country of origin and then quickly moved into com - paring it to their current residence in the United States. Participants continued throughout the interviews to draw on their past to describe the present and to bring together their meaning of ageing out of place and conceptualisation of home. In referring to their life in the country of origin, participants frequently referred to the "campo," meaning countryside, "campito," "pueblo," and "pueblito" (small town) to describe their place of origin. (p.04) [Curtin 2017; U]	**Ana Paula described her life in the Dominican Republic, "Life there is never the same as life here... I lived in a... "campo" where you know everyone in the world."<br>She continued, I came here when I was 35 years old .... My daughter be- came a citizen and requested me. After arriving here, well, you already know how life here is. Well here in many ways, one lives a life of 'having it all.' And at the same time, with yourself, you have nothing." She further explained, "Over there, in your own country, you don't feel loneliness like you feel it here. Over there in the 'campo' you open your door in the morning and people say 'hi, how are you?' And that's a different life. Not here. If it's cold here you have to live in doors. There are moments in your life when you feel sad and anxious because of the loneliness. I have all my grandchildren here but everyone lives their own life. Everyone has their own obligations. It's not like if I get sick they'll say 'I'll take care of you grandma, because they can't. You understand? That's why I call it (U.S.A.) the country of loneliness." (p.04)**<br><br>Similarly, others described their sense of place as the community, the farm or where they slept. Gabriela described how she "was born and raised in a small 'campito'... I remember that was back when I was young, like five years old. We all grew up together and all slept in the same bed." Santiago stated, "Oh it was on the farm, the first years of my life were in the 'campo.' Working the

	farm life, feeding the chickens and milking the cows, then taking the milk out to the main road to sell." Camila described her place of origin as a small pueblo where she worked with her mother and father.. (p.04) [Curtin 2017; U]
In describing their experiences in their native country, although they enjoyed the communal aspects of their towns and villages, the majority of participants described growing up in some level of poverty. Despite living in some degree of poverty, most participants spoke of having good memories of their childhood, largely due to close-knit, loving families. (p.04-05) [Curtin 2017; U]	As Gabriela described it, "My parents were very poor. We were six sisters and we all slept in the same bed because we were so poor… the bed was made out of wood because in those days we had no money." She continued, We use to study in a small school in town, but my father couldn't even afford to get us a notebook…. Since the very young age of nine I was babysitting young children, cleaning kitchens and worked as a servant"(p.05).<br>As Alejandro described, "It was a poor household… but full of love and quality when it came to orientation especially in the spiritual aspect, my mother was a very spiritual lady. She showed me the moral values of human beings." (p.05) [Curtin 2017; U]
Approximately one third of the participants at the time of the interview were living alone and deliberately described two different types of homes, one where the family lived and one where they lived alone.(p.05) [Curtin 2017; U]	Some of the participants expressed a sense of loneliness as in the passage earlier from Ana Paula. Martina also related, "I try to make the best of what I have here, that also means forgetting of what I used to have in Guatemala. I already got used to this. I find it nice and comfortable now."<br>Lucia described how in Columbia she always lived with her family, but here, Here I have lived in loneliness, at all hours. I believe that here it has not been a home. First I had my apartment and I lived in it with my daughter. I said it was my 'house', never really my 'home,' yes my house".(p.05) [Curtin 2017; U]
…other residential strategies have arisen because of circumstances surrounding life in the United States. There is a shift from multigenerational housing to clustering in neighborhoods or purchasing adjacent	One elderly couple described how first their oldest son had purchased a house, then a son-in-law purchased the adjacent house, next a niece and her family bought a house behind the rest. After a few years, the entire extended

houses…Although the climate in the United States is too mild for the plants to make fruit, they provide a visual reminder of the landscape of Cambodia and a reduction in feelings of aging out of place. Her feelings of comfort, connection, and satisfaction occurred once she and her family devised a way to create an environment that felt more familiar, more like Cambodia and has allowed the entire extended family to insulate themselves through an ecological system of physical and socioemotional space. (p388) [Lewis 2009; U]	family could travel from house to house without leaving the common yard. The wife explained, "We come here [to the United States] with so little . . . one house, then another house . . . we build our own village. My grandchildren . . . I hear playing and I feel comfort." She and her family also have planted banana plants along the fence that surrounds their family compound. (p388) [Lewis 2009; U]
several ageing migrants felt emotionally attached to both their present place of residence and their place of origin, reflecting the ambivalent nature of their sense of home and place. (p.09) [Buffel 2017; U]	'I feel like this [neighbourhood in Brussels] is my home now, but I also miss the mountains and scenery in my home in Turkey' (71-year old woman). (p10)<br>'I feel very much attached to my neighbourhood. Whenever I go to Turkey I miss my friends and children [who live in Belgium]. But when I'm here, I miss my family in Turkey' (64-year-old man).(p09) [Buffel 2017; U]
Evidence of such "de-territorialisation" of belonging and attachment was found in the way a Somali woman in Liverpool and a Moroccan woman in Brussels talked about their "first" and "second home"; and in the local and transnational connections about which many interviewees spoke. The following excerpts illustrate how several older migrants felt emotionally attached to both their present place of residence and their place of origin, reflecting the ambivalent nature of their sense of home and place. (p.27) [Buffel 2011; U]	I feel very much attached to my neighbourhood. Whenever I go to Turkey I miss my friends and children [who live in Belgium]. But when I'm here, I miss my family in Turkey (Turkish man, Brussels).<br>is [neighbourhood in Manchester] is my home now. I don't think of it as anything else. But there is nothing like your home in Pakistan, which I miss. But this is my home (Pakistani man, Manchester). [Buffel 2011; U]
Additional participants noted how the lakes and parks reminded them of home, their place of origin. [Hawkins 2022; U]	Participant 15 (61 years old) said: "I love the beach...I am from Puerto Rico, surrounded by water...I love the water. Here I love to go to the water, the water by Downtown." Participant 15 selected a picture of the beach in Puerto Rico (photo five). Participant 21 (80 years old) took photo six: "I like where we live. Because it's very tranquil, very beautiful.

	Not many cars pass by, it's good, good, very beautiful." As you can see in her photo, there a few cars, ample walking area, and plenty of green (in the summer, the photo was taken in February)" (p. 111) [Hawkins 2022; U]
Outside of the temple, participants' socialization with ethnic peers was limited. Friendships forged at ethnic spaces were maintained primarily via telephone due to geographical dispersion and lack of independent and accessible transportation. Apart from the temple, participants infrequently met their ethnic peers at family dinners, private almsgiving events, and cultural shows. Although infrequent, these meetings were reminiscent of their lives in Sri Lanka. [Wijekoon 2018; C]	**Now when we go to the homes of the Sri Lankan families that live here, we all get together and sing Sinhala songs. I enjoy that very much. I am overcome with the feeling that I am back in Sri Lanka when I sing those songs and speak to them [in Sinhala]. There we are all the same. There we act in our [Sinhala] way. – Apsara" (p. 155) [Wijekoon 2018; C]**
Furthermore, in an automobile dependent culture, these suburbanite non-driving participants felt confined and excluded from broader society. (p. 164) [Wijekoon 2018; U]	...now when you enter your home, all the doors and windows are closed due to the cold. Once you enter the home, you are isolated. You cannot see your surroundings. If you look at the road, you rarely see any people walking. Now in Sri Lanka if I happened to step out from the yard, go out to the road and walk along the road, I see people. There are people everywhere, crowds everywhere. [I see] people talking, making jokes, screaming and shouting and engaging in various types of discussions. I would see familiar people, [they] will ask 'where are you going?', 'how are you doing?'. Worries and concerns within my mind would vanish. So, I did not feel lonely or isolated there. That is what I felt after coming here [to Canada] after spending 75 years [in Sri Lanka]. I couldn't bear it in the least. I initially wondered whether I could tolerate it. – Bandula [Wijekoon 2018; U]
Outside of their child's home, participants rarely engaged in their neighborhoods and communities. Sponsoring children shouldered the financial care and fulfilled community occupations on participants' behalf. Thus,	I lived as a very sociable person there [in Sri Lanka]. [I had] a lot of connections. I would go to gathering places to engage in convivial socialization. I do not have the opportunity to continue that level of socialization here. I

outside of healthcare facilities, the Buddhist temple, and infrequent trips to a mall, post-office, or library, participants rarely engaged in their community. The lack of diversity of occupational spaces meant that participants social encounters were limited to their families, members of the ethnic community, healthcare providers, service providers (e.g., cashiers, pharmacists) and the occasional telemarketer. The limited interactions with the non-ethnic community did not meet the socialization needs of some participants, particularly males, who had previously enjoyed an extensive social life in Sri Lanka. [Wijekoon 2018; U]	grieve that. It is during the winter that I feel very restricted, alone and isolated. I cannot stay in one place like this. – Bandula" (p. 165) [Wijekoon 2018; U]
In Canada, participants were deprived of the informal daily social interactions and pleasantries that took place between neighbors in their home country. In describing this lack of regular interactions, which did not provide sufficient opportunities to 'use' his mouth, Saman stated "I have cobwebs in my mouth from not talking". Specifically, Canadian culture was perceived to promote privacy and social distance between persons in society. Visual markers such as closed doors of neighbours' homes, in contrast to the open-door policy of Sri Lanka, led to participants' perceptions of their Canadian neighbours as uncongenial, unconcerned, and unwelcoming. These perceptions reinforced participants' sense of exclusion in their neighbourhoods and communities…<br>The social distance and privacy of Canadian culture engendered a feeling of anonymity and invisibility within their neighborhoods." (p. 166) [Wijekoon 2018; U]	In Sri Lanka, we spoke to all our neighbours. They come over [to our house], we go over [to their house]. That does not exist here. A neighbor does not know whether there is an ill person next door, whether there is an argument, or whether there is an assault. What happens in this house is not known by the neighbor next door. That is the nature of this country. As I said before, the culture of Sri Lanka operates according to a certain method. You are encouraged to give a helping hand to anyone in your vicinity. There is no such thing in this country. If there is a person on the street, they do not even look you in the eye. At least in Sri Lanka, if you speak to someone they would say 'ok bye'. There is no concern for your neighbor, whether it is genuine or insincere. Because of that we do not have a relationship [with the neighbours]. – Ranil (p. 166) [Wijekoon 2018; U]
Among immigrant older adults, walking was identified as a popular way of getting around in their respective countries of origin. However, participants walked less in their current community in the U.S. due to the lack of sidewalks, winter conditions, unsafe neighborhoods, and heavy traffic. Walkability	They say because the winter was always good (in Somalia) they used to walk. They used to walk for local stuff that they can go. They say walking was healthy for us. It was good, but here we cannot walk. (Somali). (p. 8) [Dabelko-Schoeny 2021; U]

of neighborhoods varied by geographic region. [Dabelko-Schoeny 2021; U]	
Issues of accessibility and personal preferences regarding activities complicated the relationship between green spaces and well-being. For instance, older Chinese immigrants preferred green spaces that supported their ability to partake in Chinese activities. [Gao 2020; C]	On the Gold Coast, all (100%) participants reported that they still retained a willingness to continue their previous lifestyle and physical activities in green spaces, such as performing Tai Chi, square dancing, and singing. Gu (male, 63 years old, living in Australia for 2 years) stated his experience: "I like performing Tai Chi. I practiced Tai Chi for nearly 10 years before I moved to Australia. Now, I've joined a Tai Chi club, and I can continue to practice it." (p. 6) [Gao 2020; C]
Because of the differences between the green spaces in China and in Australia, the participants fulfill their values of being active and healthy on the Gold Coast to a lesser degree, which has generated negative perceptions of green spaces as older Chinese immigrants are unable to continue the lifestyle that they had in China. [Gao 2020; U]	Ping (female, 65 years old, living in Australia for 7 years) made the following comparison: "In my hometown, there are always various activities, such as square dancing and choir singing. I can participate in these activities. I always want to find similar activities here, but it's difficult. I only walk here, nothing else." (p. 8) [Gao 2020; U]
They prioritized going to green spaces as a regular daily activity to spend leisure time and escape boredom. They perceived experiences with green spaces to be integral in experiencing a fulfilling day. [Gao 2020; U]	As Fu noted: "I go to the small park to walk and do some exercise twice a day. Once is in the morning and the other is after my dinner. In China, I have lots of physical or social activities, but in Australia I have no place to go. So, going to the nearby park has become a thing for me; otherwise, I have nothing to do and I am always at home." (p. 8) [Gao 2020; U]
They perceived that the monotonous landscape of green space could not fully meet their values of being involved in an active environment [Gao 2020; U]	as Tian (female, 68 years old, living in Australia for 4 years) noted: "Although the environment is good, it is boring. There is no other scenery I can see. The trees, flowers, and large lawn are always the same. You can see a few people walking or running. I feel bored." (p. 9) [Gao 2020; U]
In Romania, Gizella took care of her house and did some handcrafts for herself and her family. She explained that she experienced a big difference between those two countries,	"Not like in my hometown. There we got out of the house and we took some steps and arrived. Or the market was only two or three streets away." "The people here are very

mainly the distances between her house and supermarkets and the social contact [Schuster 2019; U]	reserved. They aren't like us in [hometown]. Because our neighbours walk into your house and opens up the pot and looks at your food [laughs]." (p. 64) [Schuster 2019; U]
Oana's health also did not allow her to visit Romania. She missed her friends from Romania the most and explained that the public transportation system in her current neighbourhood was insufficient in order for her to make and visit friends in Canada. Not having a driver's license, nor a car, made it difficult for her to get around the city independently. [Schuster 2019; U]	"Here, it is very difficult to have friends, because it is impossible to reach them. I don't have a driver's license and I depend on my daughter to drive me." "It takes me more than an hour to get to the first bus station. And there, if I go, I have to change the bus. [...] If I go somewhere, it would take me the whole day." (p. 68) [Schuster 2019; U]
all four participants reported having trouble finding and connecting with people in Canada due to a perception of Canadian society as individualistic and reserved, as well as the physical distances between neighbourhoods. [Schuster 2019; U]	"I know some people from London. Even Cuban people. But they don't live really, very near of me, so... it's not easy for me to visit them." (Samantha)<br>"What should I say... the people here [in Canada] are very reserved. They aren't like us. [...] We don't visit our neighbours [in Canada]. [...] I don't have any contact with strangers." (Gizella) "Here it is very difficult to have friends because it is impossible to reach them. [...] Here [in Canada], everybody stays in their family. They [Canadians] are more individualistic." (Oana)" (p. 79) [Schuster 2019; U]
Limitations, such as an inability to drive, has caused a shift toward elders being homebound, engaging in temple participation solely on weekends, and infrequent trips to visit family, friends, or markets because of the need to rely on younger family members who often are working….As her narrative shows, it is more difficult for elders to achieve the level of freedom they expect. Elders, such as the woman described above, limit their interactions with non-Khmers because of language difficulties and what they perceive as suspicious looks from neighbors. The	One older woman, living in a small house with her daughter while caring for her young grandson, described how she had lost her freedom when she arrived in the United States. No longer able to walk to the temple, to markets, or to visit other elderly women, she feels trapped. She explained, "I am afraid to walk around my neighborhood. I don't know the people; they only speak English. They watch me when I walk so I am afraid." She stays inside her small frame home and only ventures into her fenced backyard an hour or so each day so her grandson can play. She is as suspicious of her non-Khmer

critical aspect of neighborliness and belonging (Casarett, 1991) is missing.<br>(p387) [Lewis 2009; U]	neighbors as they are of her. She explained that, in Cambodia, she remembers her mother walking to visit other women, walking to the market, and feeling free to go into the forest to gather fruits. She is happy that she is in the United States but misses the ability to move about freely. She is acutely aware of a disconnection between her memories of place in Cambodia as connections across relationships and with the land and present-day discomfort with unfamiliar terrain and customs. "Here I have my grandson," she continued, "I make my daughter and her family happy; I pray to Buddha. I am peaceful inside [the house] with my small family. Next time [in her next life] maybe I can be free, too."<br>(p387) [Lewis 2009; U]
Finding a place called home, based on the experiences of these Chinese seniors, appeared to have a lot to do with the weather, the natural and built environment, and the standard of living.<br>Similar to earlier studies weather is found to be an important factor in old age. Bad weather or winter seasons can be major confining factors for older adults.. This study also reveals that some seniors moved to the south for the warm weather to avoid cold winters like in Boston. In addition, seniors in this study echoed findings in earlier studies that emphasized positive emotions such as comfort, joy, and relaxation in their perceptions of making Atlanta their home. Clearly, the natural and built environment is directly linked to individual's emotional perception of the home. [Zhan 2017; U]	Beyond the warm weather, the natural built environment is another reason that adds to the Chinese seniors' sense of home. Ms. Qin described her sense of home in this way: "When I go out, I go inside the park, children's playing ground makes me very happy. I take a walk every day and enjoy the sunshine, which makes me feel good. Also, the library–when I was in better health, I used to borrow every kind of books to read. I also borrowed foreign DVD (meaning non-Chinese). So people in the library were very nice to me. There were Chinese books there too." [Zhan 2017; U]
Participants discussed their love of both wild animals in parks and their own pets as positive experiences in their lives. Animals, both wild and domestic, provided a sense of both enjoyment and comfort to participants.<br>[Hawkins 2022; U]	"Photos three and four were taken by participant 8 (71 years old). She reiterated her love of the lake and parks particularly her daily walks in those parks, as well as her enjoyment of the animals…<br>"I really like this beauty...When I'm going home [to my apartment], this is [the beauty] I

	see [showing the photos]... I love them (the birds), I don't know why... Once I'm back home, I'll show my neighbors."– Participant 8 (71 years old). Participant 8 also noted that seeing the animals reminded her of home: "here there are plenty (of animals), there (in Azerbaijan) there are a lot (of animals)."– Participant 8" (, p. 110) [Hawkins 2022; U]
A few Turkish people in Brussels expressed a similar sense of discouragement because they didn't feel integrated in their community. (p.25) [Buffel 2011; U]	A woman who migrated from a different village than the majority of Turkish people in her neighbourhood, reported how she felt excluded from the Turkish community:<br>"I would like to get to know other Turkish people, but it's difficult. Everyone knows everyone here… It's difficult to find acceptance if you're not from Kayseri." Further in the interview, she said: "I became somehow isolated; that's why I'm stuck in the house now and don't see people… nobody helps me, no one cares about me." [Buffel 2011; U]

Lack of Availability for Senior Housing in Desired Neighbourhoods

There is a lack of affordable independent older adult housing in the neighbourhoods that older immigrants are wanting to live in.

Author Statement	Illustration
However, getting a senior public housing, especially the ones located in Chinatowns, was very difficult and there was a long waiting period. (p.385) [Chen 2022; U]	I don't have a house and have been applying for government housing for years. I use all my cash benefits to rent a room, and I apply for food stamps because I don't have enough money. (P5, Female, 70) (p.385) [Chen 2022; U]
As new immigrants were not eligible for some public benefits, their aging arrangement was more passive. They had to depend on their	It is a bit hard to think about a better condition due to my short immigration period. I expect more accessible access to senior housing and

families, especially adult children since they had fewer options economically and socially as they migrated (p.385) [Chen 2022; C]	home care service. I will be satisfied if I had met these needs. (P17, Male,77) (p.385) [Chen 2022; C]
Monolingual seniors found Chinatown attractive not only for convenience and autonomy but also for sociability and daily normalcy. This further required them to negotiate the space of everyday life in Chinatown. The grandiose Chinese arches might be important for tourist development or urban diplomacy with Shanghai, but They played a trivial role in Chinatown residents' life. In contrast, social and exercise space in Chinatown was important for them to cultivate a sense of belonging, social relations and individual wellbeing P.339.<br>In addition to public or semi-public areas, the respondents in this research also took advantage of common areas in subsidised housing complexes and Chinese organisations. They socialised, took language or exercise classes and participated in leisure activities such as Mahjong, Tai chi, choirs and Chinese opera, among others. P.340 [Hsu 2014; U]	Despite the accelerated development for tourism, Montreal's Chinatown continues to provide a vibrant social life for seniors. A long waiting list of 4 years on average did not prevent monolingual seniors from applying for a subsidized one or two-bedroom apartment in one of the five Chinese-run housing complexes. Construction began during the 1980s to accommodate the rising amount of monolingual single senior dwellers. About 400 units reduced the number of seniors living in deplorable rooming conditions. Nonetheless, these seniors did not confine themselves to their apartments. It is common to see Chinese seniors using hallways or food courts in nearby public buildings and shopping malls for socialisation. They gather together to chat, to play Chinese chess or simply to sit alone reading community newspapers. It is<br>equally common for them to spend a whole day in the YMCA in the Guy Favreau building. While some fitness classes are specifically designed and instructed in Chinese, Ping Pong and badminton courts are popular among older immigrants.<br><br>Mrs. Yip recounted her everyday life:<br>I can walk to every corner in Chinatown. Friends or relatives take me out of town for BBQ or to the countryside on the weekends…I go to the YMCA every day. Tuesday and Thursday I study [French] at the elderly club. I also stay there for extra two hours or so just to chat…I volunteer in the Chinese hospital, as I can chat with those who have nobody to talk to in Teochewnese (Interview, 13 Dec 2008).P.340<br>Chinatown was mapped out by Mrs. Yip according to sociability and peer support.Her narrative also singled out how comfort and a general sense of satisfaction were negotiated

	out of the paradox of autonomy and dependence. Even though she did not confine everyday life to the ethnic quarter, her autonomy from family and children nonetheless depended on ethnic functions in the small ethnic community, ranging from the convenience of grocery shopping, the ease of mobility, the availability of peer support, to meaningful habitual practices. [Hsu 2014; U]
Lack of affordable rental housing for seniors in Vancouver [Brotman 2017; U]	“The hardest part about living in Canada is finding a rental house” This picture of a potential rental home was taken by Mr. Chul through the rainy window of a car rented by his visiting niece in his ongoing search for affordable housing. Kim Young Chul and his wife live in his daughter's home, which she hopes to sell now that she has moved to the U.S. It is distant from the services they use; they sometimes need to travel 2 hours each way to reach them. Young Chul's wife has osteoarthritis which limits her mobility and he is seeing-impaired so cannot drive. In the absence of their daughter, they are relatively isolated. They relied on their daughter extensively for interpretation and navigation when they arrived because they were busy running their own business; as a result they did not learn English. Now they are struggling to find affordable rental accommodation. Language, lack of transportation to view housing options, a limited income, and a lack of knowledge of available financial supports and navigation assistance are huge impediments. Young Chul looked into a Korean-run seniors’ care facility, but told us through the interpreter, “First of all, it’s very expensive. It costs a lot. For two like $3300, it’s a lot. He thought they are pretty healthy and they still have manageable life. So they don’t need to go in right now but maybe later.” (p. 63)<br>“His daughter’s marriage to a fellow Korean did not last and, in 2014, she re-married, this time to an American, and moved to the United States. Young Chul and his wife tried but did

	not feel comfortable living with her in the U.S. yet he feels he cannot return to Korea since he lost his status and family ties when he left. Life in Canada without his daughter has been very difficult he and his wife were so reliant on her. They feel isolated due to their geographic location, medical appointments and community centers are almost two hours away by public transit, and few services are tailored to Koreans. Their lack of English is challenging and has motivated him to study it at the library." (p. 48) [Brotman 2017; U]
Some interviewees indicated that they would like to live in senior housing, where they believed they would receive advanced support for older adults. However, they felt that staying in their neighbourhoods was essential….<br>Participants indicated that norms, cultural aspects, and traditional cuisine were important considerations contributing to their preferences, as was the need to move to another neighbourhood (with consequences for social contacts) due to the unavailability of housing specifically for older Surinamese adults in some interviewees' neighbourhoods. (p. 8) [Jagroep 2023; U]	They noted that senior housing in their neighbourhoods was fully occupied, with long waiting lists: You are 80 years old and you have to climb the stairs four high with the groceries. There are a lot of older adults who have to use stairs, which is an obstacle for them. One of the complaints that you have as you get older is knee problems. So, when they do not have an elevator, they do not want to go down and back up. There Are senior residences in the area, but they are all occupied. (Participant 14)<br>The interviewees further indicated that housing specifically for older adults with Surinamese backgrounds was present in their neighbour hoods. In addition, Hindustani Surinamese interviewees reported the availability of group living for Hindustani Surinamese in their neigh bourhoods. [Jagroep 2023; U]
